# A platform of ADAPTive scaffolds: development of CDR-H3 β-hairpin mimics into covalent inhibitors of the PD1/PDL1 immune checkpoint†

**DOI:** 10.1039/d4cb00174e

**Published:** 2024-11-04

**Authors:** Sarah H. Naylon, Alexis D. Richaud, Guangkuan Zhao, Linda Bui, Craig P. Dufresne, Chunjing J. Wu, Medhi Wangpaichitr, Niramol Savaraj, Stéphane P. Roche

**Affiliations:** a Department of Chemistry and Biochemistry, Florida Atlantic University Boca Raton Florida 33431 USA sroche2@fau.edu; b Thermo Fisher Scientific West Palm Beach Florida 33407 USA; c University of Miami, Miller School of Medicine Miami Florida 33136 USA

## Abstract

Aberrant and dysregulated protein–protein interactions (PPIs) drive a significant number of human diseases, which is why they represent a major class of targets in drug discovery. Although a number of high-affinity antibody-based drugs have emerged in this therapeutic space, the discovery of smaller PPI inhibitors is lagging far behind, underscoring the need for novel scaffold modalities. To bridge this gap, we introduce a biomimetic platform technology – adaptive design of antibody paratopes into therapeutics (*ADAPT*) – that enables the paratope-forming binding loops of antibodies to be crafted into large β-hairpin scaffolds (*ADAPTins*). In this study, we describe a novel strategy for engineering native CDR-H3 “hot loops” with varying sequences, lengths, and rigidity into *ADAPTins*, ultimately transforming these compounds into irreversible covalent inhibitors. A proof-of-concept was established by creating a series of *ADAPTin* blockers of the PD1:PDL1 immune checkpoint PPI (blocking activity EC_50_ < 0.3 μM) which were subsequently modified into potent covalent PD1 inhibitors. The compelling rate of stable and folded *ADAPTins* above physiological temperature (21 out of 29) obtained across six different scaffolds suggests that the platform technology could provide a novel opportunity for high-quality peptide display and biological screening.

## Abstract 2

This study presents a biomimetic platform technology (*ADAPT*) that enables the display of antibodies CDR-H3 loops into stand-alone β-hairpin peptide scaffolds to design new classes of sizeable PPI inhibitors.

## Graphical abstract

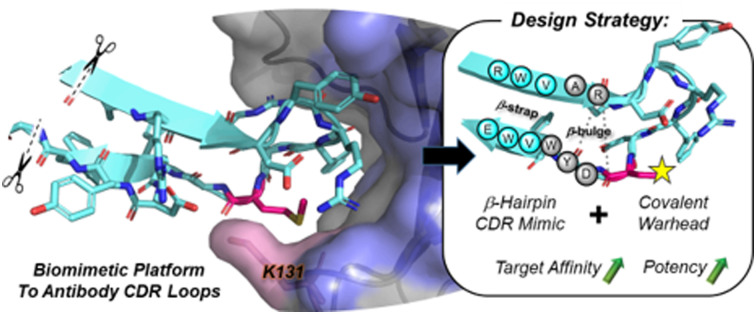

## A. Introduction

Protein–protein interactions (PPIs) regulate a plethora of fundamental cellular processes and their misregulation has now been associated with a variety of diseases.^1–4^ Yet, most PPIs interfaces exhibit rather shallow, water-exposed, and sizeable surface areas (800–2000 Å^2^) which are challenging to disrupt with small molecules from conventional drug libraries (*M*_W_ < 0.5 kDa, binding surface < 100 Å^2^).^5,6^ Moreover, theses interfaces can be either rugged or more dynamic, further contributing to their “undruggable” reputation. Over the past two decades, a new landscape of antibody drugs (Abs) and biological therapeutics of smaller size such as nanobobies,^7^ DARPins,^8^ and more recently BiTEs^9^ have truly revolutionized our clinical approach to targeted therapies.^10^ Despite their efficacy in modulating or blocking PPIs, the massive size of Abs (∼150 kDa) is often associated with delicate pharmacokinetic properties such as low bioavailability, but also poor tissue penetration, and slow clearance rates resulting in undesirable high systemic accumulations.^11,12^ Because of these drawbacks, a relatively unexplored therapeutic space between large biologics and low-molecular weight small-molecule drugs has attracted a lot of attention.^13–15^ In this uncharted space, cyclic peptides,^16^ bicycles,^17^ β-bracelets,^18^ and other helical peptides^19–21^ have laid the groundwork for the development of smaller size scaffolds as PPI inhibitors.^22^ Despite these advances, a pressing need persists for more robust and versatile scaffolding technologies capable of engineering peptide therapeutics with antibody-like structures, affinity, and potency.^23^ With this goal in mind, we created the *ADAPT* technology (short for adaptive design of antibody paratopes into therapeutics) that enables “hot loops” of antibodies with varying sequences, lengths, and rigidity to be crafted into short stand-alone β-hairpin scaffolds (*ADAPTins*).

**Fig. 1:**
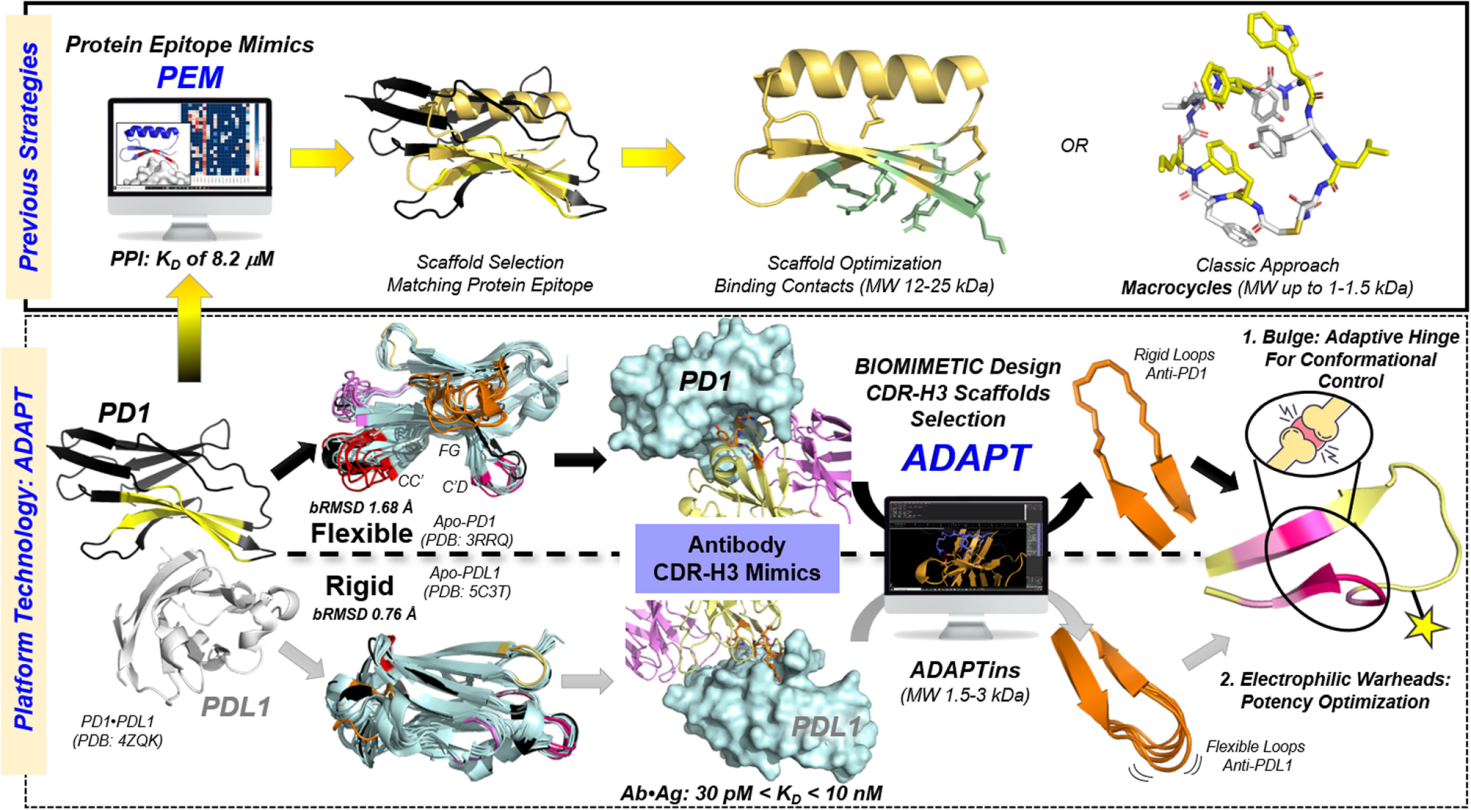
*ADAPT* platform technology. A biomimetic approach for designing β-hairpin peptide inhibitors of PPIs (*ADAPTins*) based on the plasticity or rigidity of the protein of interest. The relative flexibility of PD1 (*b*RMSD of 1.68 Å) compared to its ligand PDL1 (*b*RMSD of 0.76 Å) was calculated from backbone alignments over +400 atoms using unbounded *apo*-PD1 (PDB: 3RRQ) and *apo*-PDL1 (PDB: 5C3T) as respective reference. Top panel depicts typical strategies for designing protein epitope mimics into peptide macrocycles or larger protein-derived scaffolds. Bottom panel depicts a novel general approach to mimic CDR-H3s found in antibodies paratopes into *ADAPTin* scaffolds of varying rigidity. These stand-alone scaffolds (β-strap + β-bulge motifs) can display a broad variety of CDR-H3 loops which can be modified to incorporate electrophilic warheads to covalently bind a protein target.

To substantiate the technological proof of concept, we selected the programmed cell death-1 protein (PD1) and its ligand-1 (PDL1) as our focal PPI target. Here, we outline a general strategy to engineer synthetic loop mimics into *ADAPTins* that mimic the native fold of antibody CDR-H3 loops. We showed that out of the six anti-PD1 antibodies evaluated, four distinct CDR-H3 scaffolds could be obtained without altering the original H3 loop sequence. Several standalone CDR-H3 mimics displayed a remarkably efficient inhibition of the PD1/PDL1 immune checkpoint interactions at sub-micromolar concentrations. Selected *ADAPTins* were subsequently crafted with electrophilic warheads to achieve a covalent and irreversible inhibition of PD1 and advance candidates for *in vitro*- and *in cellulo*-studies. Unlike conventional strategies of protein epitope mimicry (Fig. 1, Top panel), our technology offers a novel avenue for grafting large non-canonical CDR-H3 antibody loops into smaller *ADAPTin* scaffolds which are not accessible by other means (Fig. 1, bottom panel).

## B. Results and discussion

### General design principles guiding CDR-H3 mimics

Despite the significant breakthrough by Baker and Craik in transforming computational models of protein motifs into large scaffolds (*M*_W_ ∼ 12–25 kDa),^24^ the synthesis of sizeable and 3-dimensionally folded peptides outside of their protein context remains non-trivial.^25^ Although powerful, protein epitope mimics (PEMs) are inherently limited to canonical motifs found in proteins (Fig. 1, top panel).^26,27^ Similarly, the miniaturization of high-affinity antibody paratopes into smaller scaffolds, *aka* complementary determining regions (CDRs), has been essentially focused on the canonical loops found at the apexes of light-chain (L1-3) and heavy-chain (H1-2) CDRs.^28,29^ Yet, recent structural analyses of protein–protein complexes in the Protein Data Bank revealed that a large number of PPI “hot contacts” are in fact generated by nonregular secondary structures (∼50%) mainly from loops embedded in either β-hairpin structures or non-canonical forms.^30,31^ To bridge this gap, our approach innovatively repurposes the CDR-H3 hairpin and β-bulge motif into a unified scaffold that could withhold longer loops (>10-residue long) while closely mimicking the native fold found in high-affinity antibodies (Fig. 1, bottom panel).^32^

In comparison to all other CDRs, CDR-H3 loops are known to possess the largest variability of sequence^33,34^ topology, and length (4 up to >21 residues)^35^ which drastically increases the span of conformational space accessible^34,36^ to maximize protein binding affinity and specificity.^37–40^ Strikingly, the vast majority of CDR-H3s possess a β-bulge motif edging their loops;^41,42^ Yet the role of this structural motif in CDR-H3 folding, stability, and rigidification remains mostly unknown.^43^ One could imagine that the promiscuity of bulges is a result of evolutionary optimization to favor the display of long and conformationally adaptable H3 loops to mutations and 3D-rigidification (Fig. 1, bottom panel conformational hinge).^44–46^ In addition, most H3 loops have unique ‘noncanonical’ topologies^47,48^ that may enhance antibody specificity to a protein target and therefore constitute an exciting starting point for the design of PPI inhibitors.^49^ For all these reasons, we and others became interested in mimicking CDR-H3 scaffolds to recreate miniaturized peptide loop displays either for protein loop grafting or standalone loop scaffolding.^32,50–53^

To validate a proof of concept of biomimetic CDR-H3 scaffolding platform, we selected the immune checkpoint PD1:PDL1 interaction. Indeed, anti-PD(L)1 antibody drugs have completely transformed our current approach to cancer therapy.^54^ The PD1:PDL1 interaction is nearly an ideal model to test our *ADAPT* technology because: (a) PD1 is inherently more flexible than PDL1 (backbone *b*RMSD of 1.68 Å *vs.* 0.76 Å) which is in line with an entropically-driven induced-fit binding mechanism of PD1 to PDL1 *vs.* PDL2,^55^ and (b) its low complex affinity between PD1 and PDL1 (*K*_D_ of 8.2 μM) suggesting that a PEM strategy would be complicated. Indeed, the PD1·PDL1 interface lacks well-defined binding pockets and is highly dynamic, which explains why small-molecule intervention remains challenging. Since the initial report by Bristol-Myers Squibb scientists of anti-PDL1 macrocyclic peptides,^56^ only a handful of small molecules^57^ and larger peptide scaffolds have been discovered (Fig. 1, top panel).^58,59^ Likewise, only very few PD1 antagonists have been reported.^60,61^ Excitingly, a significant number of high-resolution crystal structures of PD1·AbDrug complexes are available, providing us with detailed structural information to rationally design anti-PD1 CDR-H3 mimics (*ADAPTins*) (Fig. 1, bottom panel).

**Fig. 2:**
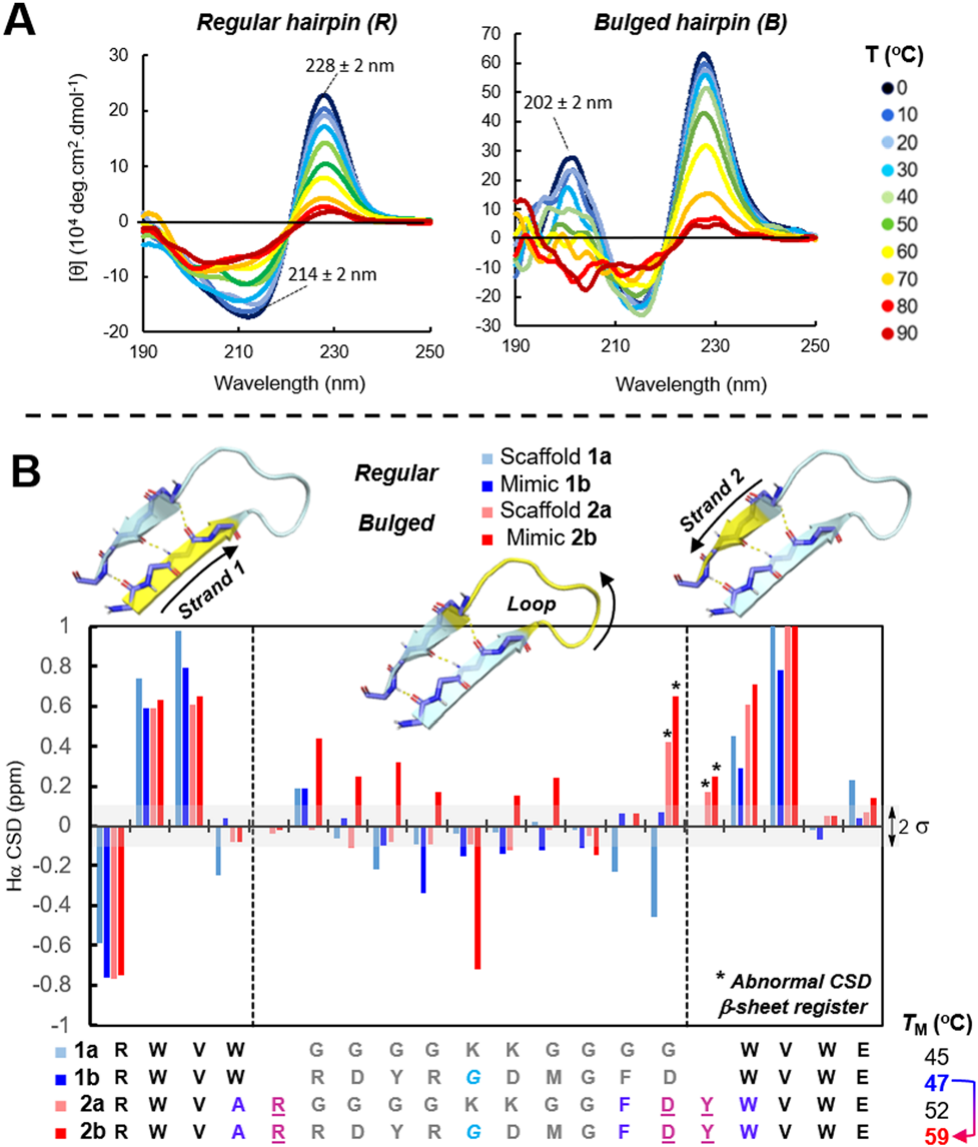
Structuration of stand-alone CDR-H3 scaffolds and their loops. Regular 1*vs.* bulge-like 2 β-hairpins. (A) Variable-temperature CD analysis of hairpin folds. (B) H_α_ NMR chemical shift deviations (CSDs) analysis with 2*σ* of standard deviation from random coil values (in shaded grey).

Recently, our laboratory brought forward a novel synthetic technology for the synthesis of acyclic β-hairpins with long loops. Prior to these studies, the access to hairpin peptides was essentially limited to short loops possessing an innate β-turn (4-AA long: D̲DATKT̲ and N̲PATGK̲).^62–64^ Inspired by the work of Andersen on long-loop closure,^65–67^ we created a series of minimalist β-straps (strap = strand + cap) RWVW⋯W(V/H)WE that enable regular hairpin folds with up to 10-AA loops. To compare hairpins’ stability, both regular (R) and bulged (B) scaffolds were crafted around a flexible 10-residue model loop (G_4_K_2_G_4_) and analyzed by CD (circular dichroism) spectroscopy (Fig. 2(A), see ESI,† Table S5). The tertiary structure of these model scaffolds 1a*vs.*2a in solution were recorded in the far-UV CD spectra. The characteristic and very intense exciton couplet maxima at 214 and 228 ± 2 nm in the CD spectra of β-hairpins (π–π* transition) originates from an edge-to-face staking of tryptophans W2/W19 nearby the C-/N-β-strap termini. The CD-exciton intensity was therefore used as a global probe to determine the %-folding of *ADAPTins* and to obtain melting curves corresponding to the hairpin fraying mechanism upon a gradual increase of temperature (0 to 95 °C).^68^ The melting temperature (*T*_M_) upshift of about 10 °C calculated from CD-melts clearly indicates that the bulged scaffold 2a is significantly more resistant to thermal denaturation than the regular hairpin 1a. As shown in the CD-spectrum of the bulged scaffold 2a (Fig. 2(A)), a unique positive band at 202 ± 2 nm was observed in each bulged *ADAPTin*2 which was never observed in the spectra of regular scaffolds like 1a. This additional band was therefore exploited to monitor the unfolding of bulged *ADAPTins*2 and the melting data were found to be in general agreement with the global hairpin unfolding results. In addition, NMR (nuclear magnetic resonance) chemical shifts were measured to determine variations in backbone tertiary structures (Fig. 2(B)). Secondary chemical shift deviations (CSDs) were calculated against random coil values^69^ for both *ADAPTin* scaffolds 1*vs.*2 to verify that the hairpins were folded and if any variation of conformational rigidity exist within the loops. As expected, successive backbone CSDs of H_α_ protons within the β-straps of 1a–b and 2a–b are relatively large (0.5–0.9 ppm) confirming a β-sheet register. In addition, the β-bulge residues D and Y (marked with asterisk in Fig. 2(B)) appeared abnormally deshielded in 2a–b (up to 0.7 ppm) suggesting that bulge-like *ADAPTins* present an extension of β-sheet structure. Notably, a number of secondary chemical shifts observed within the loops of the scaffold 2a and *ADAPTin*2b differ by more than 0.1 ppm than their regular hairpins counterparts 1a and 1b respectively suggesting that the bulged scaffold generate a more structured and strained loop.^70^

### Proof of concept: engineering a library of CDR-H3 mimics targeting the PD1:PDL1 interface

To rationally design our library of anti-PD1 *ADAPTins*, high-resolution crystal structures of PD(L)1·AbDrug complexes were analyzed. Using a series of bioinformatic tools in the Rosetta Suite, RosettaDock-4.0^71^ and Peptiderive,^72^ the energy profile for each binding paratope was exploited to score the “hot loops” contacts (ESI,† Fig. S8–S13). These H3 loops were ranked based off their calculated binding free energy, the buried surface area of interaction (>20% total BSA), and the total number of contacts to PD1. The results summarized in Table 1 suggest that most CDR-H3s were found promising scaffolds in comparison to all the other CDRs (ESI,† Table S1). Ranking of H3 loops from overall scores of binding free energy, binding-surface areas, and RMSD was obtained as follows: pembrolizumab > tislelizumab ∼ GY-14 > mAb059c > MW11-h317. Overall, the pembrolizumab H3 loop was found to be unique presenting the largest surface of interaction of 460 Å^2^ (36% of the overall Ab·PD1 buried-surface area) encompassing 21 contact interactions across distal regions of the PD1 epitope (localized on C′D and FG loops of PD1).

**Table 1:** Library of *ADAPTins* with diversely functionalized H3 loops generated from an individual CDR-H3 binding analysis. Evaluation of folding and stability properties of the designed *ADAPTin* peptides 1–2

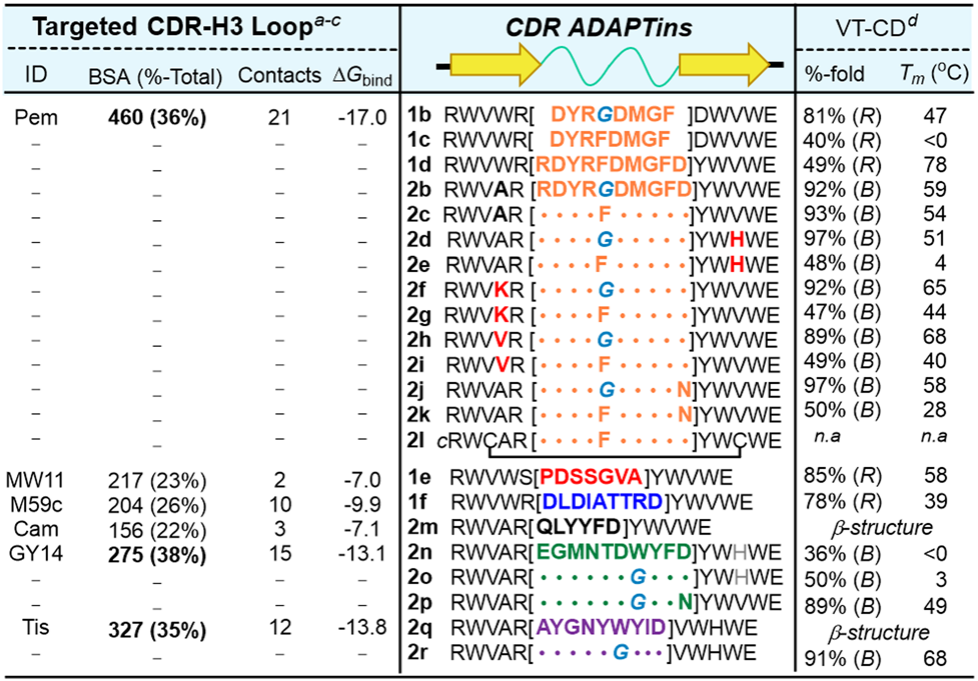

a Individual CDR buried surface area (BSA) computed by dr_SASA with %-binding surface calculated as a ratio to the total BSA (TBSA) from Ab·PD1 cocrystal structures.

b Total number of hydrophobic and polar binding contacts created at the CDR-H3 interface.

c Binding Gibbs free energy (reu) calculated by Peptiderive to score binding interfaces.

d Folded fraction (*χ*_F_) at 25 °C and melting temperatures calculated from CD-melts based on the type of *ADAPTin* fold (B: bulged, R: regular).

Having selected a set of potential CDR-H3 binders (Table 1), we carried out the synthesis of a library of regular (R) and bulged (B) *ADAPTin* peptides 1b–f and 2b–r respectively, using a typical Fmoc-chemistry on solid support. The folding of these peptides was first verified by their CD signature (Fig. 2(A)) and CD-melts measurements. At the exception of peptides 2m and 2q which are characterized by a β-sheet structure (band at 214 ± 2 nm, and a lack of exciton at 228 ± 2 nm) suggesting a misalignment within the hairpin β-strap, most peptides folded as expected according to their designed (R)- or (B)-scaffolds (Table 1, detailed CD analysis provide in the ESI†). Strikingly, the introduction of a glycine residue within the loop mimics of pembrolizumab (F10G, 1b, 2b, 2d, 2f, 2h, and 2j, *vs.*1c, 2c, 2e, 2g, 2i, and 2k,), GY-14 (W12G, 2n*vs.*2o), and tislelizumab (W11G, 2q*vs.*2r) afforded in each case additional flexibility that enhanced the global folding. Within the entire library, 10 bulge-like *ADAPTins* out of 14 analogs 2a–r had a melting temperature above 37 °C (Table 1), showing that the β-strap design is adaptable to a large variety of CDR-H3 loops. While these results are consistent with the notion that the *ADAPT* technology can create hairpin with long loops (6 to 10-residue tested), our ability to fully extend and extrapolate these folding properties is currently limited by the nature of the loop sequences. It will therefore be necessary to further optimize the β-strap stability-potentially by mimicking the rigidification and maturation mechanisms of CDR-H3s-to improve and generalize the *ADAPT* technology to a broader range of loop sequences and lengths.

**Fig. 3:**
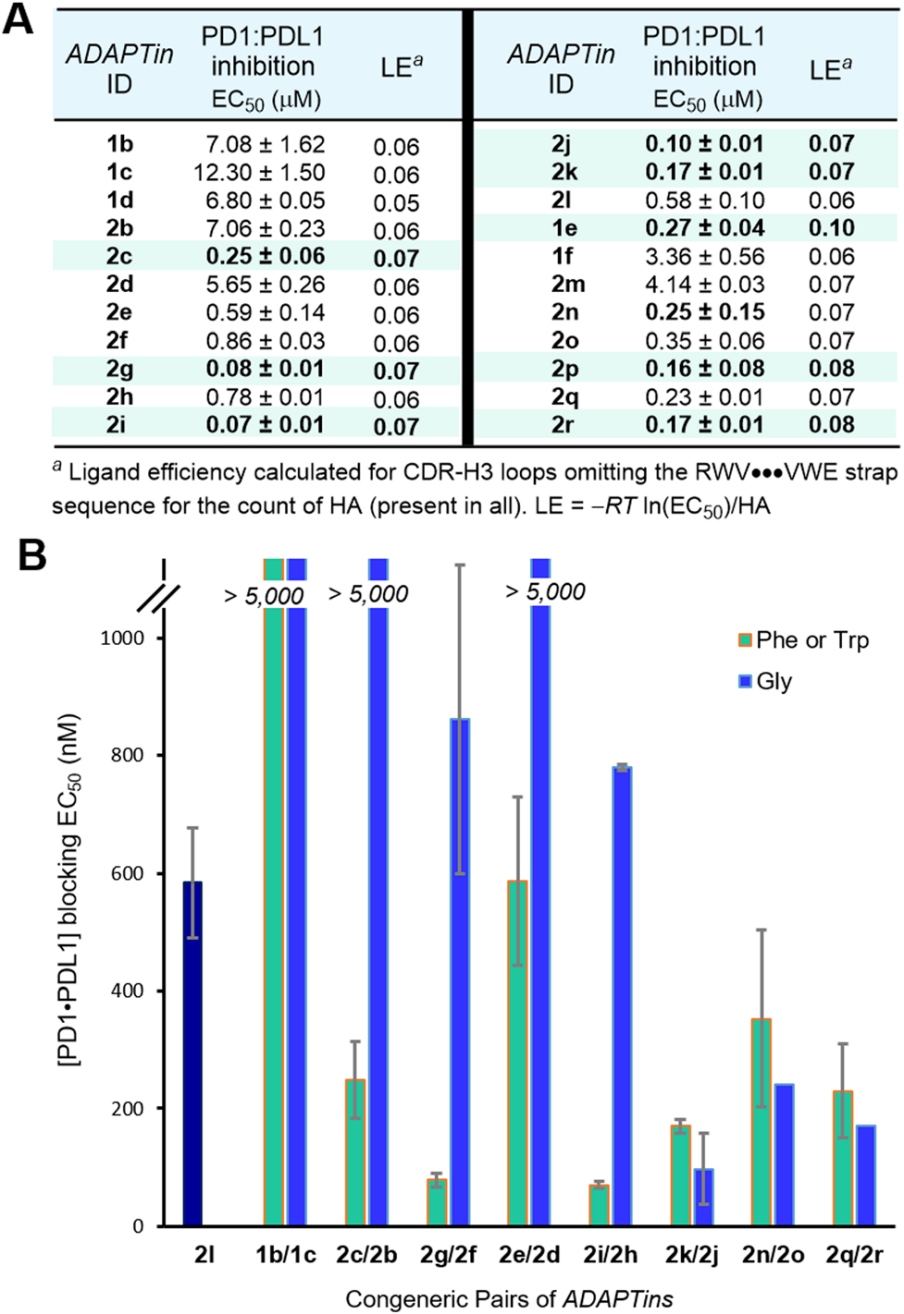
Inhibitory activity and ligand efficient of non-covalent *ADAPTins* against the PD1:PDL1 interaction. (A) Mean EC_50_ values determined from dose-dependent binding curves obtained by ELISA in a PD1–PDL1 assay; *n* = 3, SD reported in the ESI.† (B) Comparative plot of PD1·PDL1 inhibitory activity by single side chain modulation within *ADAPTins*. Original loops with Phe/Trp and their Gly-derived analogs. Experimental EC_50_ values from inhibitory dose–response curves. Error bars indicate the mean ± SD, (*n* = 3).

Given the significant structural differences between regular and bulged *ADAPTins*, we investigated if different loop topologies resulted in different PD1 binding affinity. Examination of interference of *ADAPTins*1 and 2 on the binding of PDL1 to PD1 was performed with a fluorescence-based ELISA (Fig. 3(A)). The blocking activity of these peptides was obtained and compared to a macrocyclic hairpin *cyclo*-2j (EC_50_ of 140 nM) and to the FDA-approved anti-PD1 antibody pembrolizumab (IC_50_ of 1 nM, positive control). Regarding regular *ADAPTins*, the MW11 mimic 1e offered the strongest activity (EC_50_ of 270 nM), while three different bulge-like scaffolds inspired by pembrolizumab (2c, 2g, 2i–k), but also by GY-14 (2n,p), and tislelizumab (2q–r) all presented promising blocking activities (EC_50_ < 300 nM). Given that all *ADAPTins* are approximately of same size, a ligand efficiency (LE) metric was calculated to better compare the H3 loops’ affinity to PD1, (Fig. 3(A)).^73^ By this measure, the LEs of *ADAPTins* studied herein (range 0.05–0.10, mean of 125 HA) were about two-fold lower than the comparably large peptide scaffold SFTI-1 (LE of 0.145 for 105 HA).^18^ This analysis suggested that *ADAPTins*1e, 2c, 2g, 2i–k, 2p, and 2r possess both potency and loop-display efficiency to block the PD1:PDL1 interaction. In addition, the plot of inhibitory activities of *ADAPTins* by congeneric pairs (Fig. 3(B)) revealed that the substitution of tryptophan or phenylalanine residues by glycines in the loops resulted in most cases in a substantial reduction of activity. These results mirror the membrane permeation measurements previously reported for those *ADAPTins*,^74^ highlighting the importance of hydrophobic residues to enhance both the passive diffusion and the pharmacological activity of long loops. Taken together, these results suggest that the *ADAPT* technology could become a new tool for mimicking antibody CDR-H3 structures; yet these miniaturized scaffolds have obviously lost a great deal of potency in comparison to their full-length parental antibodies (*K*_D_ low nano- to picomolar range).^75^ Indeed, the *k*_on/off_ kinetics of anti-PD1 mAbs are characteristic of very tight binders that attach almost irreversibly to PD1 (residence time in the order of v6 hours).^76^ To remediate to the lower potency of *ADAPTins*, we sought to further exploit these scaffolds to create irreversible covalent binders of PD1.

### Optimization of electrophilic *ADAPTin* inhibitors

**Fig. 4:**
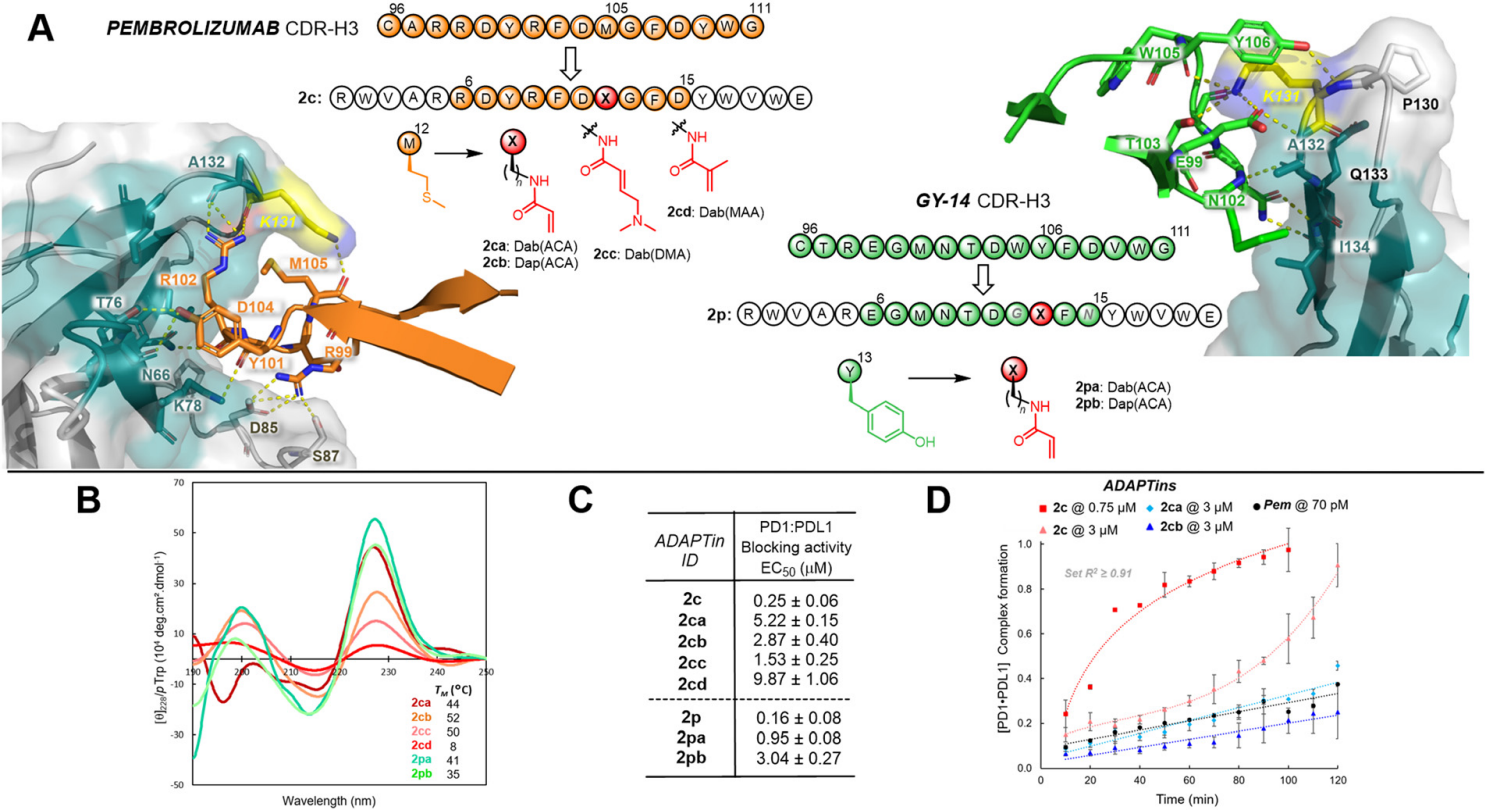
Design of lysine-targeted acrylamide-derived electrophilic inhibitors of PD1. (A) Pembrolizumab (orange) and GY-14 (green) bulged CDR-H3 hairpin loops in contact with PD1 (PDB codes: 5GGS and 6J14). Sequence of *ADAPTins*2c and 2p showing substitutions of M12 and Y13 respectively with Dap/Dab residues attached to acrylamide-derived electrophiles. M12 and Y13 were selected for the introduction of electrophilic warheads based on distances between the corresponding M105 and Y106 to the targeted _PD1_K131, see ESI,† Fig. S15. (B) CD spectra for the electrophilic *ADAPTins*2ca–cd/2pa,b and *T*_M_ values obtained from a global fit of CD-melts representing the temperature at which each peptide retain 50% folding. (C) Inhibitory activity of the covalent inhibitors against the PD1:PDL1 interaction, *n* = 3 (mean ± SD). (D) Kinetic curves of competitive inhibition targeting the PD 1: PDL1 interaction (1 : 12 ratio) showing the irreversible covalent nature of electrophilic inhibitors 2ca and 2cb. Error bars indicate the mean ± SD, *n* = 4.

While downsizing a full antibody drug into a single CDR-H3 “hot loop”, one should anticipate a significant loss in binding affinity and specificity. We previously reported that the non-covalent *ADAPTin*2c inhibited the PD1:PDL1 interaction with an apparent *K*_i_ of 41 nM and a residence time on target of v30 minutes.^77^ Yet even at a 3 μM concentration, this competitive inhibitor was fully displaced by PDL1 within two hours of incubation (Fig. 4(D)). Therefore, we decided to strategically modify the initial *ADAPTin* hits into covalent binders to engage the PD1 target irreversibly (Fig. 4). The PD1 protein contains one free cysteine (Cys93) and displays three surface-exposed lysines (K78, K131, and K135). Based on the available crystallographic data of PD1 bound to antibody drugs (see ESI,† Fig. S14 and S15), lysine K131 positioned on the highly flexible _PD1_FG loop appeared to be the most attractive residues to target.^78,79^ Indeed, the FG loop was shown by us and others to have an important innate conformational plasticity and no hindering *N*-glycosylation sites that creates a large surface of direct contacts with anti-PD1 mAbs.^77–81^ This flexibility enables a shallow binding-groove to form upon contact with the anti-PD1 pembrolizumab CDR-H3 loop (Fig. 4(A)).^82^ In addition, the FG loop conformation was suggested to influence the downstream signaling of PD1.^80,83^ For these reasons, *ADAPTins*2c and 2p, respective mimics of pembrolizumab and GY-14 CDR-H3s, were selected to introduce electrophilic warheads for covalent binding. For 2c, the methionine M105 in van der Waals interaction with _PD1_K131 (within v4.0 Å) was deemed appropriately positioned for modification, while for 2p tyrosine Y106 was the closest interacting residue to the _PD1_K131 (see ESI,† Fig. S15). To modify *ADAPTin*s 2c and 2p, we selected two anchoring amino acids of different side chain length 2,3-diaminopropionic acid (Dap, *n* = 1) and 2,4-diaminobutyric acid (Dab, *n* = 2) that can be readily installed by solid-phase peptide synthesis (SPPS). Due to their tunable electrophilicity,^84,85^ a series of acrylamide-type electrophilic warheads was generated on Dap/Dab residues at the selected positions of *ADAPTin* loops, including acrylamide (ACA), dimethylaminobutenamide (DMA), and methacrylic amide (MAA) (Fig. 4(A)). Acrylamides are physiologically stable, yet powerful and selective Michael acceptors that have demonstrated efficacy in a number of covalent drugs^86,87^ in particular targeting surface exposed lysines.^88,89^

First, the folding of these electrophilic peptides 2ca–d and 2pa–b was confirmed by CD spectroscopy (Fig. 4(B)). Excitingly, most analogs (at the exception of 2cd) were well folded (*χ*_F_ > 68%, *T*_M_ > 35 °C) presenting both bands at 202 and 228 ± 2 nm characteristic of a bulged-like β-hairpin scaffold and their melting curves were in each case very similar to the corresponding non-covalent *ADAPTin* molecules. To obtain a more accurate estimate of folding, we developed a global fit protocol that allowed the CD-melts of parent *ADAPTins*2c and 2p to be fitted simultaneously to their covalent congeners (see ESI,† Fig. S18 and S19). The resulting denaturation curves and melting temperatures strongly suggest that the introduction of acrylamide-derived warheads on either Dap or Dab amino acids did not substantially interfere with the intended hairpin fold. Next, the inhibitory activity of these electrophilic analogs was measured on the PD1:PDL1 interaction by ELISA (Fig. 4(C)). The six covalent analogs inhibited the interaction in a dose dependent manner with IC_50_s in the low micromolar range. By comparison, the inhibitory activity of all these covalent inhibitors is about 10-fold weaker than the parent *ADAPTins*2c and 2p, which could presumably be imparted by either the steric hindrance of the warhead, a change in the loop topology, or a deceleration of binding kinetics. Next, we asked if these covalent inhibitors could still exhibit high binding affinity in our competitive assay under saturating conditions of PDL1 (Fig. 4(D)). Time-course experiments were repeated by preincubating the non-covalent inhibitor 2c in one case, or the covalent *ADAPTins*2ca and 2cb at the same concentration. Under these physiologically relevant conditions (PDL1 excess: 12-fold), the binding profile of both electrophilic peptides 2ca and 2cb is consistent with an irreversible inhibition affording a complete blockade of the PD1:PDL1 interaction. Over the course of two hours, both *ADAPTins*2ca and 2cb bonded covalently to PD1 leading to a complete blockade similar to the one observed with the full-length pembrolizumab antibody (at 70 pM). These results are in stark contrast to the non-covalent inhibitor 2c which was easily displaced by the excess ligand PDL1.

### Covalent bonding to the PD1 protein

**Fig. 5:**
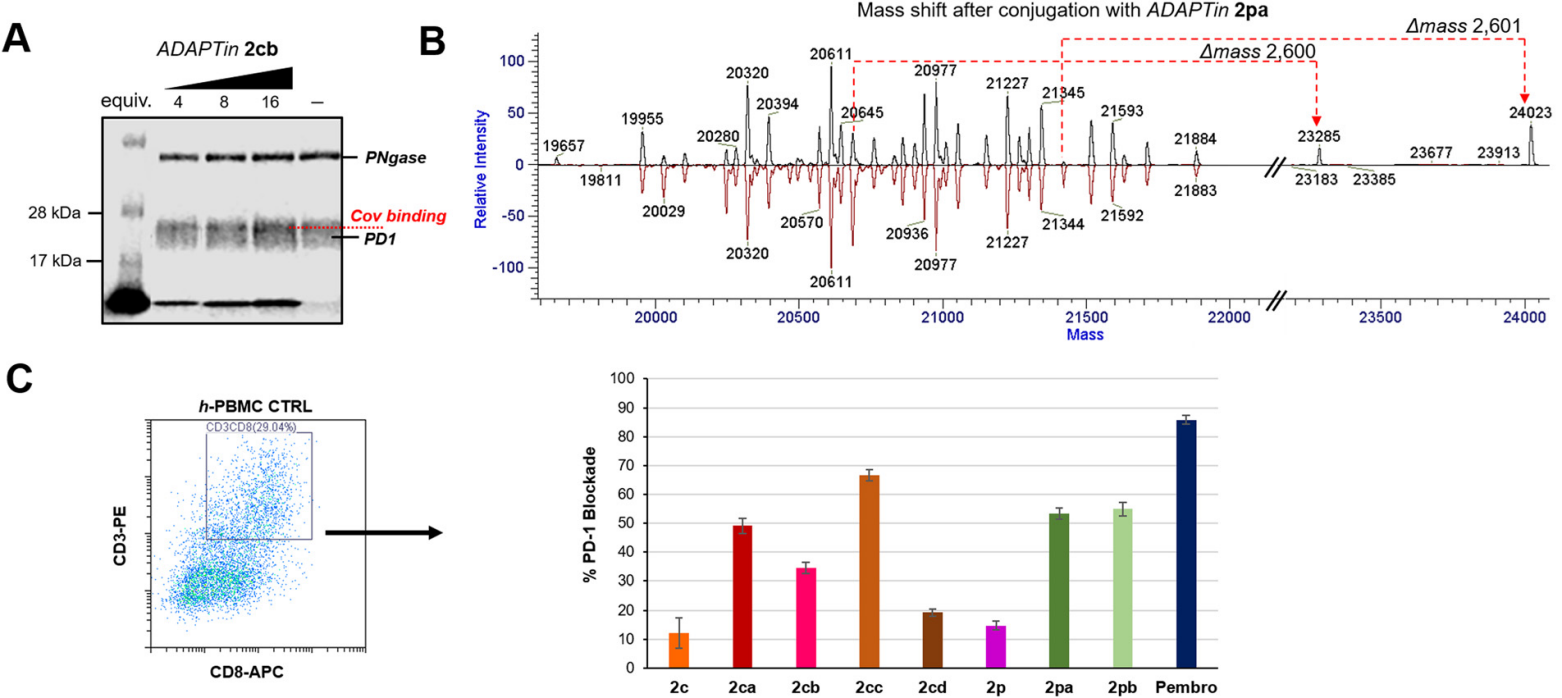
Electrophilic *ADAPTins* activity on the PD1 Protein. (A) Denaturing SDS-PAGE gel analysis showing a dose-dependence conjugation of 2cb to PD1 after incubation (12 h, 37 °C). No conjugation observed with 2c (negative control). (B) Mass spectrometry characterization of PD1 conjugation with 2pa (conjugation reaction with protein : peptide 55 : 220 μM, pH 8.0, 48 h). Deconvolution of the PD1 glycol-forms before (bottom red) and after (top black) conjugation with 2pa. (C) *In cellulo* PD1 blockade by covalent *ADAPTins*2ca–cd and 2pa–pb*versus* non-covalent analogs 2c and 2p and the full antibody control pembrolizumab. Cell surface level of PD1 blockade in PBMC cultures (30 h) determined by flow-cytometry analysis. Cells were gated on CD45^+^ and CD3^+^/CD8^+^ cells were selected from the CD45^+^ fraction; error bars indicate the mean PD1%-blockade ± SD (*n* = 3).

PD1 is heavily *N*- and *O*-glycosylated at positions N49/58/74/116 and T153/168, S157/159 respectively, therefore to assess the covalent binding of our inhibitors and generate a less diffused PD1 band on SDS-PAGE gel, the extracellular portion of the protein was first incubated with PNGase F. None of the glycosylation sites are near the binding contacts of pembrolizumab or GY-14 CDR-H3 loops, supporting the idea that such deglycosylated PD1 model protein remains relevant. Reaction of the electrophilic *ADAPTin*2cb with *N*-deglycosylated PD1 was investigated at 37 °C under different concentrations, and incubation times (Fig. 5(A)). It was found that the formation of covalent PD1-conjugates could be assessed in a dose-dependent manner at pH 6.5 using a minimum incubation of 12 h and 4 eq. of 2cb. Incubation with the non-covalent peptide 2c did not produce any higher negative molecular weight adduct on the SDS-PAGE 10% bis-tris gels (negative control), while the incubation of 2cb produced a slightly higher molecular conjugates (darker band) at peptide/protein ratios ranging from of 4 : 1 to 16 : 1 (Fig. S21 and S22, ESI†). Yet, due to the large number of *O*-glycans still present on PD1, smearing of the PD1 band (across ∼5 kDa) rendered the visualization and isolation of conjugated adducts difficult (Fig. S22, ESI†). Trypsin-digested bands of plausible conjugated adducts were subjected to MS/MS analysis, but none of the fragmentation patterns expected for the conjugation of *ADAPTins* to PD1 were observed. Therefore, we turned our attention to the direct incubation of PD1 to our electrophilic peptides (2 days at pH 8 and physiological temperature) and the detection by intact mass spectrometry (Fig. 5(B)). The deconvoluted mass spectrum of intact *N*-deglycosylated PD1 presented a number of masses ranging from 19 995 to 21 884 Da (Fig. 5(B), bottom red spectrum). The results of *ADAPTin*2pa incubation suggested that the peptide bonded covalently to certain glycol-forms of PD1 in a 1 : 1 complex affording new peaks with a Δ*m* of 2600 Da. In contrast, peptide 2pb was less selective and found to form 1 : 1 and 1 : 2 complexes with PD1 (Fig. S24 and S25).

### Cell-based antitumor immunity rescue

**Fig. 6:**
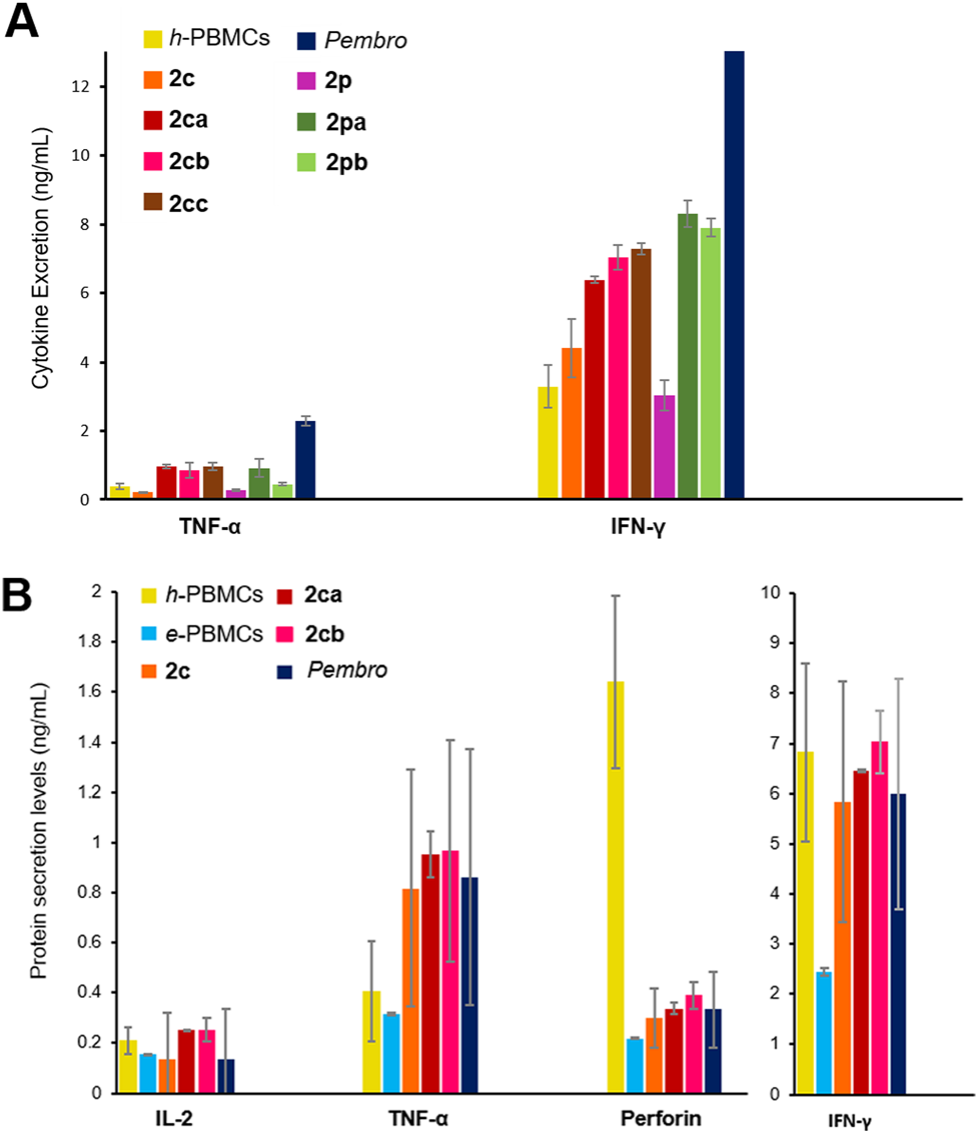
*Ex vivo ADAPTin* activity on the PD1 checkpoint pathway. Non-covalent *ADAPTin*2c/2p were used as negative control. (A) Levels of inflammatory cytokines measured by flow-cytometry (FACS) after 6 h incubation with electrophilic *ADAPTins*. Error bars indicate the mean ± SD, (*n* = 3). (B) Rescue of exhausted PBMCs from a melanoma patient. Levels of inflammatory cytokines and granule-associated proteins released (6 h) measured by FACS (healthy and untreated exhausted PBMCs: h-/e-PBMCs used as positive and negative controls respectively). Error bars indicate the avg ± SD, (*n* = 2).

To verify the binding efficacy of *ADAPTins* under more physiologically relevant conditions *in cellulo*, the binding of PD1 to non-covalent inhibitors 2c/2p and the corresponding electrophilic analogs 2ca–cd and 2pa–pb was examined on healthy human peripheral blood mononuclear cells (h-PBMCs) by flow cytometry (Fig. 5(C)).^90^ Levels of cell surface PD1 on h-PBMCs was measured after 30 hours of incubation with or without anti-PD1 peptides and compared to the levels measured with pembrolizumab (positive control, >95% blockade at 100 nM, see ESI,† Fig. S26). Compared to the non-covalent molecules 2c/2p (<20% binding), the efficacy of our electrophilic analogs 2ca, 2cc, and 2pa–pb on T-effector cells (CD3^+^/CD8^+^) were significantly higher, achieving 48 to 67 ±10% of PD1 blockade (Fig. 5(C)). These results confirmed that several electrophilic peptides not only irreversibly bind to free PD1 but also to CD8^+^ T cells expressing high levels of PD1 on their membrane. In addition, this cell-based assay suggests that *ADAPTins* achieved their covalent binding task while resisting (at least partially) to proteolytic degradation over the two-day experiments. Then, an *ex vivo* immune cell culture system using h-PBMCs and exhausted PBMCs from a melanoma patient (e-PBMCs) was used to determine the efficacy of selected *ADAPTin* peptides on T-effector cells (Fig. 6).^91,92^ T-cell exhaustion typically leads to a reduction in cytokine release, cytotoxic activity, as well as slower T-cell proliferation. Cytokine secretions were assayed after the reactivation of h-PBMCs and e-PBMCs with anti-CD2/28 antibodies and a six-hour incubation period with *ADAPTins* or pembrolizumab (positive control). As illustrated in Fig. 6(A), while non-covalent inhibitors 2c/2p had no detectable activity on cytokine secretions, an incubation with electrophilic *ADAPTin* analogs 2ca, 2cc, and 2pa–pb resulted in significant increases (2 to 5-fold) in TNF-α, IFN-γ, and perforin levels. In regards to the treatment of exhausted PBMCs (Fig. 6(B)), levels of inflammatory cytokines exhibited a substantial increase (TNF-α, IFN-γ, up by 3-fold to full recovery, IL-2 up by 1.5-fold) compared to untreated e-PBMCs and h-PBMCs (considered as two negative controls). Interestingly, the secretion of those cytokines are a hallmark of cytotoxic T lymphocytes (CTLs) response mediated by type-I natural killer (NK) cells.^93^ IL-2 concentrations reaching levels comparable to that observed upon treatment with pembrolizumab indicated that our anti-PD1 *ADAPTins* could promote the reactivation of an immune response and the rescue of effector T-cells proliferation. Along these lines, the significant increase in secreted cytoplasmic granule-associated proteins (perforin and granzyme B, levels >2-fold), particularly upon incubation with 2cb, suggested an activation of T-cell cytotoxicity, potentially related to NK cells activity.^94^ Cytokines concentrations measured at 72 h post-treatment were in most cases over the detection limit range further indicating the effectiveness of our compounds over time. Taken together, results from this *ex vivo* PBMC model suggest that *ADAPTin*2cb achieved a long-lasting PD1 inhibition and a potent rescue of T-cell proliferation and effector function.

## C. Conclusions

This study provides a proof-of-concept for transposing CDR-H3 structures found in antibodies into independent stand-alone *ADAPTin* peptide scaffolds. Our combined NMR and thermal CD-denaturation data demonstrated that diverse CDR-H3 loop sequences can be mounted into stable *ADAPTins*. Out of six anti-PD1 antibodies (pembrolizumab, MW11, M59c, camrelizumab, GY14, and tislelizumab), four native CDR-H3 loops were successfully transposed into folded scaffolds (1e, 1f, 2c, and 2n). Of the 29 peptides synthesized, over 70% demonstrated thermal stability above 37 °C. Although further NMR studies will be required to fully understand the structuration of β-bulges in *ADAPTins*,^32^ these motifs found at the apex of antibody CDR-H3s appear to play a pivotal role in structuring a large variety of loops. Notably, eight non-covalent *ADAPTins* effectively blocked the PD1/PDL1 protein–protein interaction at low nanomolar inhibitory concentrations (EC_50_ below 0.5 μM). Introducing acrylamide-based warheads to *ADAPTins*2c and 2p led to covalent binding despite a loss of affinity (∼10-fold). Even though electrophoresis gels and intact mass experiments further validated the covalent binding of 2cb and 2pa, peptide epitope mapping by MS/MS analyses were complicated by the high level of PD1 glycosylation. To establish the specificity of covalent bonding to the targeted _PD1_K131 residue and ultimately validate the antibody CDR-H3 biomimicry, further *O*-deglycosylation strategies will be evaluated^95^ as well as the introduction of more reactive lysine-targeting warheads.^96,97^ While further studies are needed to confirm specific PD1 residue targeting, covalent *ADAPTins* already showed superior PD1 binding *in vitro* and the rescue of exhausted PBMCs through cytokines secretion. The success of this scaffold highlights *ADAPTin*'s potential for peptide display and biological screening, offering a novel biomimetic platform to target protein–protein interactions implicated in human diseases. With growing antibody structural databases, CDR-H3 mimics can be rationally designed and further optimized using high-throughput mRNA- or phage-display technologies^53^ to position these scaffolds in the therapeutic space of PPI inhibition.

## Author contributions

This project was conceived by S. P. R.; S. H. N., A. D. R., G. Z., and L. B. per-formed the research experiments and S. P. R. with S. H. N. analysed the data. Biological studies were coordinated by C. J. W, M. W., and N. S. and the mass spectrometry experiments were directed by C. P. D. The manuscript was written through contributions of S. P. R. and S. H. N.; all authors have given approval to the final version of the manuscript.

## Conflicts of interest

There are no conflicts to declare.

## Supplementary Material

CB-005-D4CB00174E-s001

CB-005-D4CB00174E-s002

## Data Availability

The data supporting this article have been included as part of the ESI.† The structural analysis of CDR-H3 binding surfaces was carried out using publicly available data of six different full-length antibodies from the worldwide protein data bank at [URL – https://www.rcsb.org/] with [PDB Entries: 5B8C, 5GGS, 6J14, 6JJP, 6K0Y, 7BXA, and 7CU5]. The NMR-guided 3D-structure and conformational ensemble of our model CDR-H3 *ADAPTin*2b is also available at the worldwide protein data bank [PDB Entry – 8W0Q].
