# Supplementary material for: A platform of ADAPTive scaffolds: development of CDR-H3 β-hairpin mimics into covalent inhibitors of the PD1/PDL1 immune checkpoint
Source: RSC Chem Biol. 2024 Nov 4;5(12):1259–70. doi: 10.1039/d4cb00174e (PMC11562385; doi:10.1039/d4cb00174e)
Supplement: CB-005-D4CB00174E-s002 [file CB-005-D4CB00174E-s002.pdf]

Supplementary Information (SI) for RSC Chemical Biology.
This journal is © The Royal Society of Chemistry 2024

Supporting Information

A Platform of ADAPTive Scaffolds: Development of CDR-H3 β-Hairpin Mimics into Covalent Inhibitors of the PD1/PDL1 Immune Checkpoint

Sarah H. Naylon[a], Alexis D. Richaud[a], Guangkuan Zhao[a], Linda Bui[a], Craig, P. Dufresne[b], Chunjing J. Wu[c], Medhi Wangpaichitr[c], Niramol Savaraj[c], and Stéphane P. Roche[a]*

[a] Department of Chemistry and Biochemistry, Florida Atlantic University, Boca Raton, Florida 33431, United States.

[b] Thermo Fisher Scientific, West Palm Beach, Florida 33407, United States.

[c] University of Miami School of Medicine, 1475 NW 12 Ave, Miami, Florida 33136, United States.

Contents

1. **General Information** 4

2. **Structural Analysis of anti-PD1 Antibody Blockers from X-Ray Co-Crystal Structures** 5

   Figure S1. Mapping and Identification of pembrolizumab CDR-H3 loop interactions with PD-1 (PDB code: 5GGS). 6

   Figure S2. Mapping and Identification of pembrolizumab CDR-H3 loop interactions with PD-1 (PDB code: 5B8C). 7

   Figure S3. Mapping and Identification of mAb059C CDR-H3 loop interactions with PD-1 (PDB code: 6K0Y). 8

   Figure S4. Mapping and Identification of MW11-h317 CDR-H3 loop interactions with PD-1 (PDB code: 6JJP) 9

   Figure S5. Mapping and Identification of camrelizumab CDR-H3 loop interactions with PD-1 (PDB code: 7CU5) 10

   Figure S6. Mapping and Identification of GY-14 CDR-H3 loop interactions with PD-1 (PDB code: 6J14) 11

   Figure S7. Mapping and Identification of tislelizumab CDR-H3 loop interactions with PD-1 (PDB code: 7BXA) 12

3. **Structural Analysis Summary of anti-PD1 Blockers** 13

   Table S1. Selection of Potential PD-1 Inhibitors from known X-ray Crystal Structures of Antibody Drugs Complexes. 13

   Table S2. Calculated binding energies with macrocycle score from Peptiderive Server. 13

   Figure S8. Peptiderive generated figures for Tislelizumab. 14

   Figure S9. Peptiderive generated figures for Camrelizumab. 14

   Figure S10. Peptiderive generated figures for GY-14. 15

   Figure S11. Peptiderive generated figures for mAb059c. 16

   Figure S12. Peptiderive generated figures for MH11-h317. 17

   Figure S13. Peptiderive generated figures for Pembrolizumab. 18

4. **Lysine pKa Predictions** 19

   Table S3. Predicted lysine pKa values from crystal structure data imported to ROSIE server. 19

   Figure S14. Motion of lysine residues (K78, K131 and K135) on the extracellular portion of PD1 based on antibody interactions. 19

5. **Lysine 131 Contact Interactions with GY-14 and Pembrolizumab CDR-H3 loops** 19

   Figure S15. Identifying distances (within 4.0 Å) between (A) pembrolizumab M105 and $_{PD1}$K131 (PDB code: 5GGS) and GY14 Y106 and $_{PD1}$K131 (PDB code: 6J14). 20

6. **Peptide Synthesis, Purification and Structural Interpretation** 20

   Figure S16. Procedure for peptide synthesis and on-resin warhead incorporation *via* Fmoc-SPPS. 22

   Table S4. Summary of all peptides prepared in this study. 24

   Figure S17. Peptides' HPLC chromatograms. 25

7. **Comparison of *β*-hairpins Stability by Circular Dichroism (CD) Spectroscopy** 36

   Table S5. Summary of peptides thermodynamic and hairpin folding parameters calculated through Origin 9.0. 37

   Table S6. Comparison of folding and melting temperatures using individual and global fitting protocols for GY-14 peptides (covalent and non-covalent *ADAPTins*). 38

   Table S7. Comparison of folding and melting temperatures using individual and global fitting protocols for pembrolizumab peptides (covalent and non-covalent *ADAPTins*). 38

Figure S18. CD Analysis of GY-14 peptides. (A) Spectra taken every 10 degrees. (B-C) Melting curves obtained from a global fit protocol for the bands at 228 and 202 ± 2 nm.. 39

Figure S19. CD Analysis of pembrolizumab peptides. (A) Spectra taken every 10 degrees. (B-C) Melting curves obtained from a global fit protocol for the bands at 228 and 202 ± 2 nm.. 40

8. **Immunoblocking ELISA Assay** 41

Table S8. Tabulated mean EC_{50} values their corresponding standard deviations (*n* = 3) and the calculated ligand efficiency values (LE). 41

9. **Competitive Binding Kinetics** 42

Figure S20. A) Kinetic PDL1 binding curves at varying concentrations of 2k to determine K_i value. B) Kinetic PDL1 binding curves at varying concentrations of **2c** (non-covalent) to determine K_i value and with **2ca-cb** (covalent). 42

10. **SDS-PAGE and Mass Spectrometry Experiments for the Covalent Binding of ADAPTins to PD1** 43

Figure S21. Full SDS-Page with varying equivalence of peptide (2cb) to PD-1 43

Figure S22. Full SDS-Page with varying equivalence of peptides (2ca, 2cb, 2cc, and 2cd) to PD-1 (2 μg/well). 44

Figure S24. A) Deconvoluted mirrored spectra output for a direct comparison between deconvoluted PD1 masses recorded after incubation with **2pa** (top) versus pure PD1 control (bottom). 46

Figure S25. A) Deconvoluted mirrored spectra comparison output for a direct comparison between deconvoluted spectra of **2pb** (top) with respect to PD-1 control (bottom). 46

11. ***In Vitro PBMC Binding Data*** 47

Figure S26. Selected example of *In Vitro* PD1 blocking activity of *ADAPTins* 2 on healthy PBMCs measured by flow cytometry. 47

Table S9. Raw data of cytokine secretion assay 48

12. ***ADAPTin NMR Analysis and Selected Spectra*** 49

Table S10. NMR Chemical Shift Deviations (CSD, ppm) calculated using random coil values reported by Poulsen 49

Figure S27. Histograms of CSD for hairpins **1a,b** (Regular)and 2a,b (bulged) in water/DMSO-d_6 (90:10, v/v) 50

Figure S28. Schematic representation of the most relevant inter-residue NOESY cross-peak correlations for **1a**. 51

Figure S29. Schematic representation of the most relevant inter-residue NOESY cross-peak correlations for **2a**. 52

Table S11. Long range NOESY cross-peaks for peptide RW-VAR-GGGGKKGGFD-YWV-WE (**2a**) 53

1. General Information

X-ray structures analyzed in this study are publicly available at https://www.rcsb.org/. LigPlot+ software facilitated the interpretation of interactions, while PyMOL software was utilized for molecular visualization. Monomers demonstrating the greatest number of interactions were exclusively chosen, and additional interactions identified in other monomers were incorporated into the corresponding tables. Reagents such as Fmoc-amino acids and resins utilized in this study were procured from Chemimpex and Millipore Sigma. Bulk solvents were sourced from Fisher Scientific™. The assay kit used for evaluating inhibitors of the PD-1/PD-L1 interaction was obtained from BPS Bioscience. Peptides **2g**, **2h**, **2k**, **2i**, **2m**, and **2p** were purchased from Peptide2.0 with purity >95%. For all other peptides the synthesis was performed employing the standard automated Fmoc-SPPS method on a Protein Technologies PS-3 peptide synthesizer and using a Fmoc-Glu-Wang resin (0.6 meq/g). Peptide purification was done using a Hitachi L-7000 series HPLC equipped with a semi-preparative XBridge BEH C18 column (130 Å, 10μm, 10 mm x 250 mm). Analytical reverse-phase high-performance liquid chromatography (RP-HPLC) on a Hitachi L-7000 series equipped with an XBridge BEH C18 column (130 Å, 10 μm, 4.6 mm x 250 mm) determined peptide purity, while mass spectrometry using a Bruker Microflex LRF matrix-assisted laser desorption ionization time-of-flight (MALDI-TOF) determined their molecular weight. Intact mass spectra of the PD1 protein and the conjugates with *ADAPTins* were collected with an Orbitrap Fusion™ Lumos™ Tribrid™ Mass Spectrometer coupled to a Vanquish™ Neo UHPLC System (Thermo Scientific™). Peptide solutions for circular dichroism (CD) were prepared within a concentration range of 15-120 μM in a phosphate buffer (15 mM, pH 6.5) with the addition of MeOH (up to 20% v/v) as necessary for enhanced solubility. Solution UV-absorbance measured accurately using a JASCO V-670 spectrophotometer relied on the combined molar absorptivity of Trp (ε_{280} = 5580 M^{-1} .cm^{-1} per Trp) and Tyr (ε_{280} = 1280 M^{-1} .cm^{-1} per Tyr) to determine each sample's concentration. CD spectra were recorded using a JASCO J-810 Spectropolarimeter equipped with a temperature controller module JASCO PFD-425S. Raw CD melting curves were processed in SpectraGryph, and fitting was performed in OriginPro 9.0 (Originlab Corporation, U.S.A.). NMR samples were prepared by reconstituting freeze-dried peptides (~ 1-5 mg) in mixture of water and DMSO-d6 (90:10 v/v) using 2,2-dimethyl-2-silapentane-5-sulfonate (DSS) as internal standard for chemical shifts (0 ppm), and spectra were recorded at 291 K (18 ^{o}C) unless otherwise stated. Chemical shifts (δ) are reported in ppm. Samples for NMR experiments were prepared in a range of 3-10 mM of peptide in solution unless otherwise noted. NMR spectra were recorded on a Varian Mercury500 (500 MHz) spectrometer using the Vnmrj 4.2 software; ^{13}C NMR and ^{1}H^{15}N HSQC were recorded on a Bruker 400 Ultrashield (400 MHz) spectrometer using the Topspin 3.5 software. Residual water peak was suppressed using a PRESAT pulse sequence. Full NMR spectral data, tabulated chemical shift deviations data, and NOESY schematics for *ADAPTin* scaffolds **1a**/**2a** and the corresponding inhibitors **1b**/**2b** are presented in section 12. For the PD1 immunoblocking assay, inhibitor solutions ranging from 0.01 to 100 μM along with a blank (DMSO/PBS buffer solution) were prepared. SDS-Pages, buffers, and stain were purchased from Fisher Scientific. Commercial PD1 for SDS-Page and mass spectrometry was purchased from Fisher Scientific (R&D Systems™).

2. Structural Analysis of anti-PD1 Antibody Blockers from X-Ray Co-Crystal Structures

Briefly, publicly available co-crystal structures of anti-PD1 antibody blockers were obtained from the Protein Data Bank (March 2021). The interpretation of contact interactions was assisted by LigPlot+ [SI-1], and the molecular visualization with PyMOL.[SI-2] In each crystal, the monomer displaying the largest number of contact interactions was analyzed, and supplemental interactions found inside other monomers were included if the residues were not found to be conformational outliers. A maximum cut-off of 2.70 Å for hydrogen bonds (Hydrogen-Acceptor distance), 4.0 Å for salt bridges (Donor-Acceptor distance), and a range of 2.90 -3.90 Å for hydrophobic contacts distances were implemented for the binding analysis in LigPlot+. X-ray structure validation reports were used for each complex (PDB codes: 5GGS/5B8C, 6K0Y, 6JJP, 7CU5, 6J14, and 7BXA) to identify steric outliers' interactions. Residues with a mean B-factor higher than the B-factor high-median value of the entire protein were removed from consideration. For each crystal structure, the summary of polar contacts and hydrophobic interactions created by the CDR-H3 loop is detailed in **Figures S1-S7**. The total binding surface area (TBSA) of PD1 complexed with the whole antibody was calculated, as well as the binding surface of each individual CDR to determine the CDR-H3 surface to TBSA percentage (**Table S1**). Heat maps of CDR-H3 binding generated in dr_SASA[SI-3] also helped to estimate the contact surface area of each individual CDR-H3 loop residue to the PD1, guiding the selection of site-specific modifications on *ADAPTins* and of targetable lysines for covalent binding on PD1.

Figure S1. Mapping and Identification of **pembrolizumab** CDR-H3 loop interactions with PD-1 (PDB code: 5GGS). (**A**) Box diagram overview of contact interactions (red: salt bridges; blue: hydrogen bonds; green: water-mediated hydrogen bonds; yellow hydrophobic interactions). (**B**) Heat map of CDR-H3 residues involved at the contact surface area with PD-1. (**C**) Close-up view of CDR-H3 loop residues forming contact interactions with PD-1 (residues involved in hydrophobic interactions outlined in lighter color)

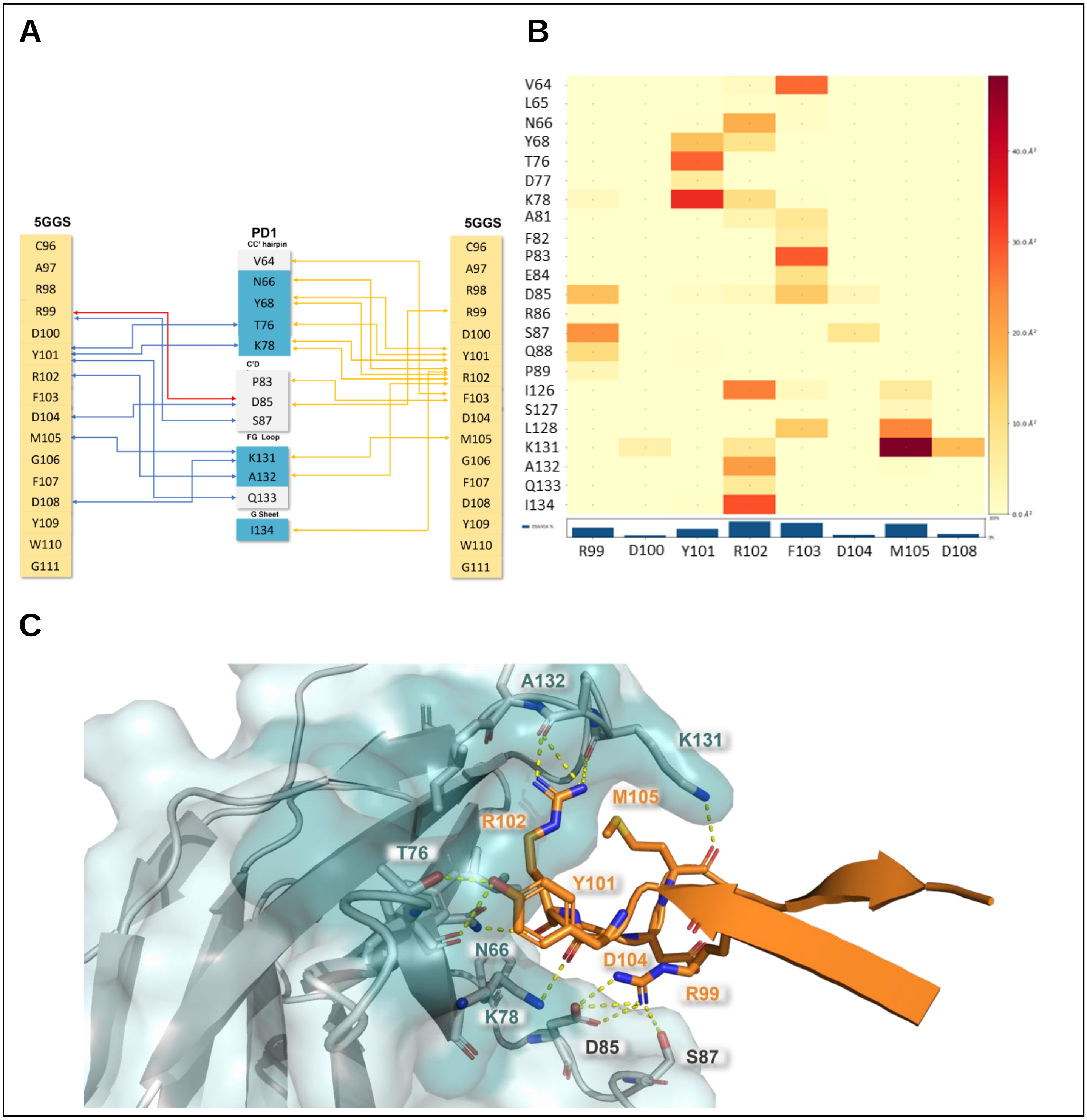

Figure S2. Mapping and Identification of **pembrolizumab** CDR-H3 loop interactions with PD-1 (PDB code: 5B8C). (**A**) Box diagram overview of contact interactions (red: salt bridges; blue: hydrogen bonds; green: water-mediated hydrogen bonds; yellow hydrophobic interactions). (**B**) Heat map of CDR-H3 residues involved at the contact surface area with PD-1. (**C**) Close-up view of CDR-H3 loop residues forming contact interactions with PD-1 (residues involved in hydrophobic interactions outlined in lighter color)

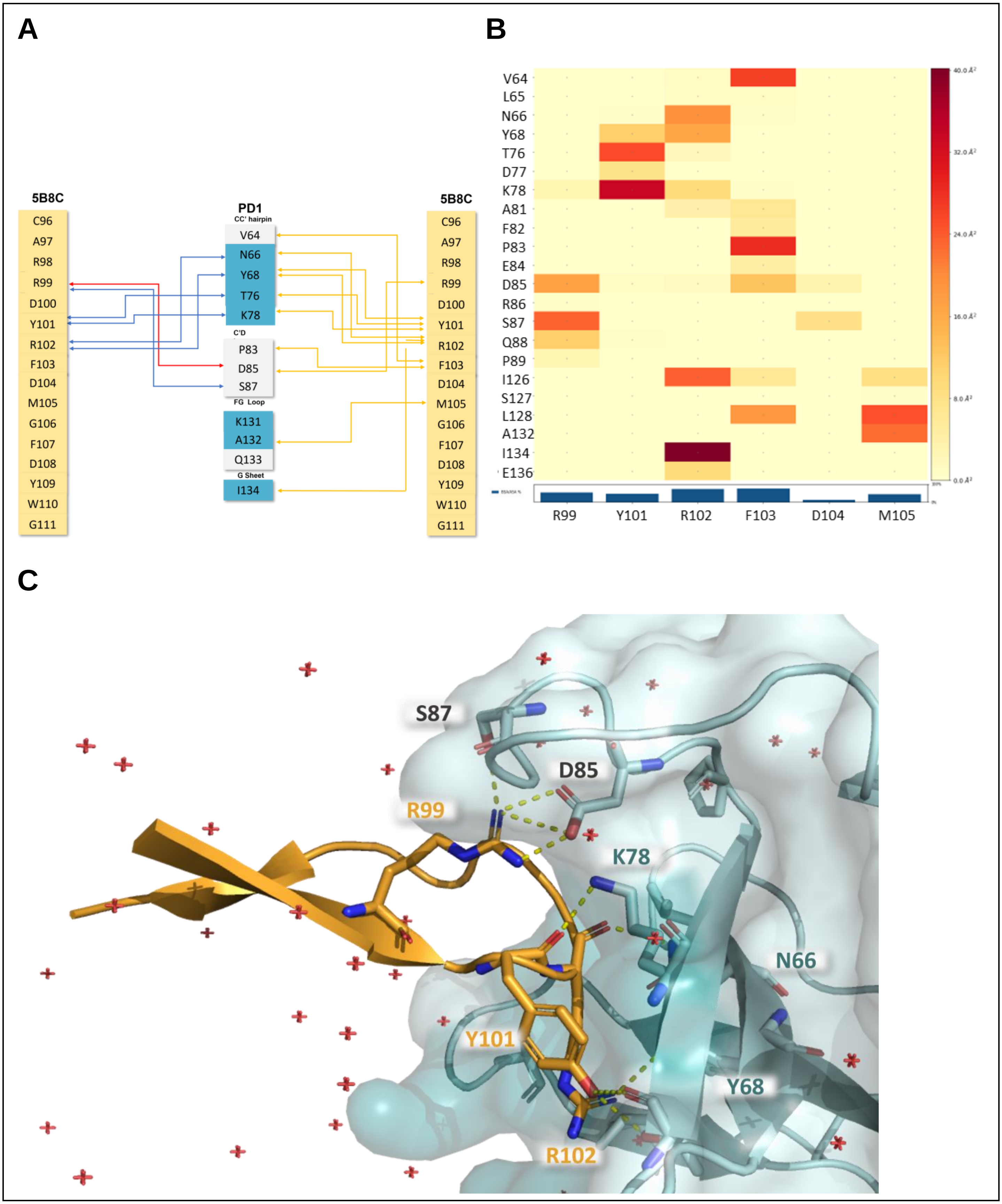

Figure S3. Mapping and Identification of **mAb059C** CDR-H3 loop interactions with PD-1 (PDB code: 6K0Y). (**A**) Box diagram overview of contact interactions (red: salt bridges; blue: hydrogen bonds; green: water-mediated hydrogen bonds; yellow hydrophobic interactions). (**B**) Heat map of CDR-H3 residues involved at the contact surface area with PD-1. (**C**) Close-up view of CDR-H3 loop residues forming contact interactions with PD-1 (residues involved in hydrophobic interactions outlined in lighter color)

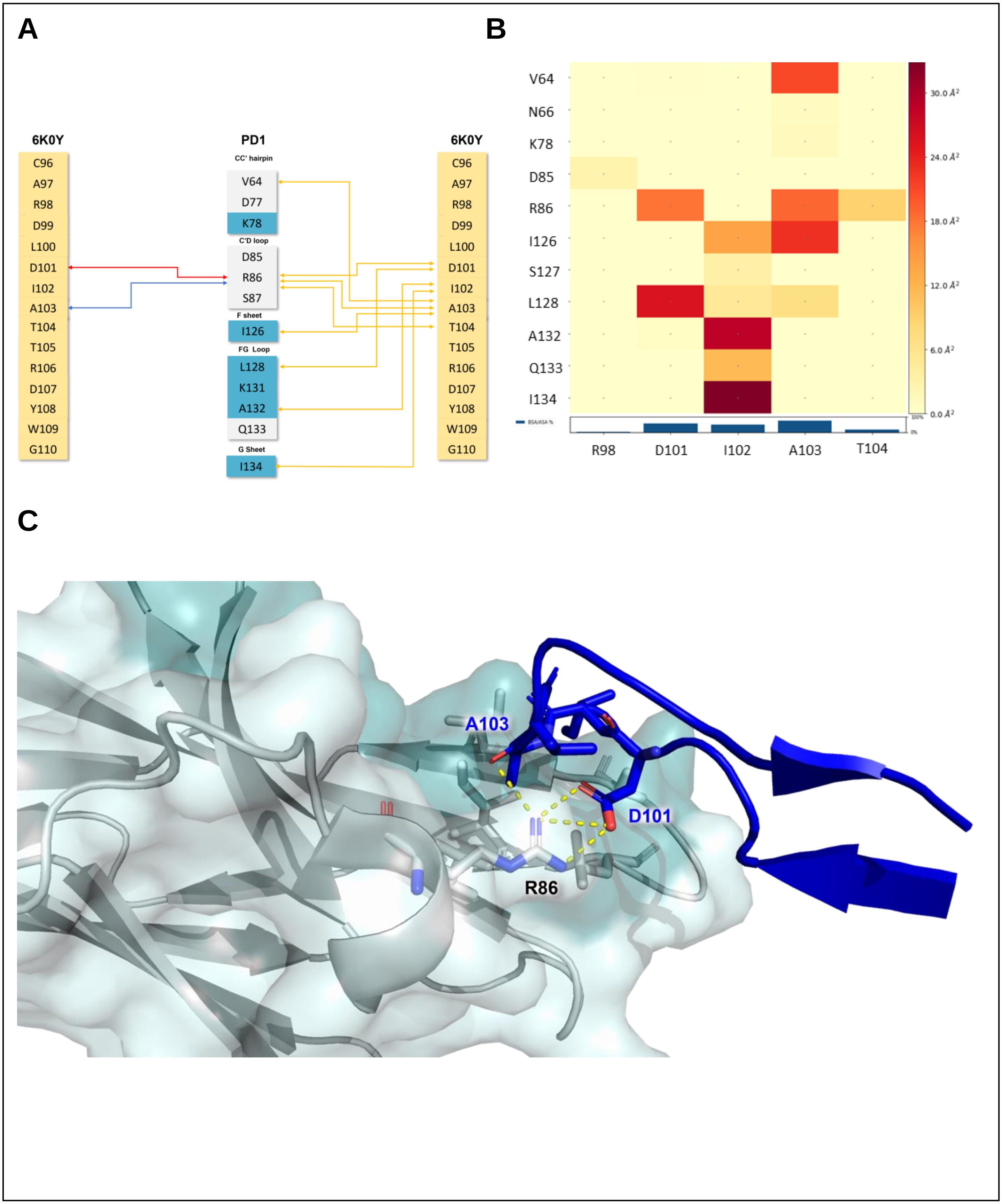

Figure S4. Mapping and Identification of **MW11-h317** CDR-H3 loop interactions with PD-1 (PDB code: 6JJP). (**A**) Box diagram overview of contact interactions (red: salt bridges; blue: hydrogen bonds; green: water-mediated hydrogen bonds; yellow hydrophobic interactions). (**B**) Heat map of CDR-H3 residues involved at the contact surface area with PD-1. (**C**) Close-up view of CDR-H3 loop residues forming contact interactions with PD-1 (residues involved in hydrophobic interactions outlined in lighter color)

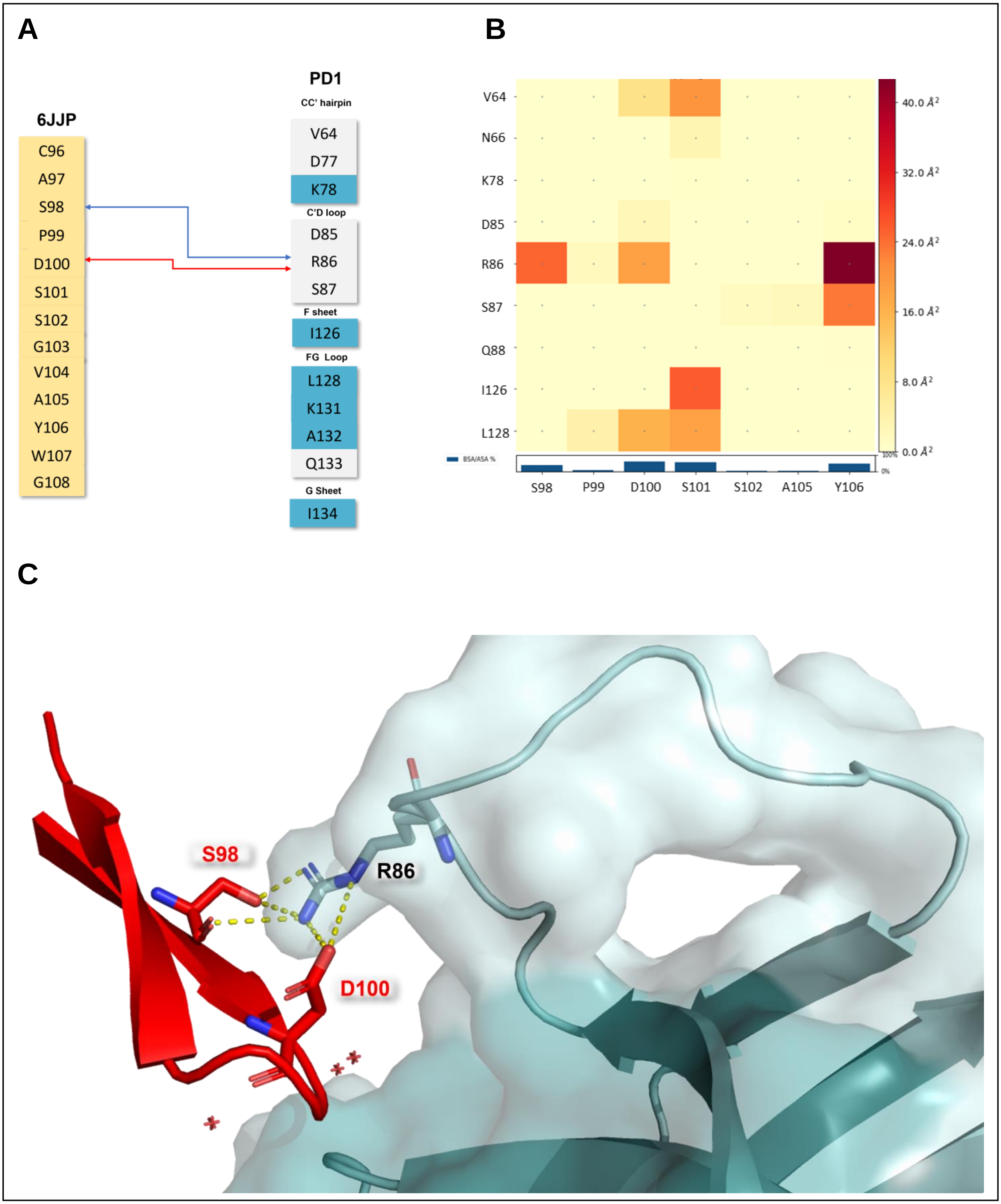

Figure S5. Mapping and Identification of **camrelizumab** CDR-H3 loop interactions with PD-1 (PDB code: 7CU5). (**A**) Box diagram overview of contact interactions (red: salt bridges; blue: hydrogen bonds; green: water-mediated hydrogen bonds; yellow hydrophobic interactions). (**B**) Heat map of CDR-H3 residues involved at the contact surface area with PD-1. (**C**) Close-up view of CDR-H3 loop residues forming contact interactions with PD-1 (residues involved in hydrophobic interactions outlined in lighter color)

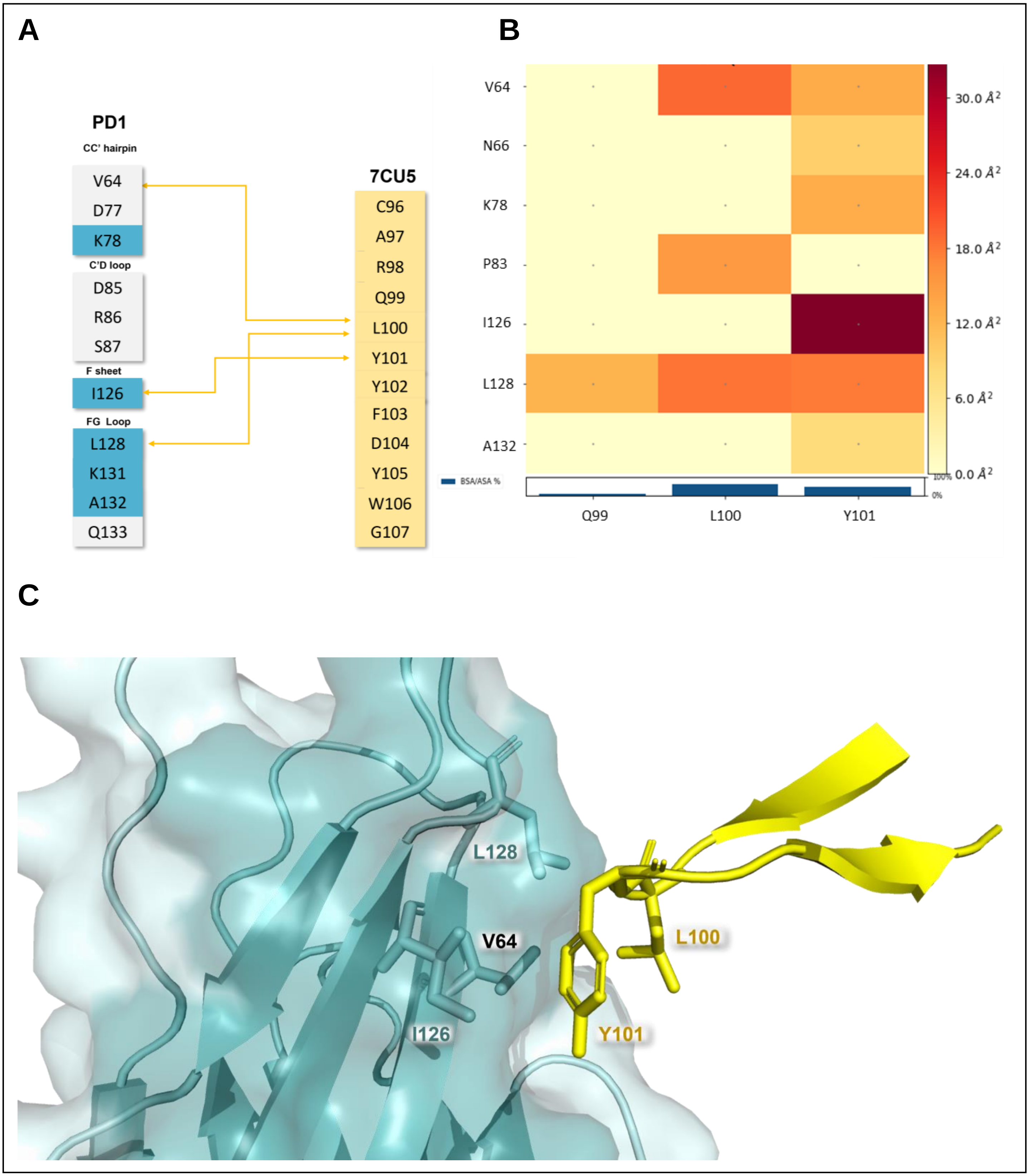

Figure S6. Mapping and Identification of **GY-14** CDR-H3 loop interactions with PD-1 (PDB code: 6J14). (**A**) Box diagram overview of contact interactions (red: salt bridges; blue: hydrogen bonds; green: water-mediated hydrogen bonds; yellow hydrophobic interactions). (**B**) Heat map of CDR-H3 residues involved at the contact surface area with PD-1. (**C**) Close-up view of CDR-H3 loop residues forming contact interactions with PD-1 (residues involved in hydrophobic interactions outlined in lighter color)

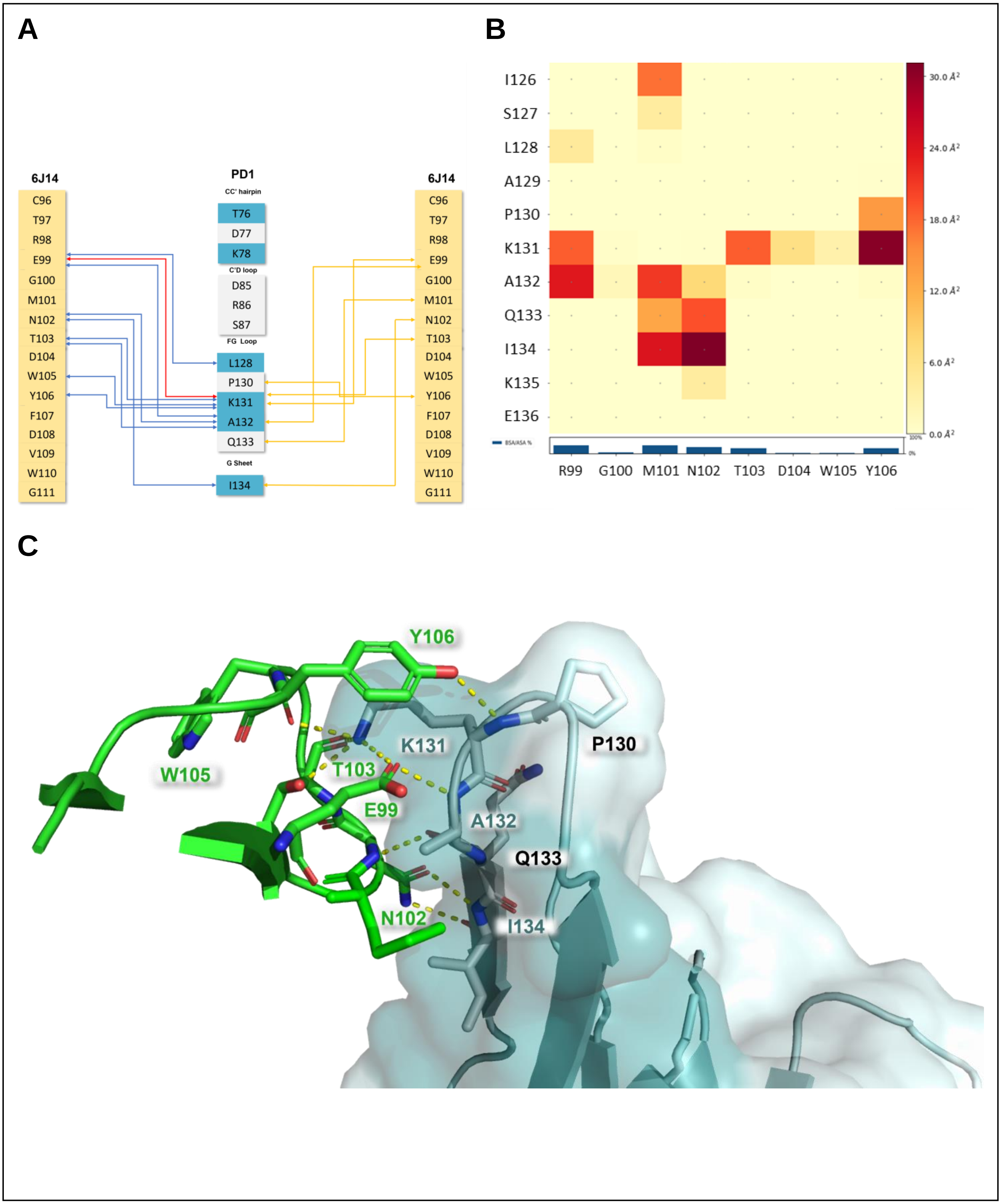

Figure S7. Mapping and Identification of **tislelizumab** CDR-H3 loop interactions with PD-1 (PDB code: 7BXA). (**A**) Box diagram overview of contact interactions (red: salt bridges; blue: hydrogen bonds; green: water-mediated hydrogen bonds; yellow hydrophobic interactions). (**B**) Heat map of CDR-H3 residues involved at the contact surface area with PD-1. (**C**) Close-up view of CDR-H3 loop residues forming contact interactions with PD-1 (residues involved in hydrophobic interactions outlined in lighter color)

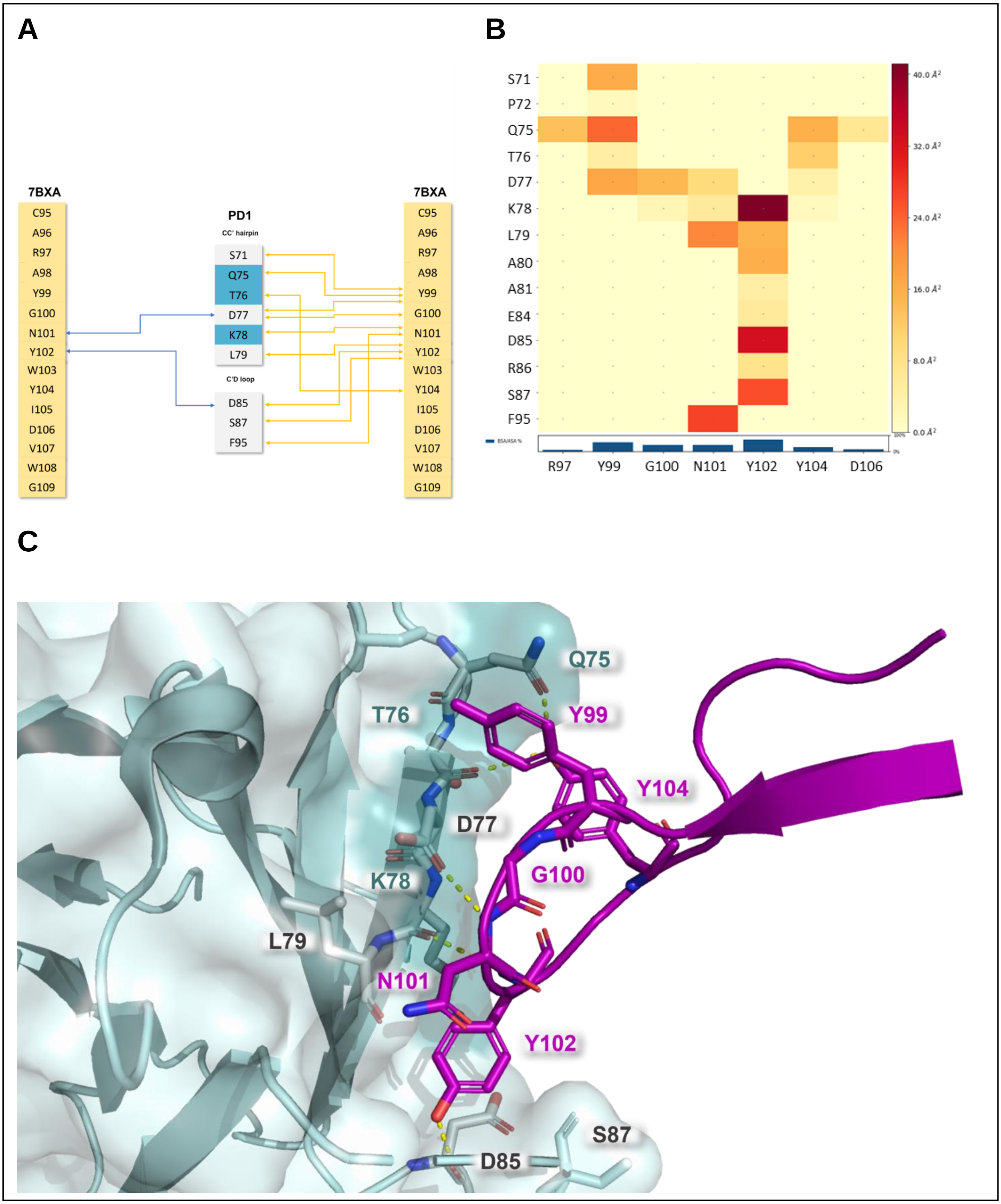

3. Structural Analysis Summary of anti-PD1 Blockers

a. *Structural Analysis Summary*

The crystal structure PDB files were imported into PyMOL to excise the full heavy chain, the light chain, and the CDR-H3 apex alone. The resulting complexes containing the heavy or light chain or CDR-H3 in contact with PD1 were saved as PDB and imported into dr_SASA for buried surface area calculation. The results of each were tabulated and the percent H3 loop binding surface area was calculated as a fraction of the total antibody binding surface area (TBSA). Six anti-PD1 antibodies were analyzed: pembrolizumab, GY-14, MW11-h317, tislelizumab, and camrelizumab.

Table S1. Selection of Potential PD-1 Inhibitors from known X-ray Crystal Structures of Antibody Drugs Complexes.

PD1•Ab (PDB code)	Pembrolizumab (5GGS)/(5B8C)	GY-14 (6J14)	MW11-h317 (6JJP)	mAB059c (6K0Y)	Tislelizumab (7BXA)	Camrelizumab (7CU5)
Avg BSA PD1:HC (Å^2)	715 / 682	513	570	545	327	445
Avg BSA PD1:LC (Å^2)	503 / 540	214	378	226	602	251
**TBSA** (Å^2)	1218 / 1222	727	948	771	929	696
Avg BSA PD1:CDR-H3 (Å^2)	**460 / 398**	**275**	217	204	**327**	156
% H3 loop binding surface over TBSA	**38% / 33%**	**38%**	23%	26%	**35%**	22%
# of H-Bond Contacts	9/6	9	2	2	2	0
# of Hydrophobic Contacts	12/10	6	0	8	10	3
Total Number of Contacts	21/16	15	2	10	12	3

b. *Binding Energy of CDR loops from the Rosetta Peptiderive Server and Potential Macrocyclic Scaffolds*

We then interrogated the Peptiderive server to analyze potential peptide binders of 8 to 12 amino acid in length (**Figures S8-S13**). In each case (except for camrelizumab) the heavy-chain CDR-H3 peptides were found to have the predominant binding to PD1 (see interface scores in **Table S2**).

Table S2. Calculated binding energies with macrocycle score from Peptiderive Server.

Antibody	Partner Energy (REU)	Position	Peptide Length	Interface Score	Sequence	Macrocycle (score)
**Tislelizumab**	**HC (-13.8)**	**98**	10	**-13.8**	**AYGNYWYIDV**	*NO*
	LC (-17.5)	50	10	-8.8	YAFHRFTGVP	*NO*
**Camrelizumab**	HC	51	8-12	-6.5	TISGGGANTYYP	TISGGGANTY (-5.1)
	HC	97	9-11	-7.1	CARQLYYFDYW	*NO*
	LC (-10.1)	211	9-11	**-8.1**	**QQVYSIPWTFGG**	QQVYSIPWTFG (-6.4)
**GY-14**	**HC (-20.1)**	**92**	9-12	**-13.1**	**REGMNTDWYFD**	TREGMNTDWYF (-13.1)
	LC (-8.4)	94	9-12	-4.7	QGSHVPYTF	QGSHVPYTF (-4.0)
**mAb059C**	**HC (-25.2)**	96	8-12	**-9.9**	**CARDLDIA**	ARDLDIATTRDYW (-9.7)
	LC	90	8-12	-7.8	QLDSYPRT	QLDSYPRTF (-7.2)
**MH11-h317**	**HC (-23.5)**	**96**	9-12	**-7.0**	**CASPDSSGVAY**	*NO*
	LC (-8.6)	88	9-12	-4.3	CQQYSRYPW	YCQQYSRYPWTF (-4.4)
**Pembrolizumab**	**HC (-28.1)**	**97**	8-12	**-17.0**	**RRDYRFDMGFY**	RRDYRFDMG (-17.3)
	LC (-8.4)	54	8-12	-4.6	LASYLESG	*NO*

Figure S8. Peptiderive generated figures for **Tislelizumab**.

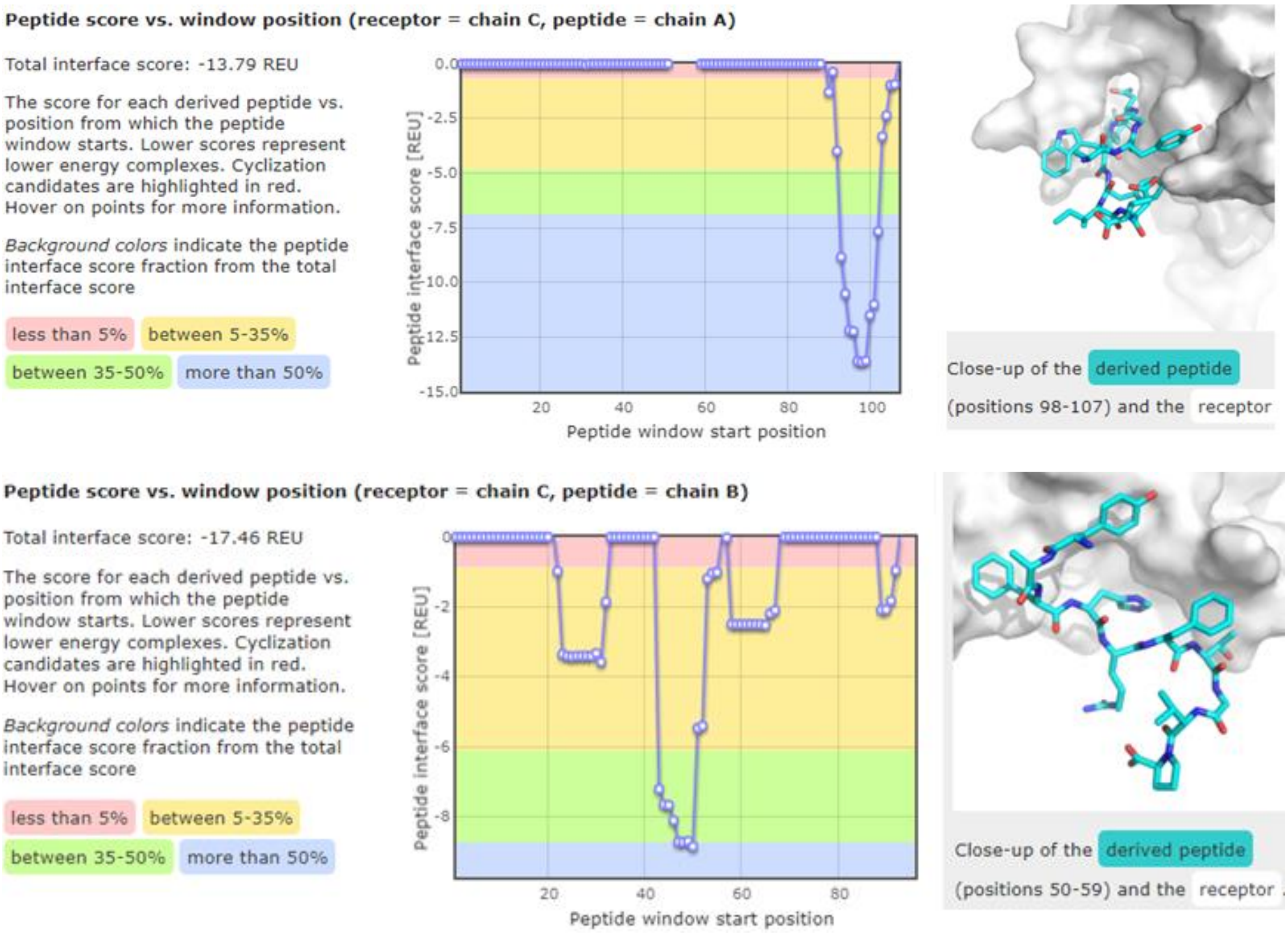

Figure S9. Peptiderive generated figures for **Camrelizumab**.

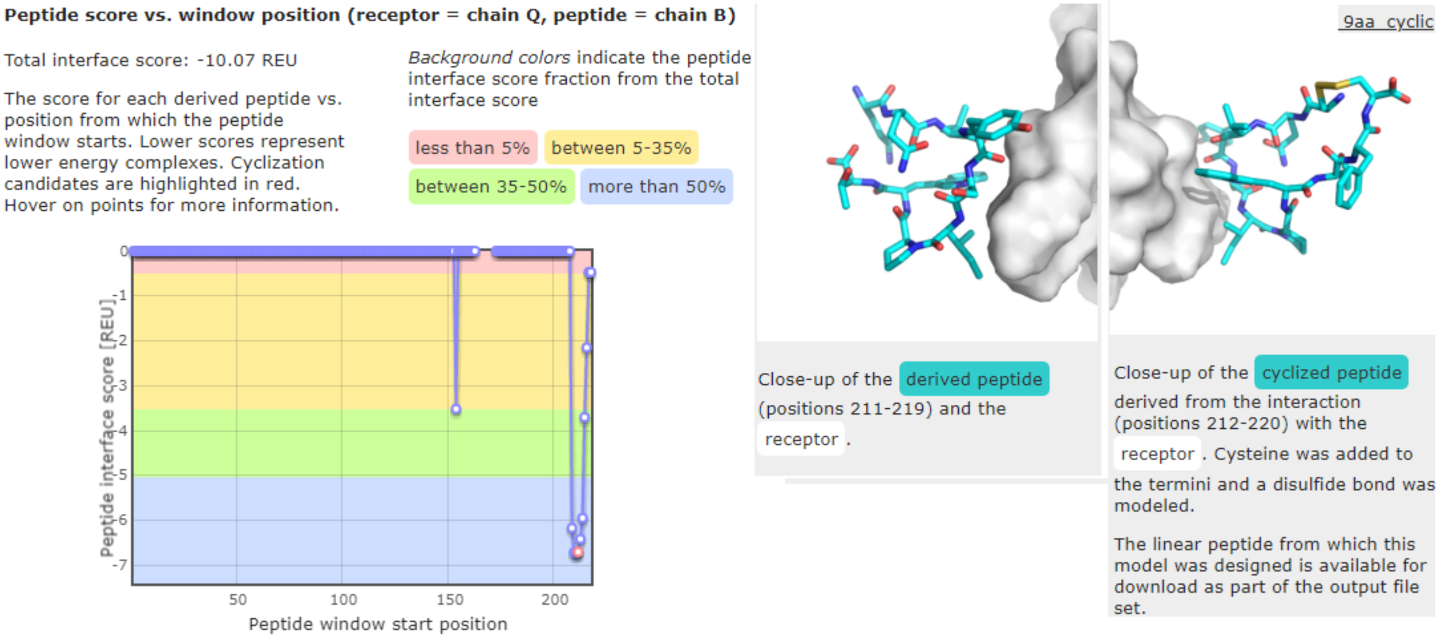

Figure S10. Peptiderive generated figures for **GY-14**.

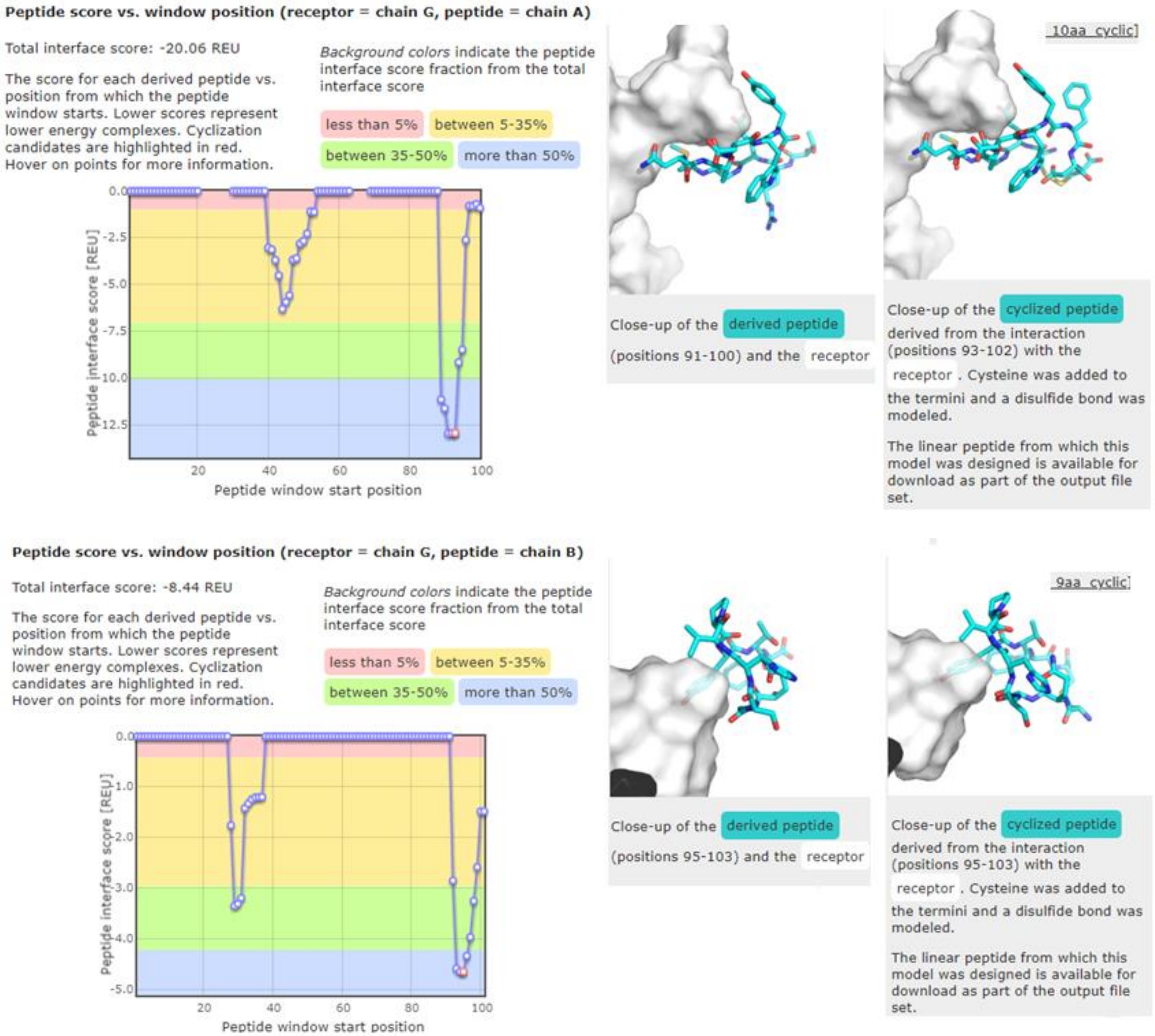

Figure S11. Peptiderive generated figures for **mAb059c**.

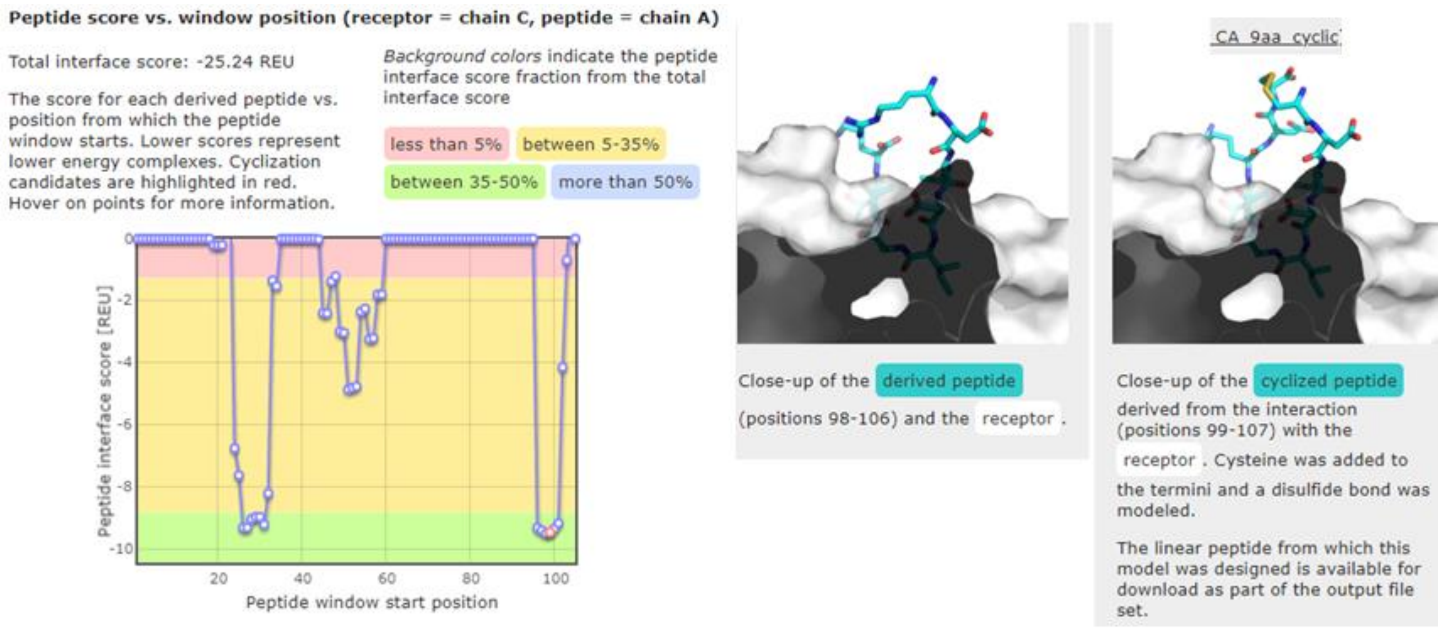

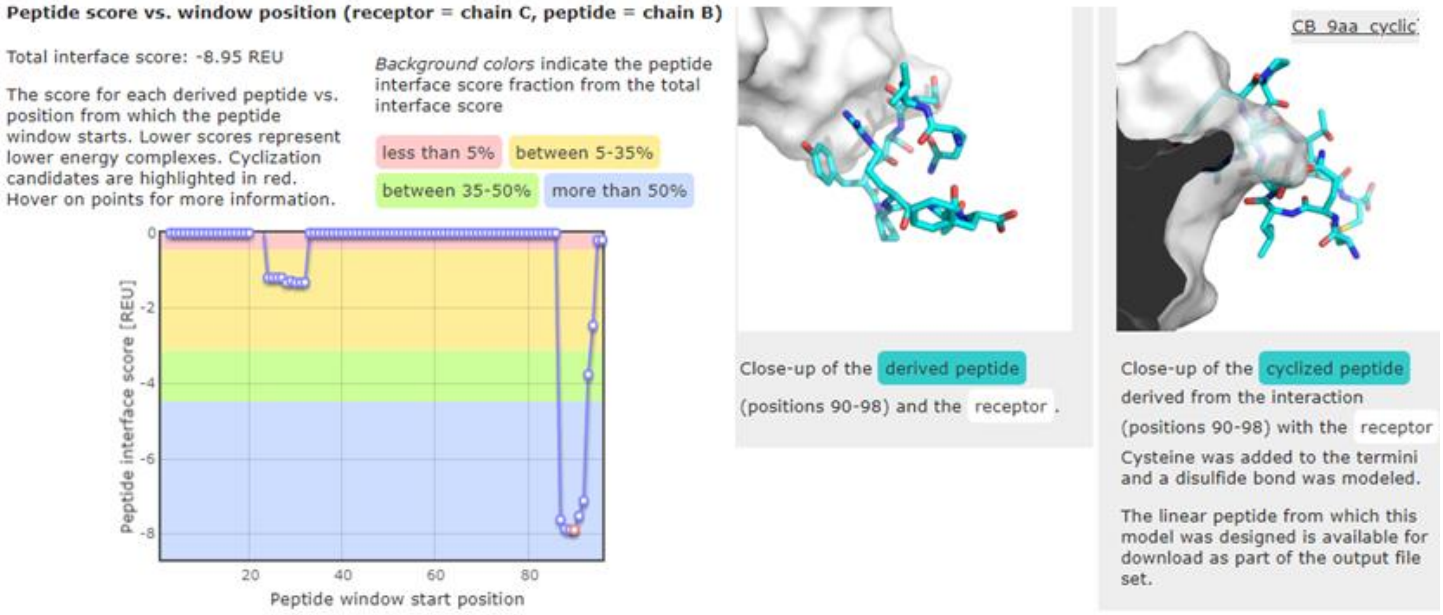

Figure S12. Peptiderive generated figures for **MH11-h317**.

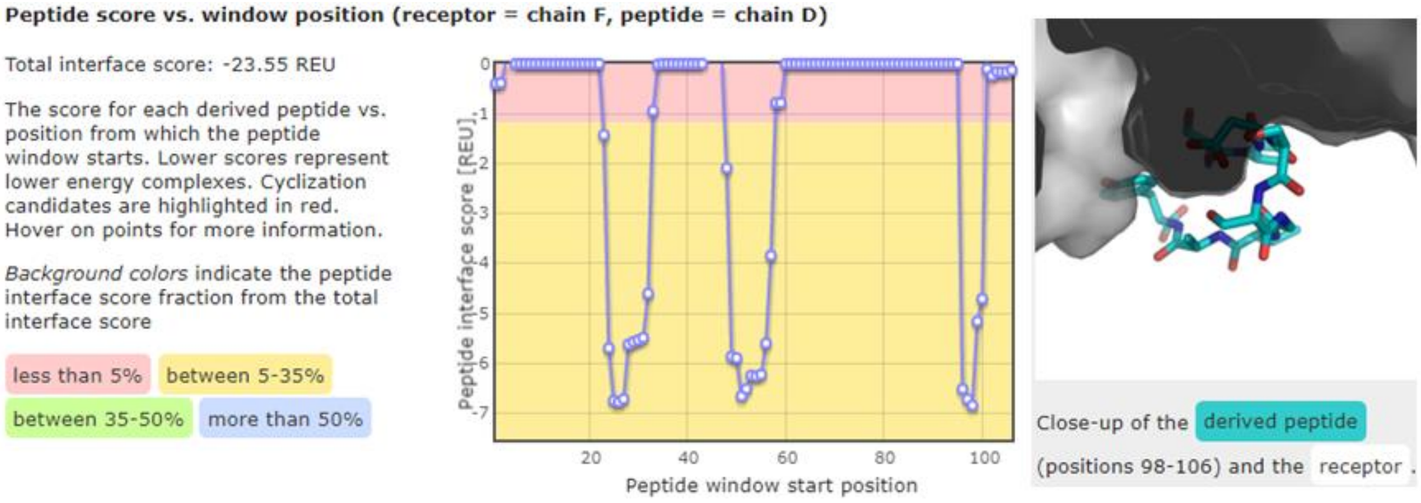

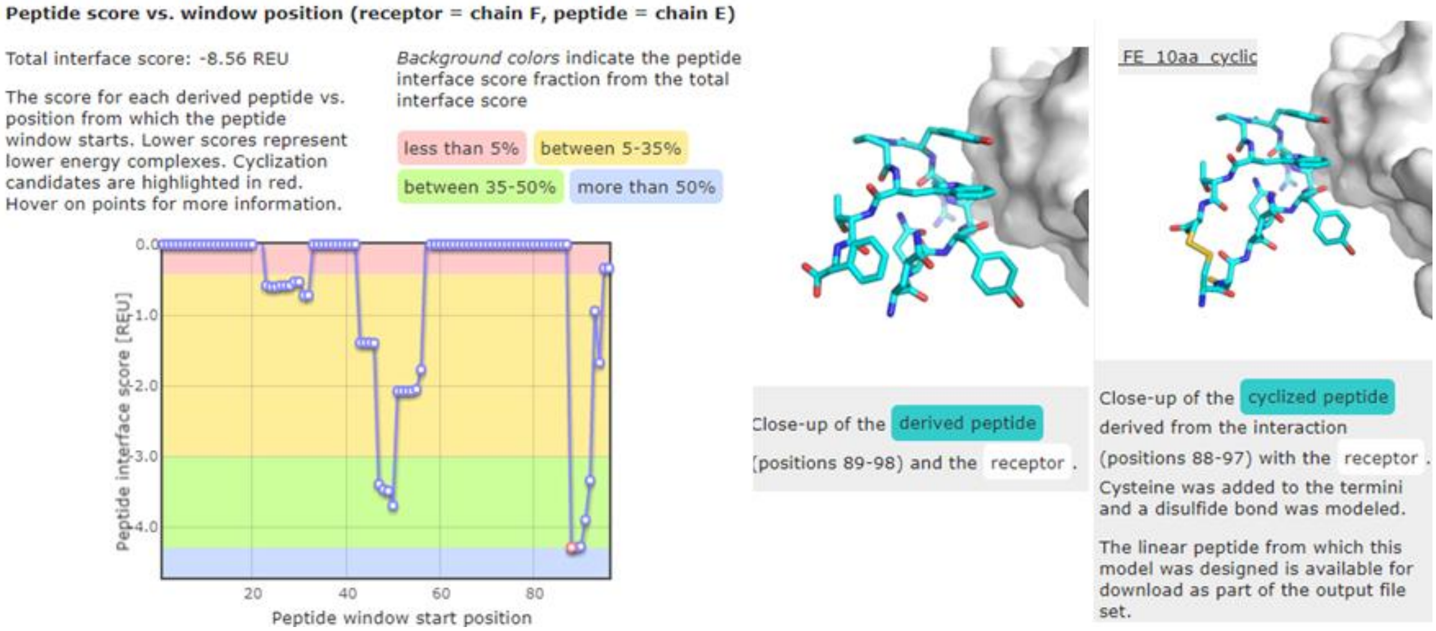

Figure S13. Peptiderive generated figures for **Pembrolizumab**.

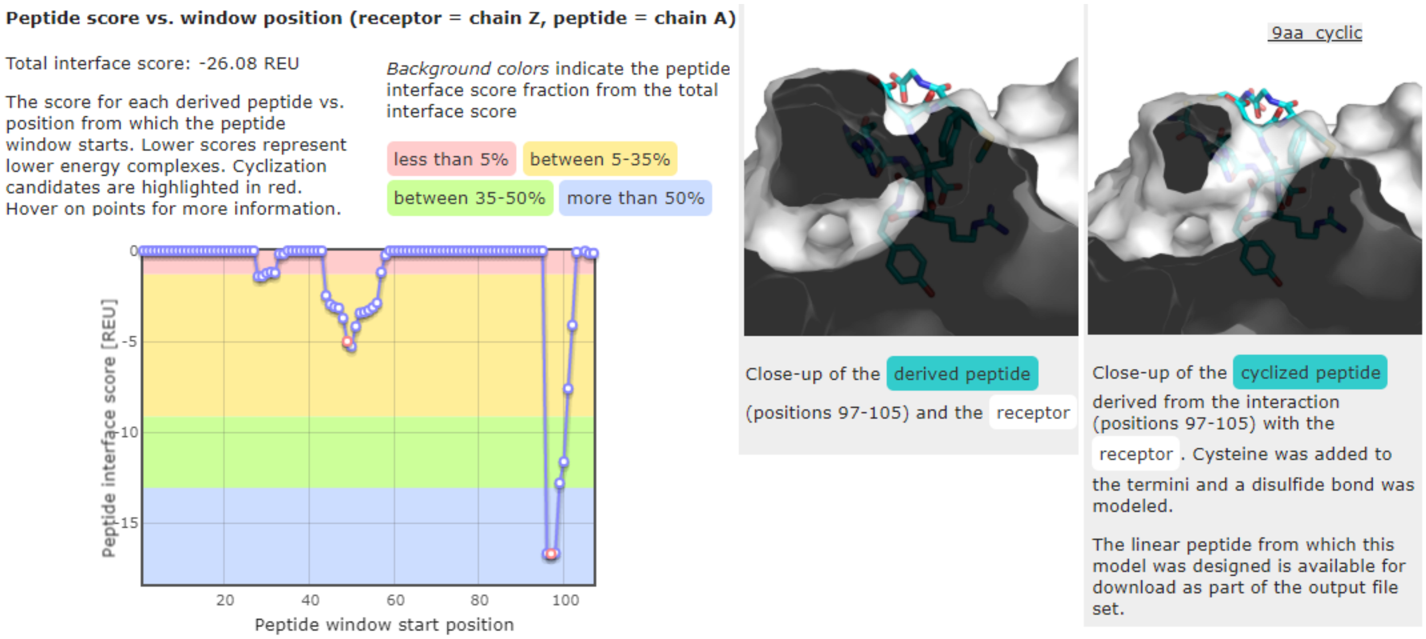

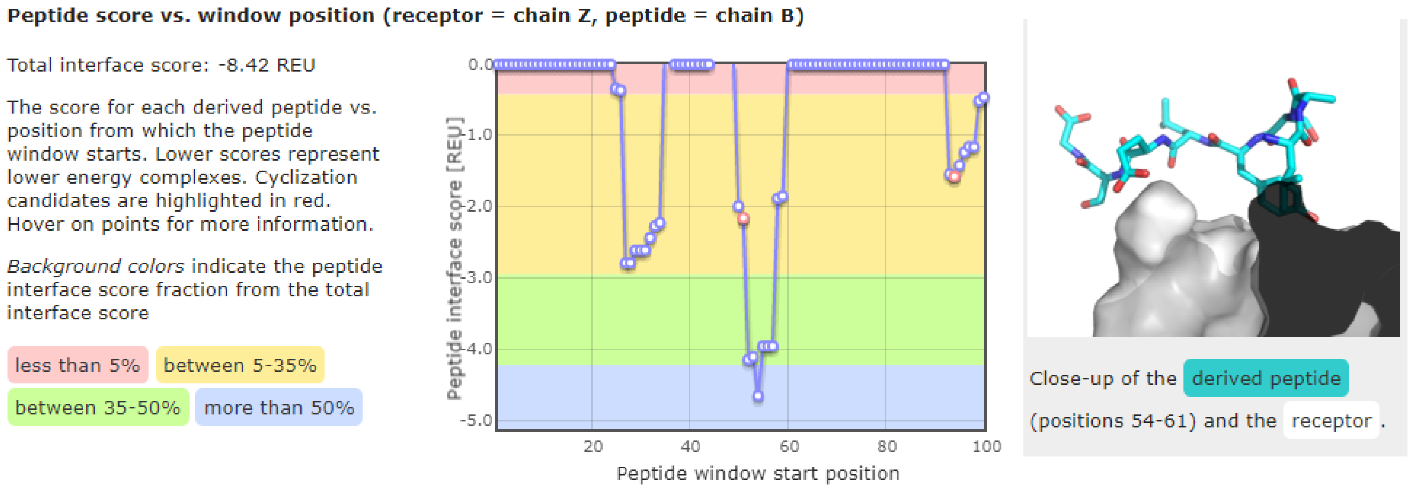

4. Lysine pKa Predictions

Using ROSIE (Rosetta Online Server that Includes Everyone)[SI-4] pKa values were calculated for the lysine residues of the extra cellular domain of PD1 in complex with the antibody from the crystal structures available mentioned previously.[SI-5] Each lysine pKa values was tabulated in **Table S3** and the results tend to indicate that the most buried lysines have lower pKas. The structural analysis also revealed that K131 is displayed in a much more flexible loop of PD1 (FG loop, **Figure S14**)

Table S3. Predicted lysine pKa values from crystal structure data imported to ROSIE server.

Residue pKa	**Pembrolizumab (5GGS)**	**Tislelizumab (7BXA)**	**GY-14 (6J14)**	**mAb-059 (6K0Y)**	**MW11-h317 (6JJP)**	**Camrelizumab (7CU5)**
K78	9.9	10.3	9.6	10.2	11.6	10
K131	10.5	N/A	10.7	10.3	10.4	10.6
K135	10.5	9.4	9.3	9.7	9.9	10.2

Figure S14. Motion of lysine residues (K78, K131 and K135) on the extracellular portion of PD1 based on antibody interactions. Overlay of PDBs: 5GGS, 7BXA, 6J14, 6K0Y, and 6JJP.

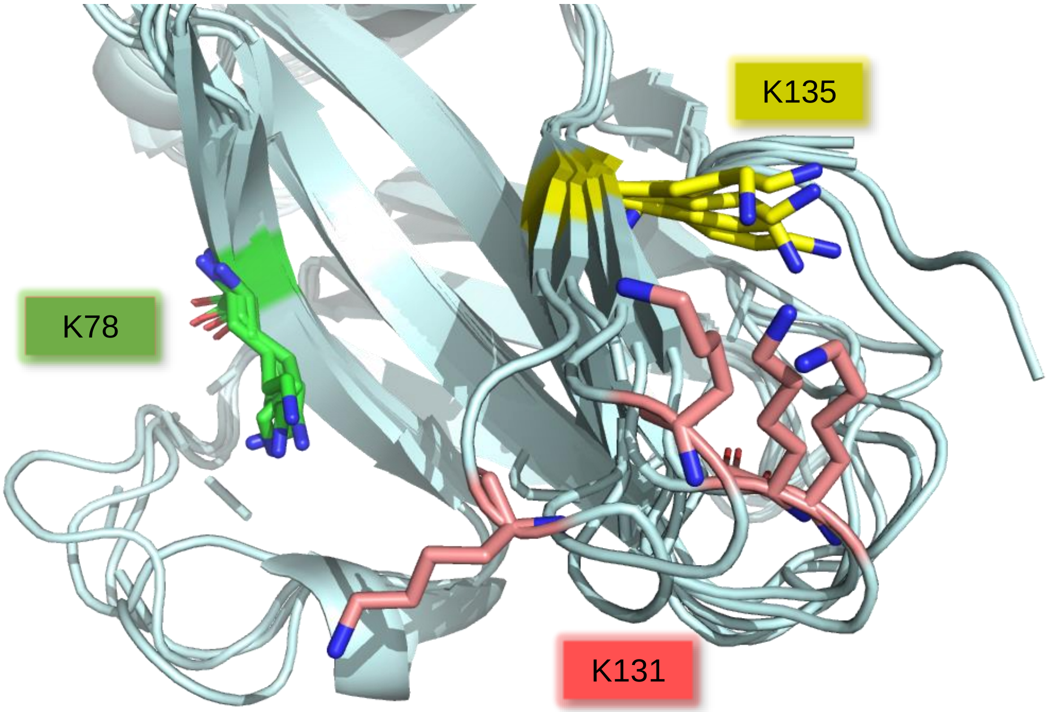

5. Lysine 131 Contact Interactions with GY-14 and Pembrolizumab CDR-H3 loops

A structure analysis was performed with an emphasis on Lysine 131 location in proximity to residues in the CDR-H3 loops of both pembrolizumab and GY-14. Pymol was used to calculate distances in the crystal structures between residues on PD1 and the CDR-H3 loop. PD1 is colored in grey, with surface showing. Blue shaded area of the surface is the PD1:PDL1 interface to show competitive inhibition. Pembrolizumab CDR-H3 loop is shown in orange and GY-14 CDR-H3 loop is colored green (**Figure S15**).

Figure S15. Identifying distances (within 4.0 Å) between (**A**) **pembrolizumab** M105 and $_{PD1}$K131 (PDB code: 5GGS) and **GY14** Y106 and $_{PD1}$K131 (PDB code: 6J14).

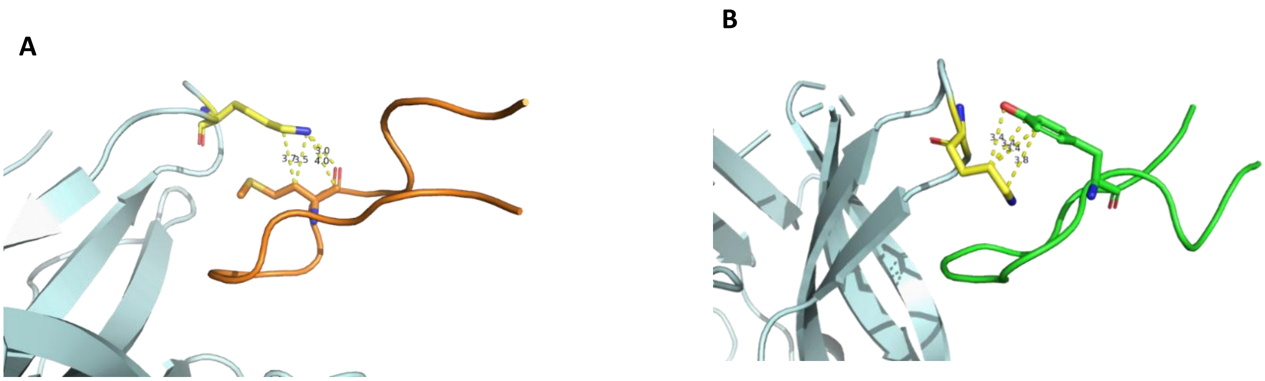

6. Peptide Synthesis, Purification and Structural Interpretation

a. Non-Proteinogenic Amino Acid Building Block Synthesis for Warhead Attachment

<u>Synthesis of Fmoc-Dap-OH and Fmoc-Dab-OH</u>

L-Fmoc-Asn-OH (10 g, 28.0 mmol, 1.0 eq) or L-Fmoc-Gln-OH (10 g, 28.0 mmol, 1.0 equiv.) was suspended in MeCN/H_2O (2:1 v/v, 144 mL). PIDA (18 g, 56 mmol, 2.0 equiv.) and pyridine (0.1 mL, 1.3 mmol, 0.05 equiv.) were added at once, and the reaction mixture was stirred for 24h at room temperature. The reaction was monitored by TLC (EtOAc/MeOH 9:1+1% AcOH v/v). After reaction completion, the solvent was evaporated under reduced pressure and the crude mixture washed with hot hexane (5 x 100 mL), and ice-cold EtOAc (2 x 10 mL) to yield a slightly orange solid (4.7 g, 14.4 mmol, 50%) with enough purity for the next step. $\boldsymbol{R_f}$ = 0.5 in MeOH for Fmoc-Dap-OH. $\boldsymbol{R_f}$ = 0.21 in EtOAc/MeOH (3:1) for Fmoc-Dab-OH.

<u>Synthesis of Fmoc-Dap(Mtt)-OH and Fmoc-Dab(Mtt)-OH</u>

L-Fmoc-Dap-OH (3.5 g, 11.0 mmol, 1.0 eq.) or L-Fmoc-Dab-OH (3.5 g, 11.0 mmol, 1.0 equiv.) was dissolved in anhydrous DMF (45 mL). The reaction mixture was cooled down to 0 °C. DIEA (5.8 mL, 33.0 mmol, 3.0 equiv.) was then added dropwise over 10 min at 0 °C, followed by dropwise addition over 20 min of a solution of Mtt-Cl (9.6 g, 33.0 mmol, 3.0 equiv.) dissolved in anhydrous DCM (45 mL) at the same temperature. The reaction mixture was then stirred for 1 h at 0 °C and the reaction was monitored by TLC until completion. MeOH (100 mL) was then added, and the reaction mixture heated and stirred at 50°C for 2 h. MeOH and other volatiles were evaporated and the crude product was then extracted twice with EtOAc/H_2O (1:5 v/v, 150 mL/750 mL). The organic layer was then washed with brine (1 x 100 mL), dried over Na_2SO_4, filtrated, then evaporated under reduced pressure to give a brown gel. The gel was then directly washed with hexane (10 X 30 mL) followed by cold ether (5 X 30 mL). The crude product was finally precipitated out from the combined ether/hexane phases to afford product in a pure form as a white solid (2.8 g, 5.0 mmol, 48% yield). Fmoc-Dap(Mtt)-OH can also be further purified by flash chromatography on silica, neutralized by a mixture of DCM/NH_4OH (99.5:0.5 v/v), and using a gradient solvent system (DCM / MeOH; 95:5 to 85:15). $\boldsymbol{R_f}$ = 0.3 (DCM / MeOH / NH_4OH 94.5:5:0.5 v/v/v) for Fmoc-Dap(Mtt)-OH. **$[\alpha]D_2O$** = + 14.2 (c 1.00, MeOH) for Fmoc-Dap(Mtt)-OH. $\boldsymbol{R_f}$ = 0.32 (DCM / MeOH / NH_4OH 90.5:9:0.5 v/v/v) for Fmoc-Dab(Mtt)-OH.

NMR Assignments Fmoc-Dap(Mtt)-OH

^{1}H NMR (400 MHz, CD_3OD, δ): 7.78 ($2H_{ArFmoc}$, d, J = 8Hz); 7.63 ($2H_{ArFmoc}$, dd, J = 8, 4 Hz); 7.44-7.24 ($16H_{ArMtt-Fmoc}$, m); 7.13 ($2H_{ArFmoc}$, d, J = 8 Hz); 4.31 ($2H_{CH2Fmoc}$, d, J = 8 Hz); 4.24 ($1H_{CHFmoc}$, t, J = 8 Hz); 4.19 ($1H_{\alpha}$, t, J = 8 Hz); 2.76 ($1H_{\beta}$, dd, J = 8, 12 Hz); 2.69 ($1H_{\beta}$, dd, J = 8, 12 Hz); 2.30 ($3H_{CH3Mtt}$, s).

^{13}C NMR (400 MHz, CD_3OD, δ): 175.2 (CO_2H); 158.4 (CO2N); 145.3 (C_{Fmoc}); 145.1 (C_{Fmoc}); 144.7 (C_{Mtt}); 142.5 ($2C_{Fmoc}$); 141.4 ($2C_{Mtt}$); 138.4 (C_{Mtt}); 129.9 ($2C_{Ar}$); 129.8 ($2C_{Ar}$); 129.7 ($6C_{Ar}$); 129.3 ($2C_{Ar}$); 128.8 ($2C_{Ar}$); 128.4 ($2C_{Ar}$); 128.2 ($2C_{Ar}$); 126.2 ($2C_{ArFmoc}$); 120.9 ($2C_{ArFmoc}$); 73.2 (C_{Mtt}); 68.1 (C_{H2Fmoc}); 54.4 (C_{α}); 48.3 (C_{HFmoc}); 46.3 (C_{β}); 20.9 (C_{H3Mtt}).

NMR Assignments Fmoc-Dab(Mtt)-OH

^{1}H NMR (400 MHz, CD_3OD, δ): 7.80 ($2H_{ArFmoc}$, d, J = 8Hz); 7.63 ($2H_{ArFmoc}$, dd, J = 8, 4 Hz); 7.46-7.22 ($16H_{ArMtt-Fmoc}$, m); 7.11 ($2H_{ArFmoc}$, d, J = 8 Hz); 4.30 ($2H_{CH2Fmoc}$, d, J = 8 Hz); 4.17 ($1H_{CHFmoc}$, t, J = 8 Hz); 4.11 ($1H_{\alpha}$, t, J = 8 Hz); 2.03 ($2H_{\beta}$, dt, J = 8, 12 Hz); 2.87 ($2H_{\gamma}$, t, J = 8, 12 Hz); 2.33 ($3H_{CH3Mtt}$, s).

^{13}C NMR (400 MHz, CD_3OD, δ): 174.8 (CO_2H); 156.9 (CO2N); 145.1 (C_{Fmoc}); 143.8(C_{Fmoc}); 143.4(C_{Mtt}); 142.5 ($2C_{Fmoc}$); 141.3 ($2C_{Mtt}$); 138.4 (C_{Mtt}); 129.9 ($2C_{Ar}$); 129.8 ($2C_{Ar}$); 129.7 ($6C_{Ar}$); 129.3 ($2C_{Ar}$); 128.8 ($2C_{Ar}$); 128.4 ($2C_{Ar}$); 126.6 ($2C_{Ar}$); 124.9 ($2C_{ArFmoc}$); 119.5 ($2C_{ArFmoc}$); 71.1 (C_{Mtt}); 66.9 (C_{H2Fmoc}); 54.3 (C_{α}); 47.0 (C_{HFmoc}); 42.4 (C_{γ}); 30.4 (C_{β}); 19.3 (C_{H3Mtt}).

b. Peptide Synthesis and Analysis.

General procedure for the synthesis *via* Fmoc-SPPS:

Peptides containing natural amino acids (*ADAPTins* **2a**, **2b**) were synthesized using standard Fmoc-chemistry on Wang resin (0.6 meq/g) using an automated PS3 peptide synthesizer. For each iteration, the deprotection/coupling sequence entails: (1) Wash of the resin with DMF (3 X 5.0 mL) for 0.5 min each, (2) Fmoc-deprotection run twice using an excess of piperidine in DMF (20% v/v, 5.0 mL) for 5 min each, (3) Wash with DMF (6 X 5.0 mL) for 0.5 min each, (4) The cocktail from the entire vial (described above) was dissolved with *N*-methylmorpholine in DMF (3.0 mL, 4 M) and added for a 40-min coupling, (5) Wash with DMF (3 X 5.0 mL) for 0.5 min each, finalized the sequence. After the final *N*-terminal coupling a final Fmoc-deprotection was achieved (step 2). Resulting peptides attached to the resin were washed with CH_2Cl_2 (2 X 10 mL), and the resin was dried under vacuum before storage under argon at -78 ^oC until cleavage.

Peptides containing unnatural amino acids for on-resin acrylamide warhead incorporation were synthesized in parallel on a shaker using standard Fmoc-chemistry on Wang resin (0.6 meq/g). Fmoc-protected proteinogenic α-amino acids (4 equiv.) were attached iteratively using HATU and DIPEA (4 equiv. each) through 2×10 min coupling cycles at 50 °C. Fmoc-orthogonally protected amino acids (**Figure S16**, residue (**X**): Fmoc-Dap(Mtt)-OH, Fmoc-Dab(Mtt)-OH, Fmoc-Dap(ivDde)-OH, or Fmoc-Dab(ivDde)-OH) were coupled when appropriate using the same reagents and equivalents through 2×30 min coupling cycles at 50 °C followed by a Kaiser test to determine the completion of the coupling. Specific Mtt and ivDde deprotections (b, d_1, or d_2; **Figure S16**) were achieved to introduce electrophilic warheads before the global peptide deprotection step and cleavage from solid support. Covalent reactive groups (CRGs) used in this study were acrylic acid (ACA), 4-(Dimethylamino)but-2-enoic acid (DMA), and methacrylic acid (MAA). For *N*-terminal Fmoc protected peptides, the selective Dap/Dab Mtt-deprotection was achieved with 1% TFA in DCM (2 x 10 min) at RT, while the Dap/Dab ivDde-deprotection was achieved with hydroxylamine hydrochloride (1 equiv.) and imidazole (0.75 equiv.) in *N*-methylpyrrolidone (NMP) for 3 x 3 h at 50 °C. For *N*-terminal Boc protected peptides the Dap/Dab ivDde-deprotection was achieved with 4% hydrazine in DMF (6 x 2 min) at RT. Once the linear peptides were synthesized and after orthogonal deprotection of Dap/Dab residues, acrylic acid derived fragments (ACA, DMA, and MAA) were coupled on resin using HATU (4 equiv.), DIPEA (4 equiv.) in DMF 2 x 30 min at 50 °C (c_1, c_2, or c_3; respectively, **Figure S16**).

Figure S16. Procedure for peptide synthesis and on-resin warhead incorporation *via* Fmoc-SPPS. For X, n=1 is Dap and n=2 is Dab. (*) is denoted for protecting groups.

Step	Reagents and Conditions
a	**SPPS:** a) 20% piperidine in DMF (2 x 1min); b) Fmoc-AA-OH (4 equiv), HOBT (4.4 equiv), HBTU (4.4 equiv), DIPEA (4 equiv), 2 x 10min under heat lamp
b	1% TFA in DCM, 2 x 10 min
c_1	acrylic acid (4 equiv), HATU (4 equiv), DIPEA (4 equiv) in DMF, 2 x 30 min
c_2	4-(Dimethylamino)but-2-enoic acid (4 equiv), HATU (4 equiv), DIPEA (4 equiv) in DMF, 2 x 30 min
c_3	methacrylic acid (4 equiv), HATU (4 equiv), DIPEA (4 equiv) in DMF, 2 x 30 min
d_1	TFA:TIS:H_2O (95:2.5:2.5), 1.5-2 h
d_2	TFA:Phenol:H_2O:thioanisole:EDT (82.5:5:5:5:2.5), 1.5-2 h, under argon
g_1	hydroxylamine hydrochloride (1 equiv.) and imidazole (0.75 equiv.) in N-methylpyrrolidone (NMP), shake 3 x 3 h at 50°C
g_2	4% hydrazine in DMF, 6 x 2 min

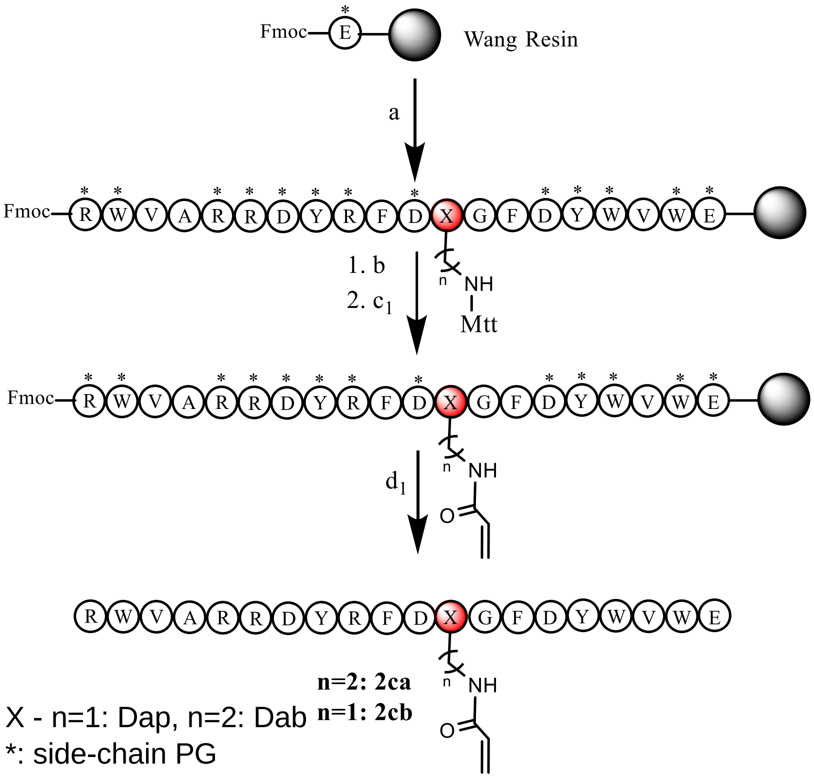

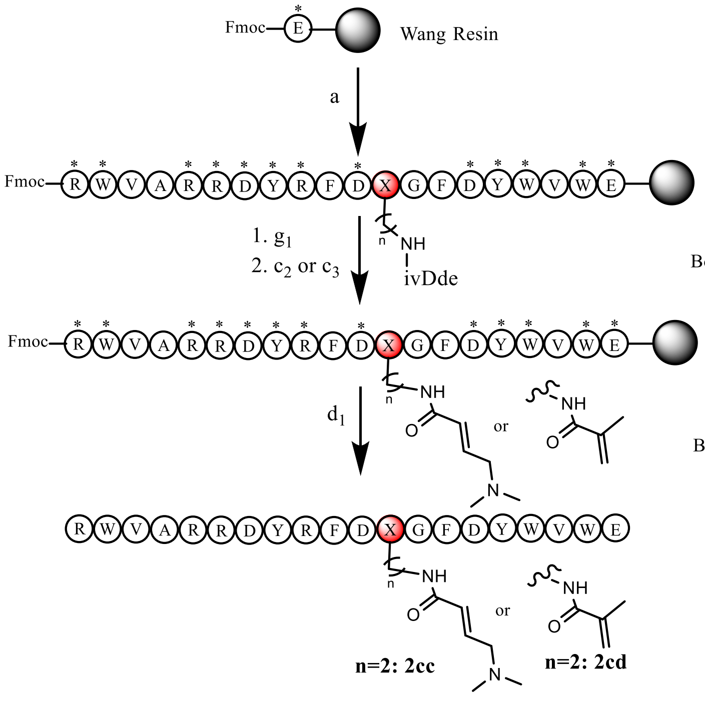

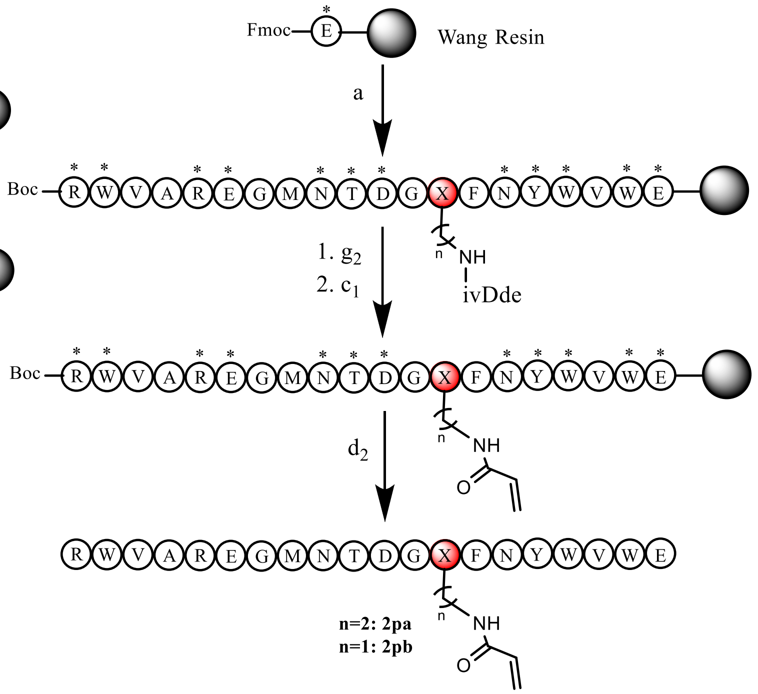

General procedure for the peptide cleavage off the resins: The same protocol of simultaneous resin cleavage and side-chains deprotection was usually applied, unless a *N*-terminal Fmoc was present then a Fmoc-removal with 20% piperidine in DMF was achieved before full cleavage. The dried resin was suspended in a cleavage cocktail (TFA/TIS/H_2O, 95:2.5:2.5 v/v, 1.0 mL per 20 mg of resin) and shaken for 1.5-2 h at rt. Peptides containing a methionine residue were cleaved with TFA:Phenol:H_2O:thioanisole:EDT (82.5:5:5:5:2.5), 1.5-2 h, under argon. The mixture was filtered to remove the resin and evaporated on a rotary evaporator. Crude peptides (~250 mg) were precipitated in cold ether (40 mL), then centrifuged, and washed with cold ether (3 X 40 mL). The resulting crude peptides were solubilized in water and lyophilized before being stored as dry powders under argon at -78 ^{o}C.

Peptide Samples Storage and Concentration. After RP-HPLC purification, peptides were lyophilized and stored as a powder at −78 °C and reconstituted, when necessary, in potassium phosphate buffer (15 mM, 6.5 pH) stock solutions of 0.5 mg/mL for subsequent analysis. The concentration in pure peptide was accurately determined by UV-absorbance measurements using a JASCO V-670 spectrophotometer using the combined molar absorptivity of Trp (ε_{280} = 5580 M^{-1} .cm^{-1} per Trp) and Tyr (ε_{280} = 1280 M^{-1} .cm^{-1} per Tyr) residues present in the sequences of *ADAPTins*.

c. Peptide characterization by MALDI-TOF MS and RP-HPLC.

All lyophilized crude peptides were analyzed by analytical reverse-phase high-performance liquid chromatography (RP-HPLC) on a Hitachi L-7000 series equipped with a XBridge BEH C_{18} column (130 Å, 10 µm, 4.6 mm x 250 mm). HPLC grade acetonitrile and deionized water, each containing 0.1% trifluoroacetic acid, were used as mobile phases for analytical and semi-preparative RP-HPLC. Each peptide was analyzed using a gradient from 10% to 50% of acetonitrile over 30 min with a flow rate of 1.0 mL/min at room temperature and a detection at 220 nm. Yields refer to chromatographically and spectroscopically pure compounds, unless otherwise stated. If required, peptides were purified with a Hitachi L-7000 series HPLC equipped with a semi-preparative XBridge BEH C_{18} column (130 Å, 10µm, 10 mm x 250 mm) stationary phase by scaling up the analytical conditions. Pure fractions of peptides were collected and analyzed by analytical RP-HPLC and mass spectrometry using a Microflex LRF matrix-assisted laser desorption ionization time-of-flight (MALDI-TOF) instrument.

Table S4. Summary of all peptides prepared in this study.

Id	Sequences	Chemical Formula	%-Yield	HPLC Purity [a]	m/z $[M+H]^+$	
					Calcd	Found
**RC**	RGVARRDYRGDPGFDYWHWE	$C_{115}H_{156}N_{36}O_{31}$	33	98 (18.8)	2538.18	2538.17
**Native**	RWCAR-RDYRFDMGFD-YWGWE	$C_{125}H_{163}N_{35}O_{31}S_2$	33	90 (22.7)	2715.18	2715.31
**1a**	RWVW-GGGGKKGGGG-WVWE	$C_{93}H_{127}N_{27}O_{21}$	70	98 (17.3)	1957.97	1958.41
**1b**	RWVW-RDYRGDMGFD-WVWE	$C_{116}H_{151}N_{31}O_{28}S$	35	99 (23.3)	2459.11	2459.39
**1c**	RWVW-RDYRFDMGFD-WVWE	$C_{123}H_{157}N_{31}O_{28}S$	30	93 (24.5)	2549.16	2549.60
**1d**	RWVW-RRDYRFDMGFD-YWVWE	$C_{138}H_{178}N_{36}O_{31}S$	53	90 (23.7)	2868.33	2868.26
**2a**	RWVA-RGGGGKKGGFD-YWVWE	$C_{109}H_{151}N_{31}O_{26}$	51	99 (19.4)	2310.14	2310.77
**2b**	RWVA-RRDYRGDMGFD-YWVWE	$C_{123}H_{167}N_{35}O_{31}S$	34	99 (21.5)	2663.24	2663.64
**2c**	RWVA-RRDYRFDMGFD-YWVWE	$C_{130}H_{173}N_{35}O_{31}S$	36	99 (23.1)	2753.28	2753.87
**2d**	RWVA-RRDYRGDMGFD-YWHWE	$C_{124}H_{165}N_{37}O_{31}S$	65	95 (20.3)	2701.23	2701.94
**2e**	RWVAR-RDYRFDMGFD-YWHWE	$C_{131}H_{171}N_{37}O_{31}S$	n.a.	97 (21.5)	2791.27	2791.74
**2f**	RWVKR-RDYRGDM..GFD-YWVWE	$C_{126}H_{174}N_{36}O_{31}S$	31	97 (19.5)	2720.29	2720.61
**2g**	RWVKR-RDYRFDMGFD-YWVWE	$C_{133}H_{180}N_{36}O_{31}S$	n.a.	99 (21.9)	2810.34	2811.21
**2h**	RWVVR-RDYRGDMGFD-YWVWE	$C_{125}H_{171}N_{35}O_{31}S$	n.a.	99 (20.9)	2691.26	2691.35
**2i**	RWVVR-RDYRFDMGFD-YWVWE	$C_{132}H_{177}N_{35}O_{31}S$	n.a.	97 (23.1)	2781.31	2781.13
**2j**	RWVAR-RDYRGDMGFN-YWVWE	$C_{123}H_{168}N_{36}O_{30}S$	32	99 (20.9)	2662.25	2663.15
**2k**	RWVAR-RDYRFDMGFN-YWVWE	$C_{130}H_{174}N_{36}O_{30}S$	n.a.	96 (22.6)	2752.30	2752.28
**2l**	cRWCAR-RDYRFDMGFD-YWCWE	$C_{126}H_{163}N_{35}O_{31}S_3$	16	99 (22.0)	2759.14	2759.63
**1e**	RWVW-SPDSSGVAY-WVWE	$C_{102}H_{132}N_{24}O_{26}$	43	99 (22.8)	2109.98	2110.68
**1f**	RWVW-RDLDIATTRDY-WVWE	$C_{121}H_{168}N_{32}O_{31}$	28	93 (23.5)	2566.26	2566.88
**2m**	RWVAR-QLY...YFD-YWVWE	$C_{114}H_{146}N_{26}O_{25}$	n.a.	99 (25.2)	2280.10	2280.44
**2n**	RWVA-REGMNTDWYFD-YWHWE	$C_{130}H_{163}N_{33}O_{33}S$	10	94 (23.3)	2746.18	2747.46
**2o**	RWVA-REGMNTDGYFD-YWHWE	$C_{121}H_{156}N_{32}O_{33}S$	33	98 (21.1)	2617.12	2617.96
**2p**	RWVA-REGMNTDGYFN-YWVWE	$C_{120}H_{159}N_{31}O_{32}S$	n.a.	95 (21.2)	2579.84	2579.25
**2q**	RWVA-RAYGNYWYID-VWHWE	$C_{126}H_{160}N_{32}O_{28}$	23	99 (24.1)	2570.21	2570.07
**2r**	RWVA-RAYGNYGYID-VWHWE	$C_{117}H_{153}N_{31}O_{28}$	19	98 (21.9)	2441.18	2441.51
**2ca**	RWVAR-RDYRFD[Dab(ACA)]GFD-YWVWE	$C_{132}H_{174}N_{36}O_{32}$	9	99 (20.0)	2777.06	2777.51
**2cb**	RWVAR-RDYRFD[Dap(ACA)]GFD-YWVWE	$C_{131}H_{172}N_{36}O_{32}$	4	99 (22.5)	2764.04	2764.44
**2cc**	RWVAR-RDYRFD[Dab(DMA)]GFD-YWVWE	$C_{135}H_{181}N_{37}O_{32}$	20	99 (20.6)	2834.16	2834.74
**2cd**	RWVAR-RDYRFD[Dab(MAA)]GFD-YWVWE	$C_{133}H_{176}N_{36}O_{32}$	14	99 (19.7)	2791.09	2790.62
**2pa**	RWVA-REGMNTDG[Dab(ACA)]FN-YWVWE	$C_{118}H_{160}N_{32}O_{32}S$	22	99 (20.7)	2570.83	2570.42
**2pb**	RWVA-REGMNTDG[Dap(ACA)]FN-YWVWE	$C_{117}H_{158}N_{32}O_{32}S$	19	99 (22.1)	2556.80	2556.15

[a] Determined by integration of peak detected by UV-absorbance at 220 nm, from the purified lyophilized peptide samples.

Figure S17. Peptides' HPLC chromatograms.

Peptide **RC**: RGVA-RRDYRGDPGFDY-WHWE (300 mg, 0.13 mmol of Wang resin was used)
HPLC: Peptide RC retention time t_R = 18.83 min with a purity of 98%.

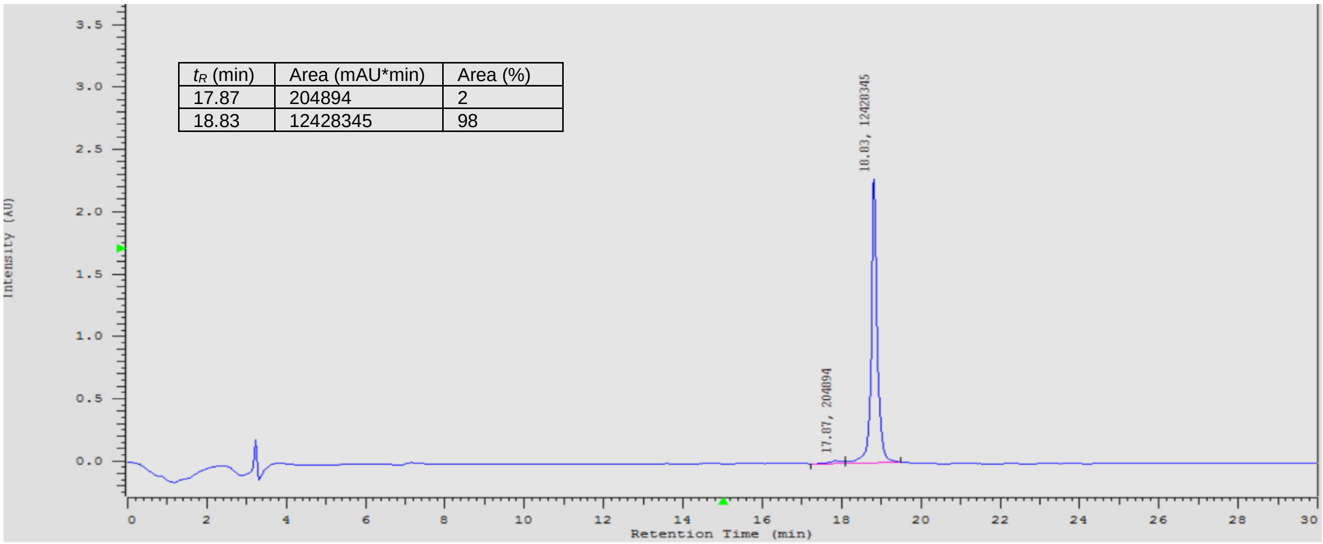

t_R (min)	Area (mAU*min)	Area (%)
17.87	204894	2
18.83	12428345	98

Peptide **Native**: RWCAR-RDYRFDMGFD-YWGWE (300 mg, 0.13 mmol of Wang resin was used)
HPLC: Peptide Native retention time t_R = 22.7 min with a purity of 90%.

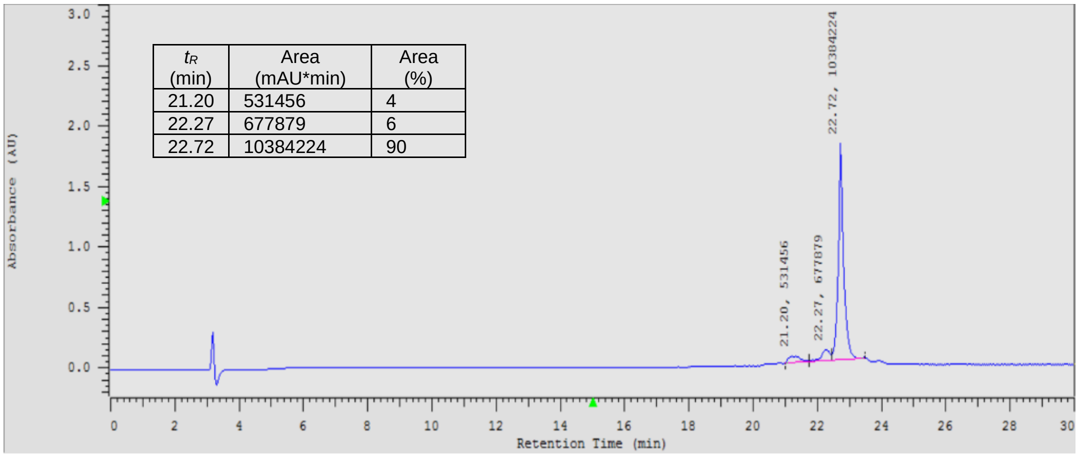

t_R (min)	Area (mAU*min)	Area (%)
21.20	531456	4
22.27	677879	6
22.72	10384224	90

Peptide **1a**: RWVW-GGGGKKGGGG-WVWE (300 mg, 0.13 mmol of Wang resin was used)
HPLC: Peptide **1a** retention time t_R = 17.25 min with a purity of 88%.

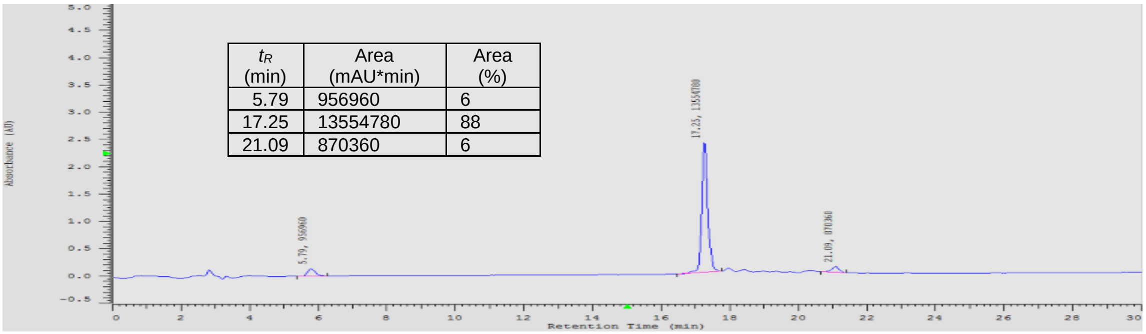

t_R (min)	Area (mAU*min)	Area (%)
5.79	956960	6
17.25	13554780	88
21.09	870360	6

Peptide **1b**: RWVW-RDYRGDMGFD-WVWE (300 mg, 0.13 mmol of Wang resin was used)
HPLC: Peptide **1b** retention time t_R = 23.2 min with a purity of 99%.

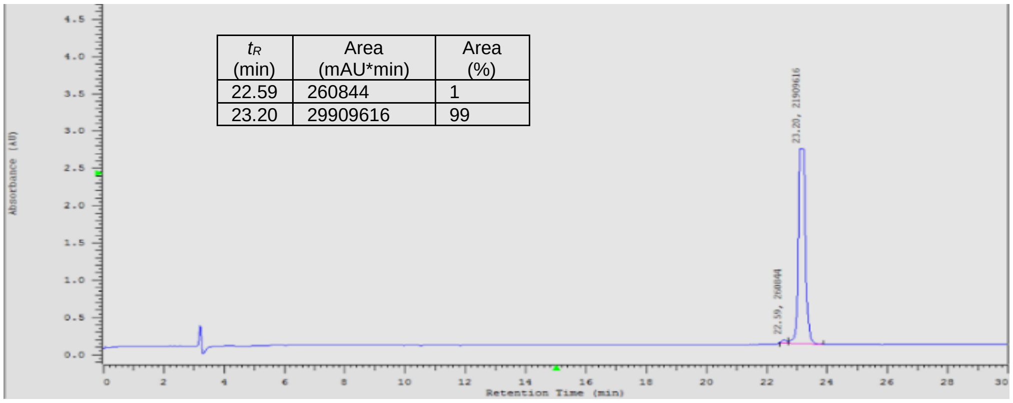

t_R (min)	Area (mAU*min)	Area (%)
22.59	260844	1
23.20	29909616	99

Peptide **1c**: RWVW-RDYRFDMGFD-WVWE (200 mg, 0.09 mmol of Wang resin was used)
HPLC: Peptide **1c** retention time t_R = 24.5 min with a purity of 93%.

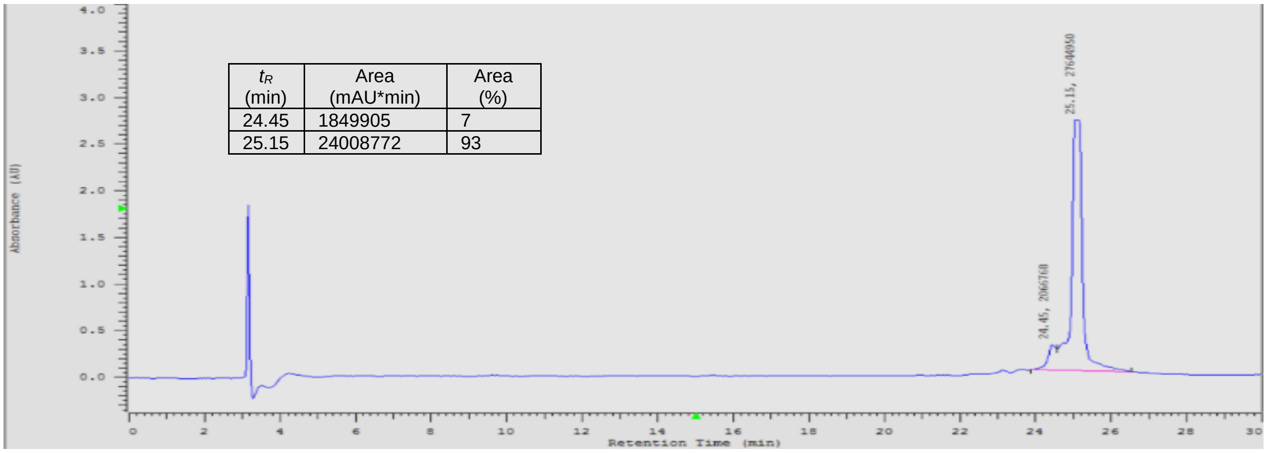

t_R (min)	Area (mAU*min)	Area (%)
24.45	1849905	7
25.15	24008772	93

Peptide **1d**: RWVW-RRDYRFDMGFD-YWVWE (300 mg, 0.13 mmol of Wang resin was used)
HPLC: Peptide **1d** retention time t_R = 23.68 min with a purity of 90%.

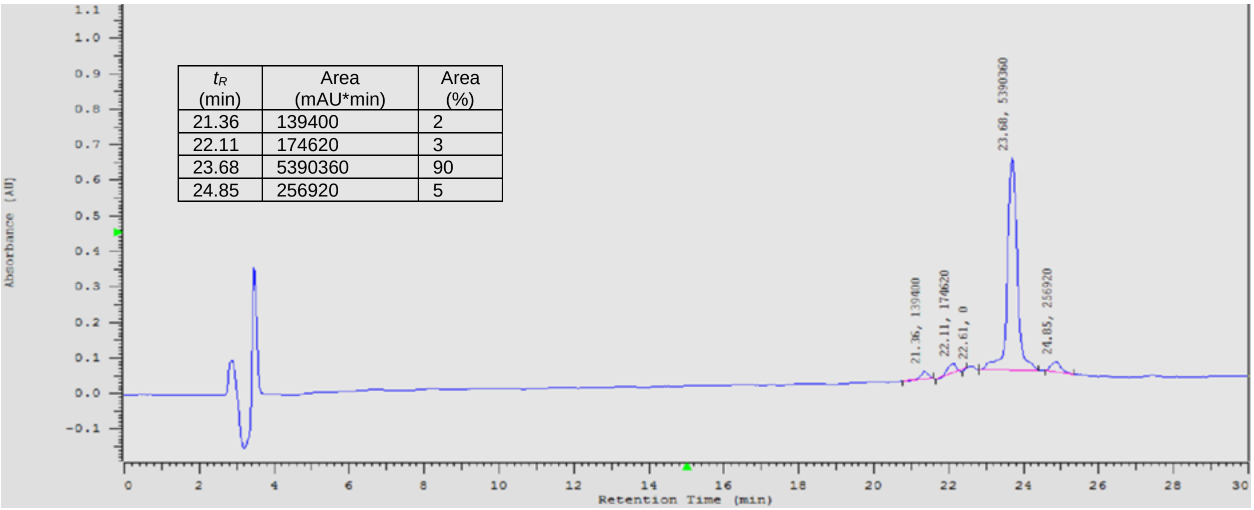

t_R (min)	Area (mAU*min)	Area (%)
21.36	139400	2
22.11	174620	3
23.68	5390360	90
24.85	256920	5

Peptide **2a**: RWVA-RGGGGKKGGFD-YWVWE (300 mg, 0.13 mmol of Wang resin was used)
HPLC: Peptide **2a** retention time t_R = 19.44 min with a purity of 99%.

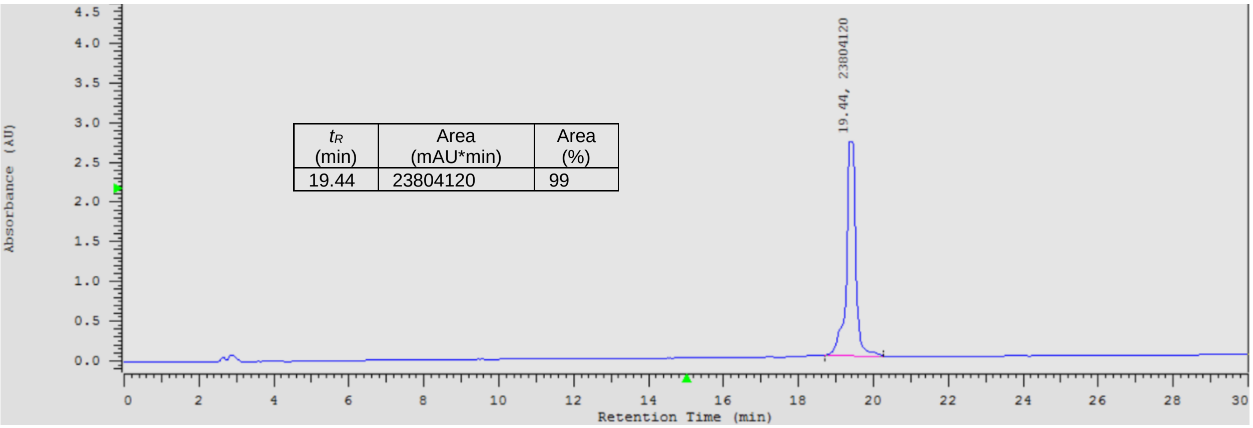

t_R (min)	Area (mAU*min)	Area (%)
19.44	23804120	99

Peptide **2b**: RWVA-RRDYRGDMGFD-YWVWE (300 mg, 0.13 mmol of Wang resin was used)
HPLC: Peptide 2b retention time t_R = 21.47 min with a purity of 99%.

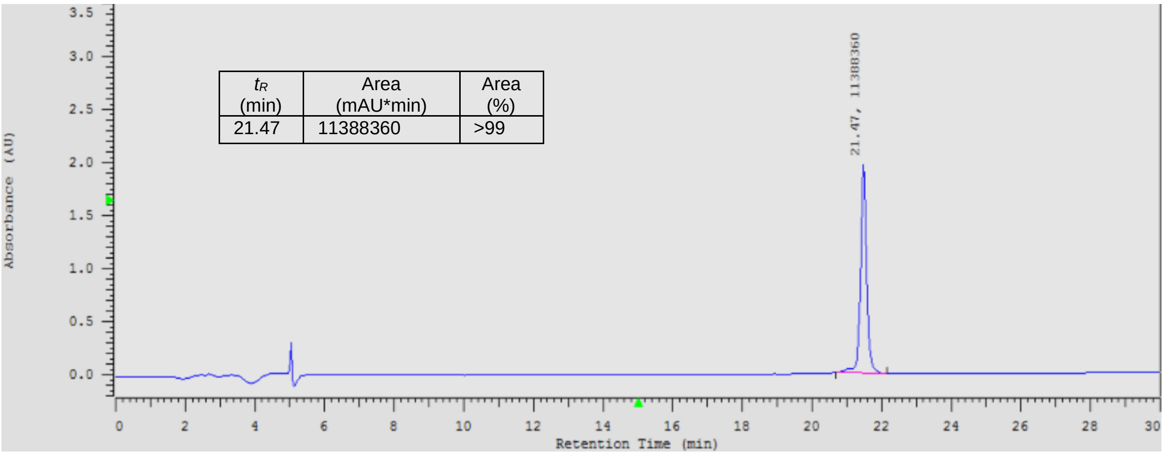

t_R (min)	Area (mAU*min)	Area (%)
21.47	11388360	>99

Peptide **2c**: RWVA-RRDYRFDMGFD-YWVWE (300 mg, 0.13 mmol of Wang resin was used)
HPLC: Peptide **2c** retention time t_R = 23.12 min with a purity of 99%.

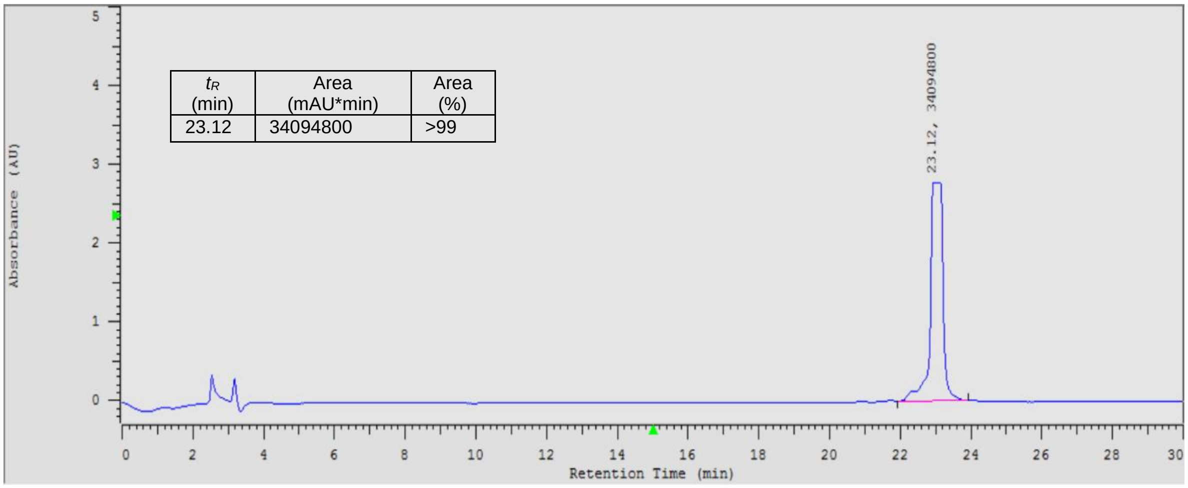

t_R (min)	Area (mAU*min)	Area (%)
23.12	34094800	>99

Peptide **2d**: RWVA-RRDYRGDMGFD-YWHWE (300 mg, 0.13 mmol of Wang resin was used)
HPLC: Peptide **2d** retention time t_R = 20.3 min with a purity of 95%.

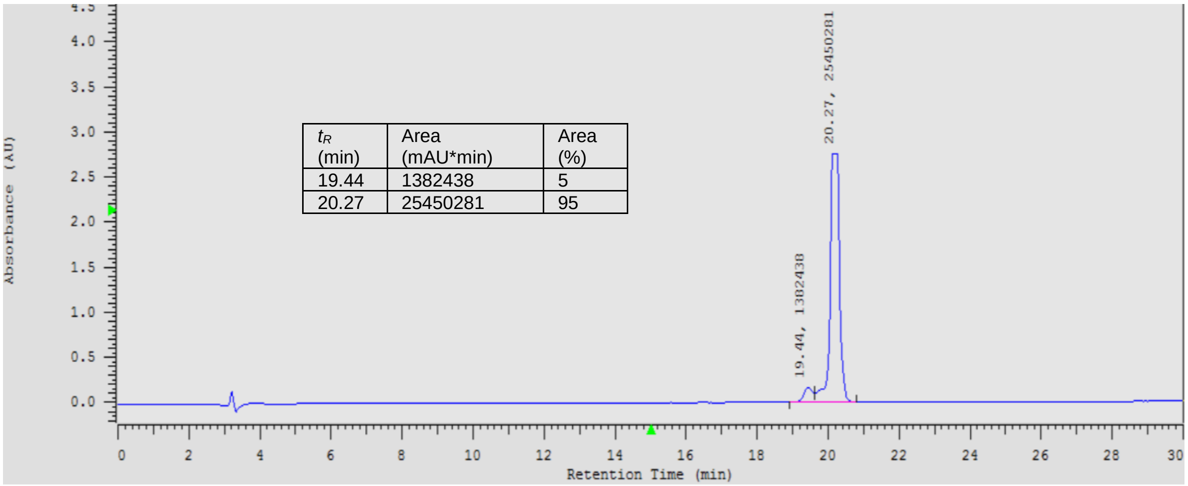

Peptide **2e**: RWVAR-RDYRFDMGFD-YWHWE (300 mg, 0.13 mmol of Wang resin was used)
HPLC: Peptide **2e** retention time t_R = 21.47 min with a purity of 99%.

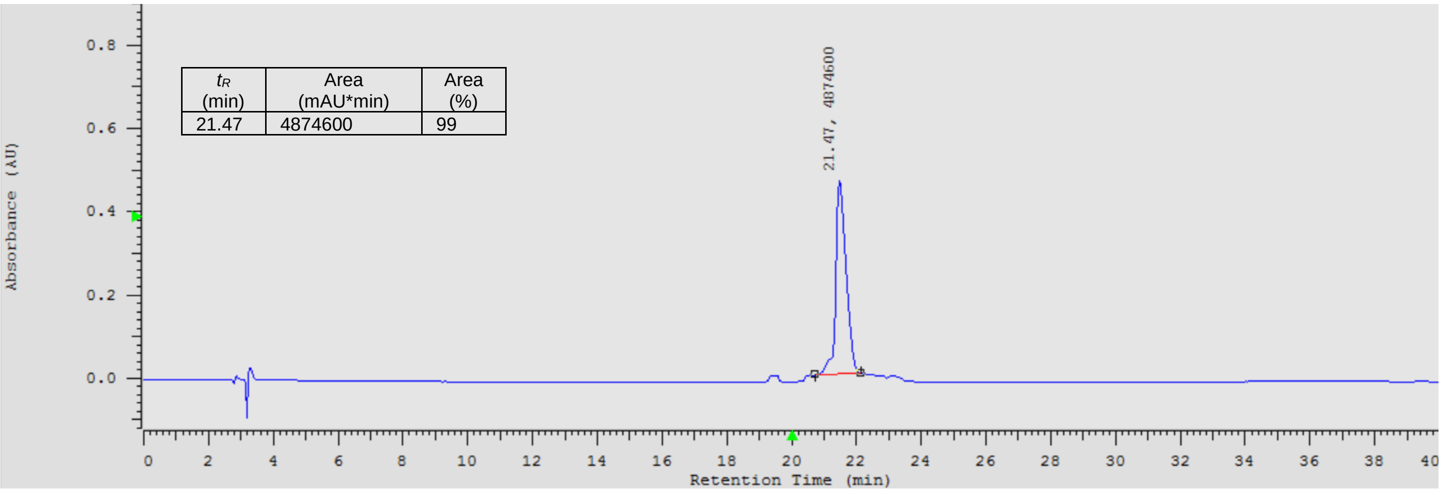

Peptide **2f**: RWVKR-RDYRGDMGFD-YWVWE (300 mg, 0.13 mmol of Wang resin was used)
HPLC: Peptide **2f** retention time t_R = 19.76 min with a purity of 97%.

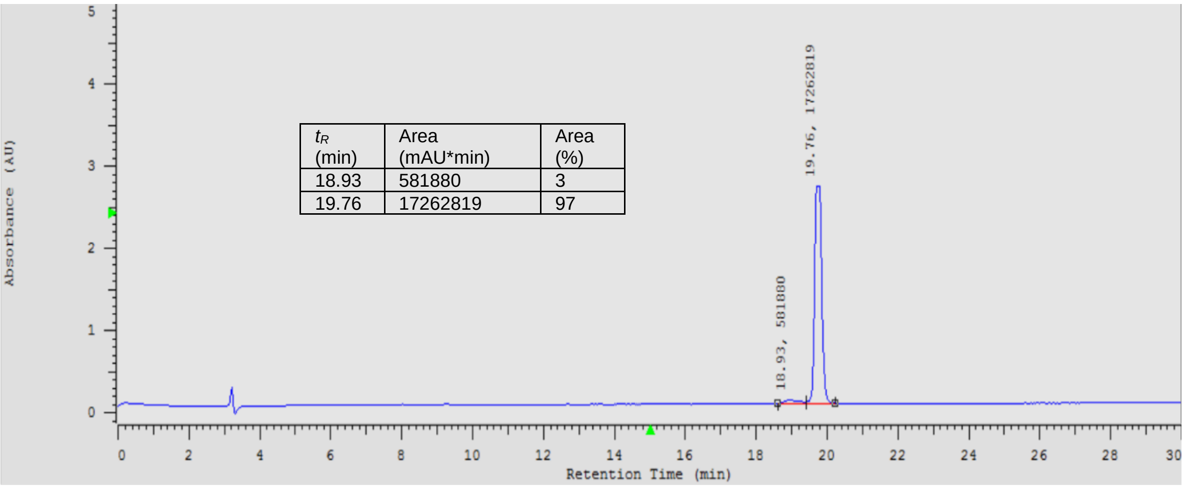

Peptide **2g**: RWVKR-RDYRFDMGFD-YWVWE (Ordered from Peptide 2.0)
HPLC: Peptide **2g** retention time t_R = 21.87 min with a purity of 99%.

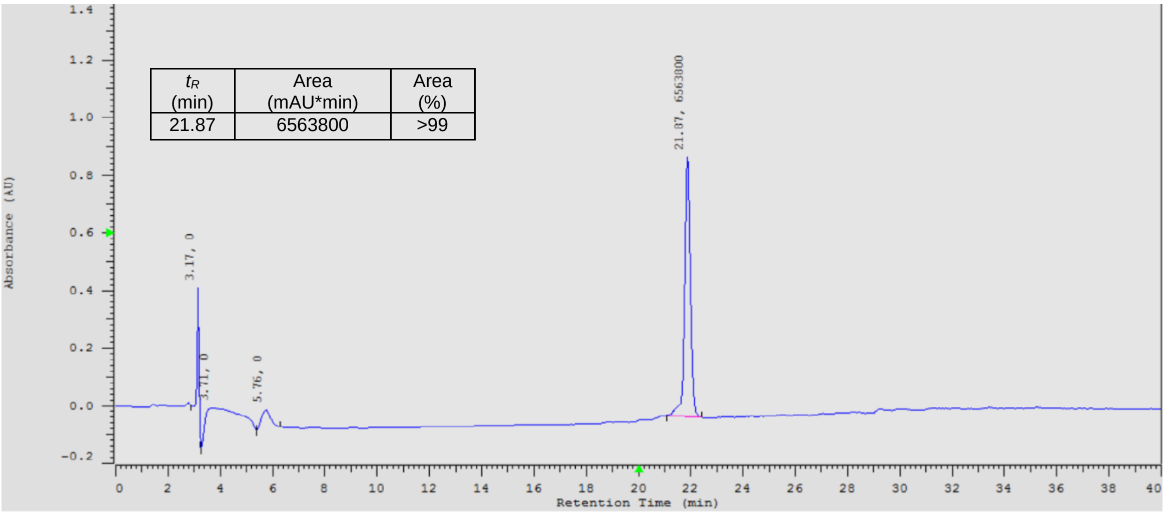

Peptide **2h**: RWVVR-RDYRGDMGFD-YWVWE (Ordered from Peptide2.0)
HPLC: Peptide **2h** retention time t_R = 20.91 min with a purity of 99%.

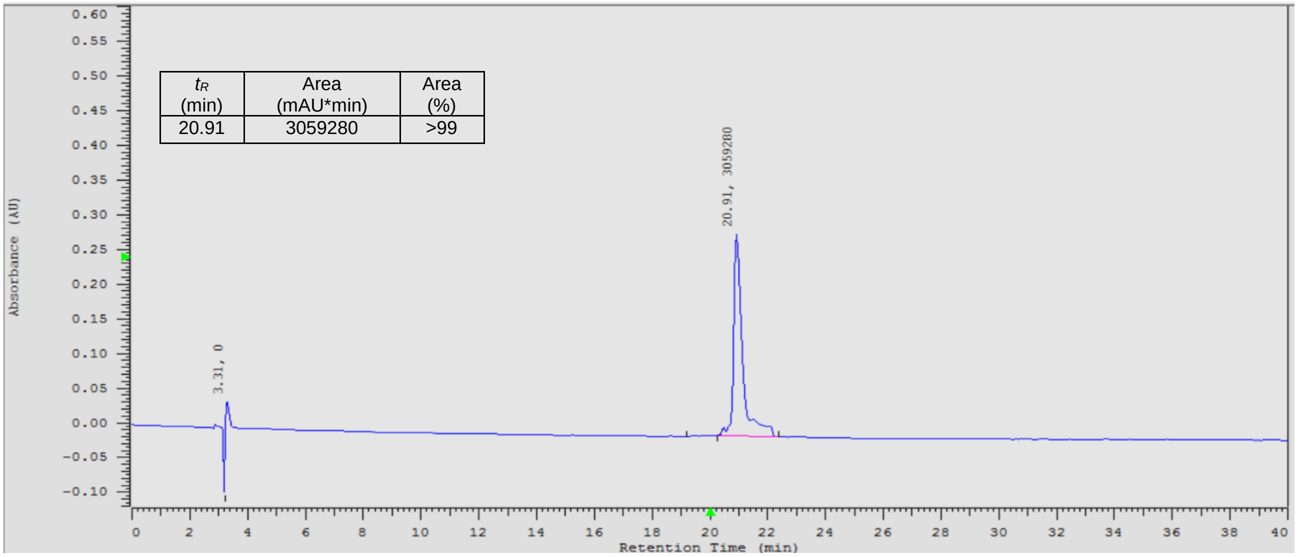

Peptide **2i**: RWVVR-RDYRFDMGFD-YWVWE (Ordered from Peptide2.0)
HPLC: Peptide **2i** retention time t_R = 23.07 min with a purity of 97%.

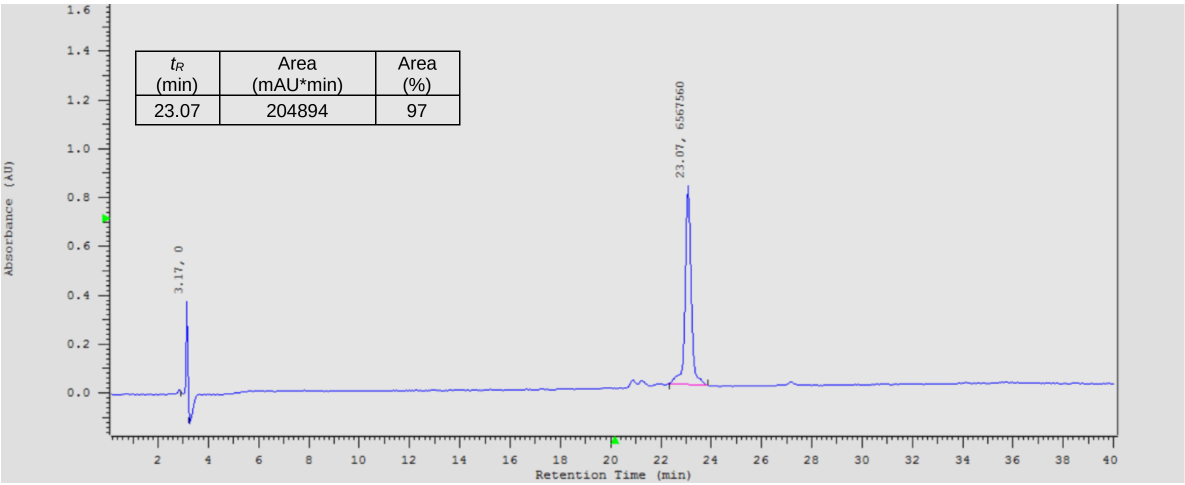

Peptide **2j**: RWVAR-RDYRGDMGFN-YWVWE (300 mg, 0.13 mmol of Wang resin was used)
HPLC: Peptide **2j** retention time t_R = 20.9 min with a purity of 99%.

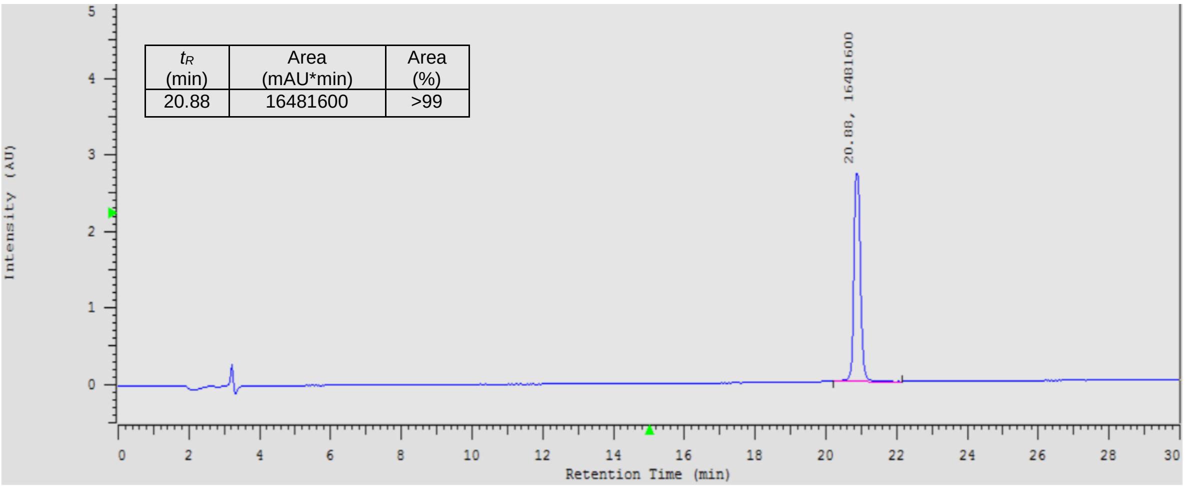

t_R (min)	Area (mAU*min)	Area (%)
20.88	16481600	>99

Peptide **2k**: RWVAR-RDYRFDMGFN-YWVWE (Ordered from Peptide2.0)
HPLC: Peptide **2k** retention time t_R = 22.59 min with a purity of 96%.

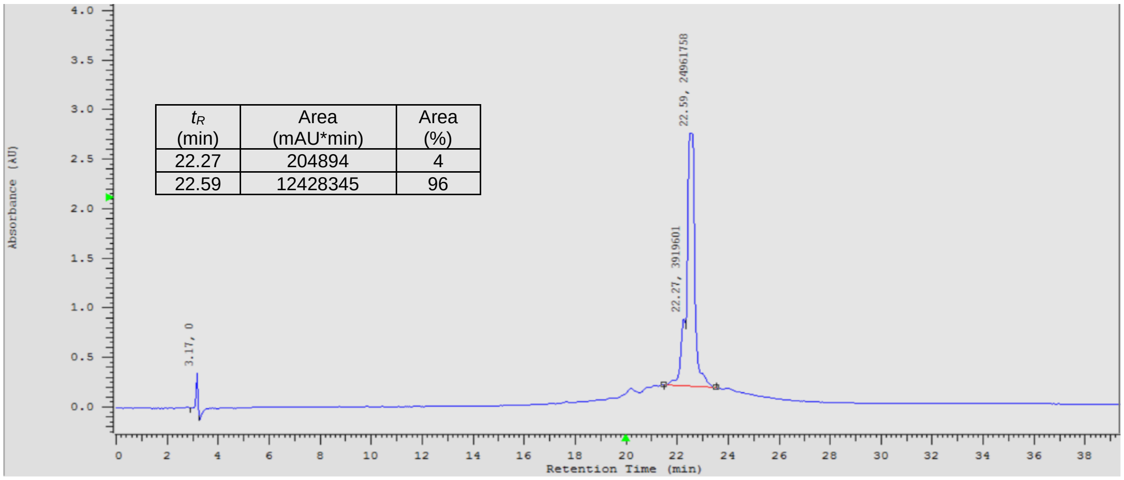

t_R (min)	Area (mAU*min)	Area (%)
22.27	204894	4
22.59	12428345	96

Peptide **2l**: cRWCAR-RDYRFDMGFD-YWCWE (300 mg, 0.13 mmol of Wang resin was used)
HPLC: Peptide **2l** retention time t_R = 21.84 min with a purity of 99%.

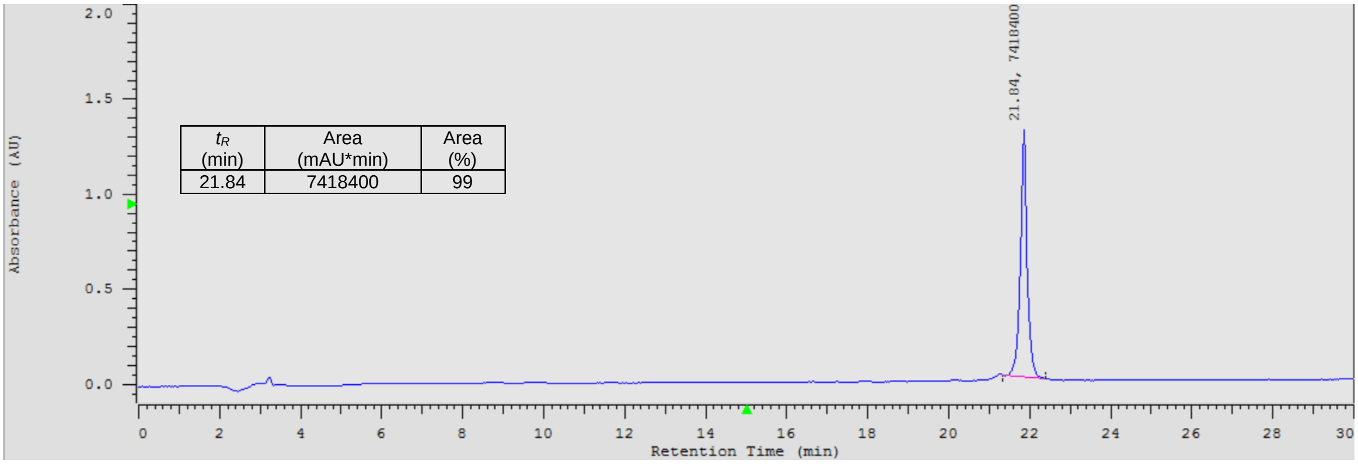

t_R (min)	Area (mAU*min)	Area (%)
21.84	7418400	99

Peptide **1e**: RWVW-SPDSSGVAY-WVWE (300 mg, 0.13 mmol of Wang resin was used)
HPLC: Peptide **1e** retention time t_R = 21.84 min with a purity of 99%.

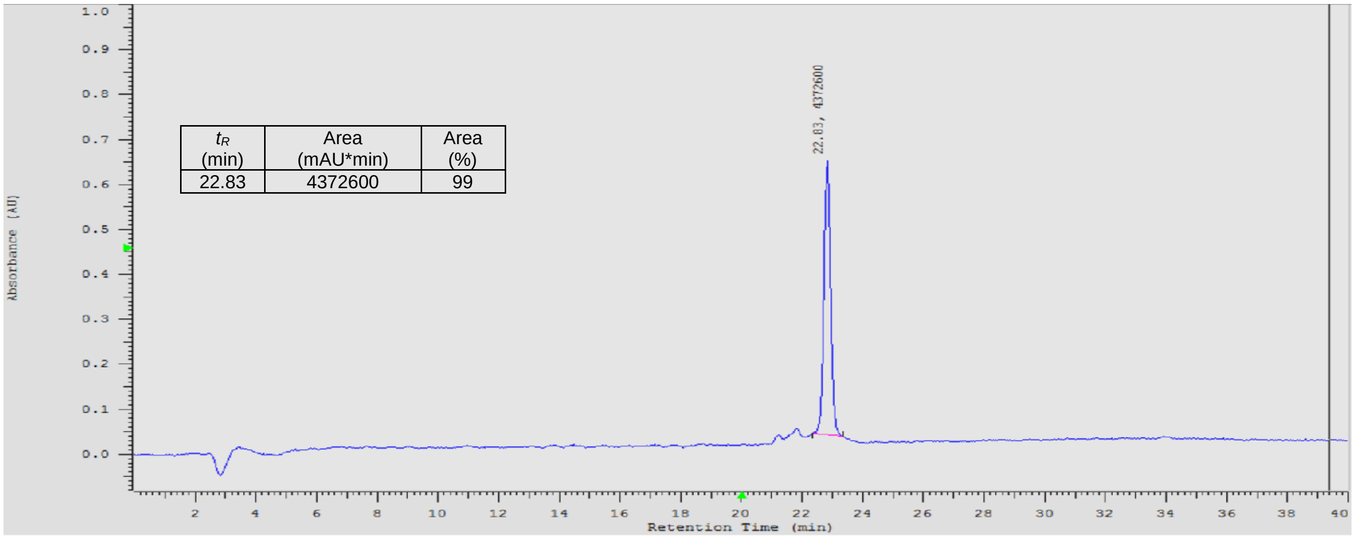

Peptide **1f**: RWVW-RDLDIATTRDY-WVWE (300 mg, 0.13 mmol of Wang resin was used)
HPLC: Peptide **1f** retention time t_R = 23.52 min with a purity of 93%.

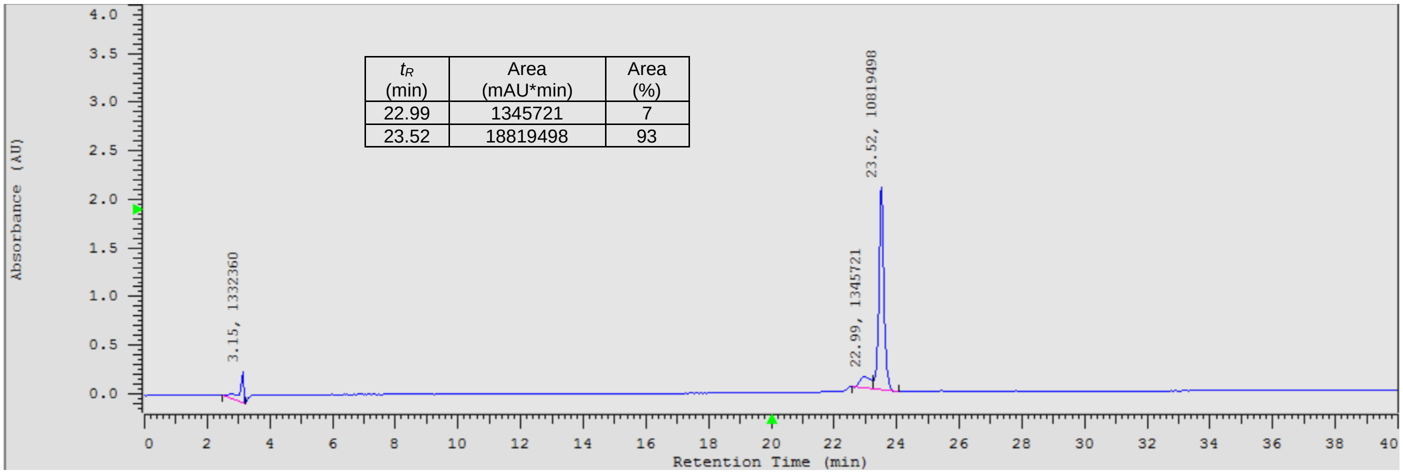

Peptide **2m**: RWVW-SPDSSGVAY-WVWE (300 mg, 0.13 mmol of Wang resin was used)
HPLC: Peptide 2m retention time t_R = 21.84 min with a purity of 99%.

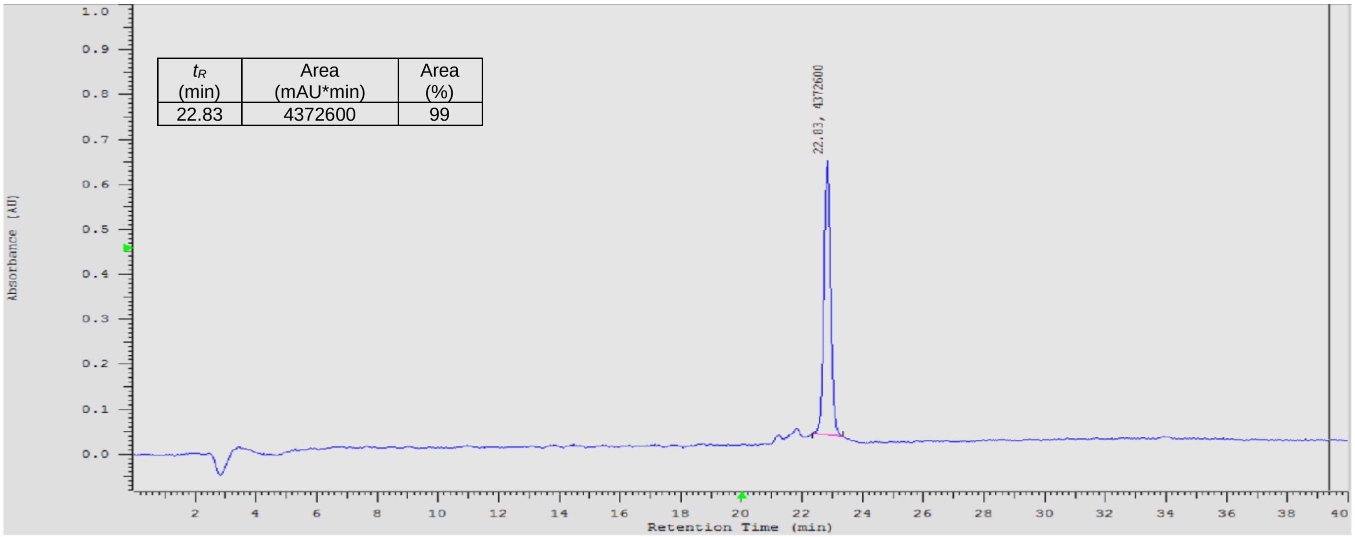

Peptide **2n**: RWVA-REGMNTDWYFDY-WHWE (300 mg, 0.13 mmol of Wang resin was used)
HPLC: Peptide **2n** retention time t_R = 23.33 min with a purity of 94%

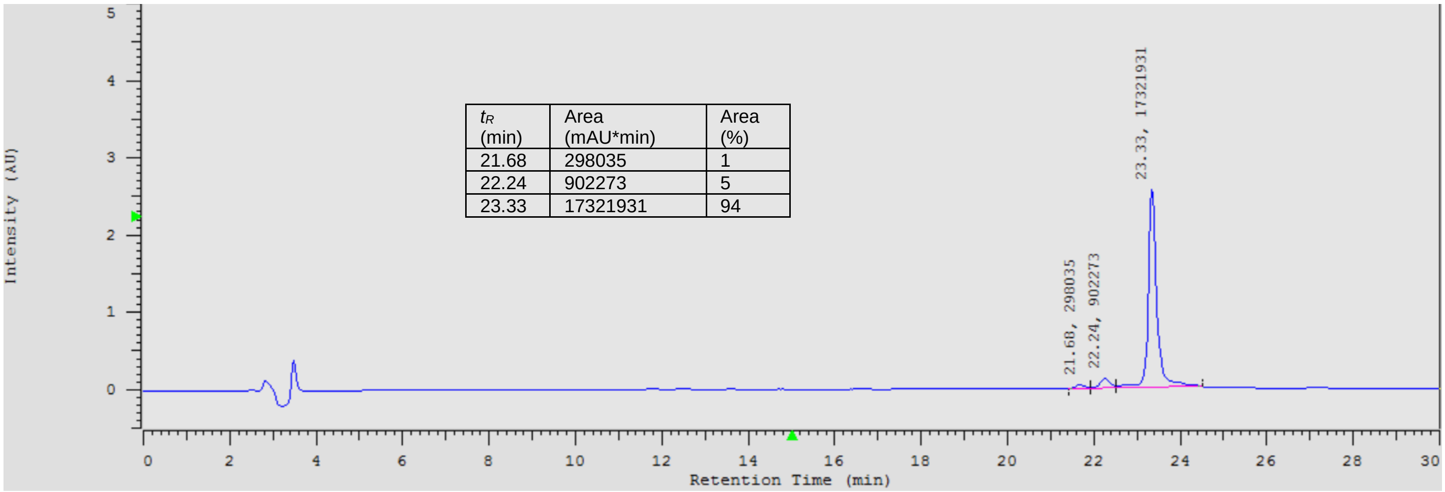

t_R (min)	Area (mAU*min)	Area (%)
21.68	298035	1
22.24	902273	5
23.33	17321931	94

Peptide **2o**: RWVA-REGMNTDGYFDY-WHWE (300 mg, 0.13 mmol of Wang resin was used)
HPLC: Peptide **2o** retention time t_R = 21.07 min with a purity of 98%

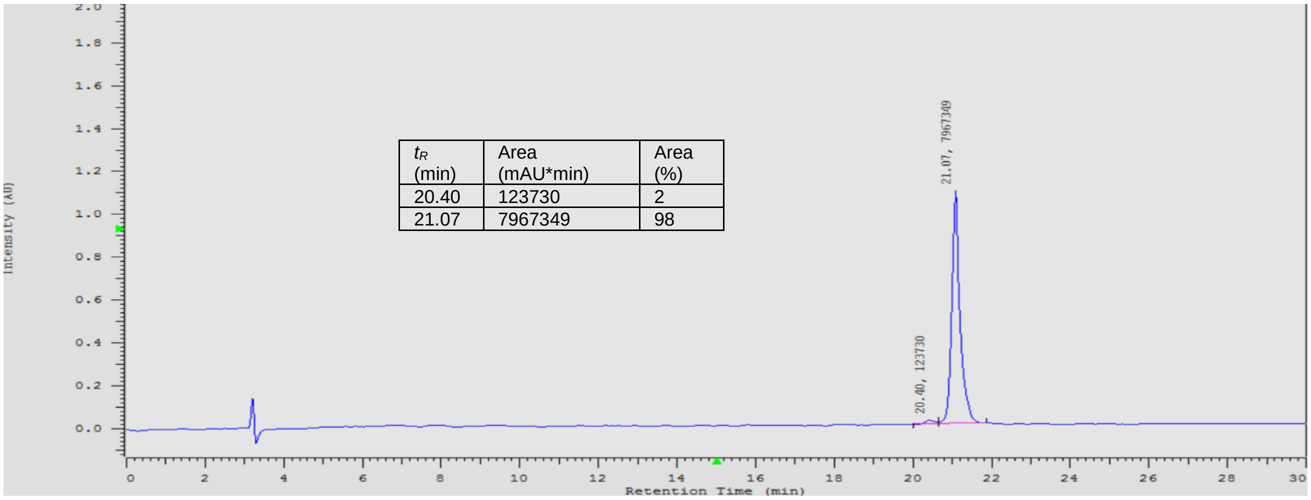

t_R (min)	Area (mAU*min)	Area (%)
20.40	123730	2
21.07	7967349	98

Peptide **2p**: RWVW-SPDSSGVAY-WVWE (300 mg, 0.13 mmol of Wang resin was used)
HPLC: Peptide 2p retention time t_R = 21.23 min with a purity of 95%.

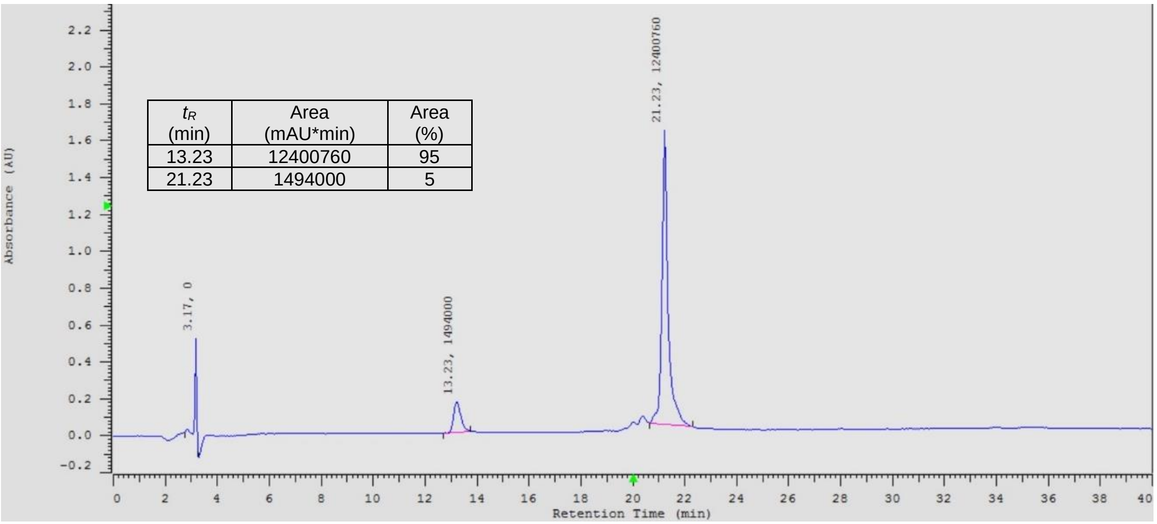

t_R (min)	Area (mAU*min)	Area (%)
13.23	12400760	95
21.23	1494000	5

Peptide **2q**: RWVA-RAYGNYWYIDV-WHWE (300 mg, 0.13 mmol of Wang resin was used)
HPLC: Peptide **2q** retention time t_R = 24.11 min with a purity of 99%

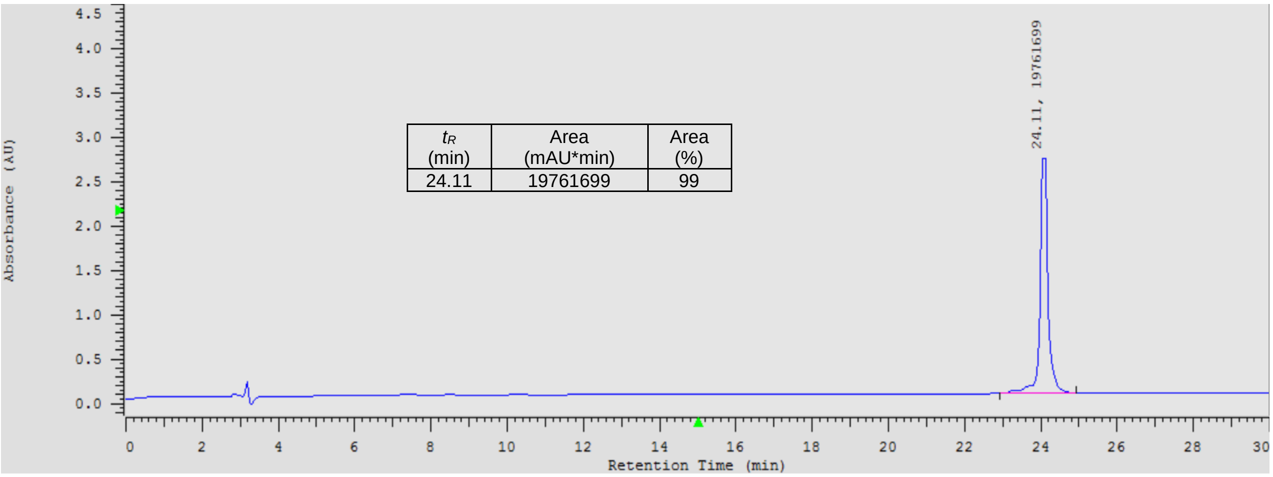

Peptide **2r**: RWVA-RAYGYNGYIDV-WHWE (300 mg, 0.13 mmol of Wang resin was used)
HPLC: Peptide **2r** retention time t_R = 21.89 min with a purity of 98%

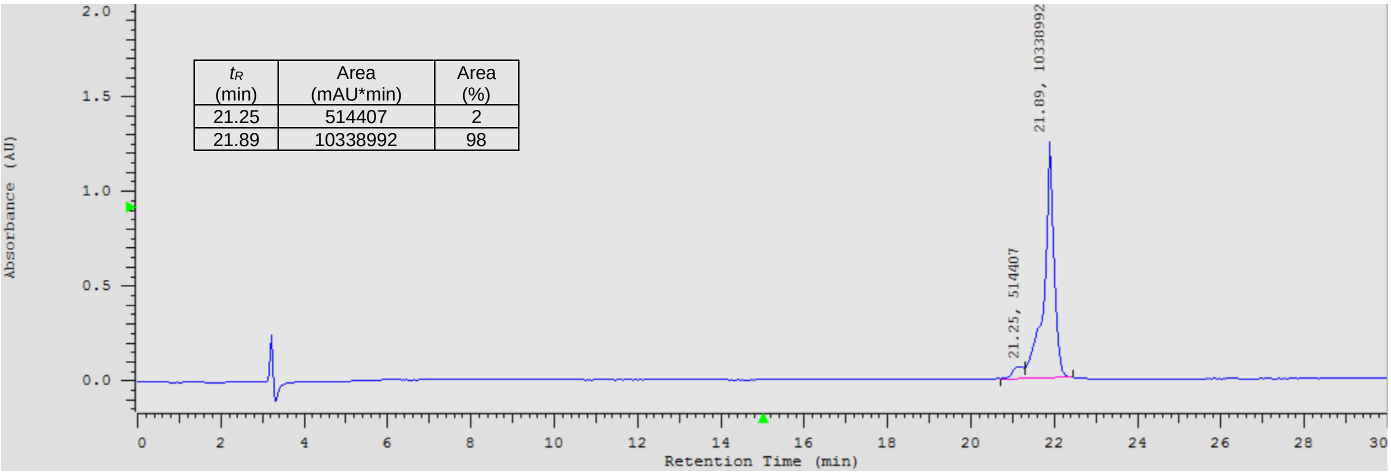

Peptide **2ca**: RWVAR-RDYRFD[Dab(ACA)]GFD-YWVWE (300 mg, 0.13 mmol of Wang resin was used)
HPLC: Peptide **2ca** retention time t_R = 20.00 min with a purity of 99%

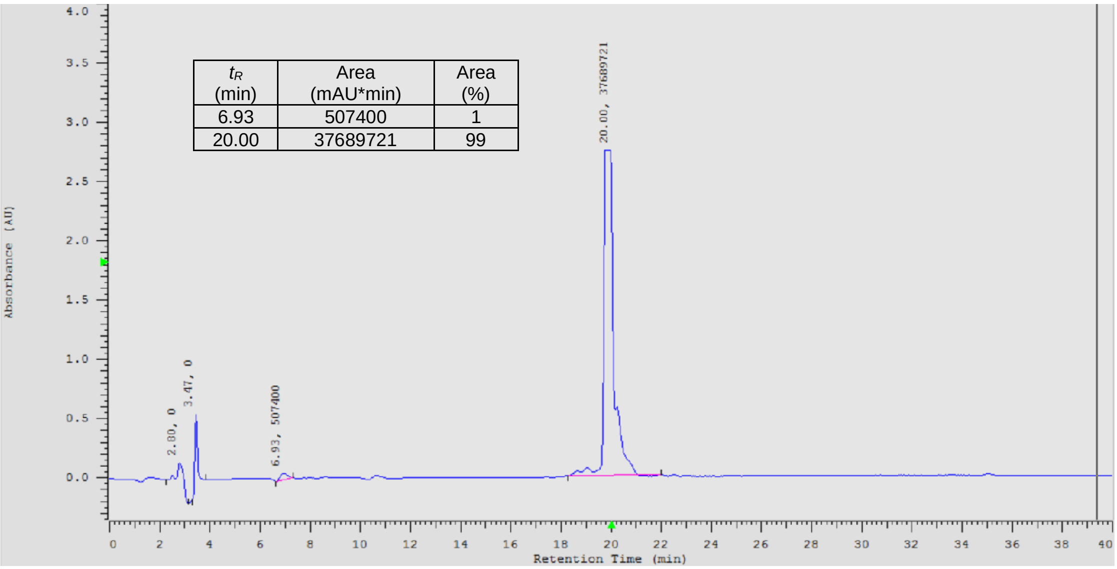

Peptide **2cb**: RWVAR-RDYRFD[Dap(ACA)]GFD-YWVWE (300 mg, 0.13 mmol of Wang resin was used)
HPLC: Peptide **2cb** retention time t_R = 22.53 min with a purity of 99%

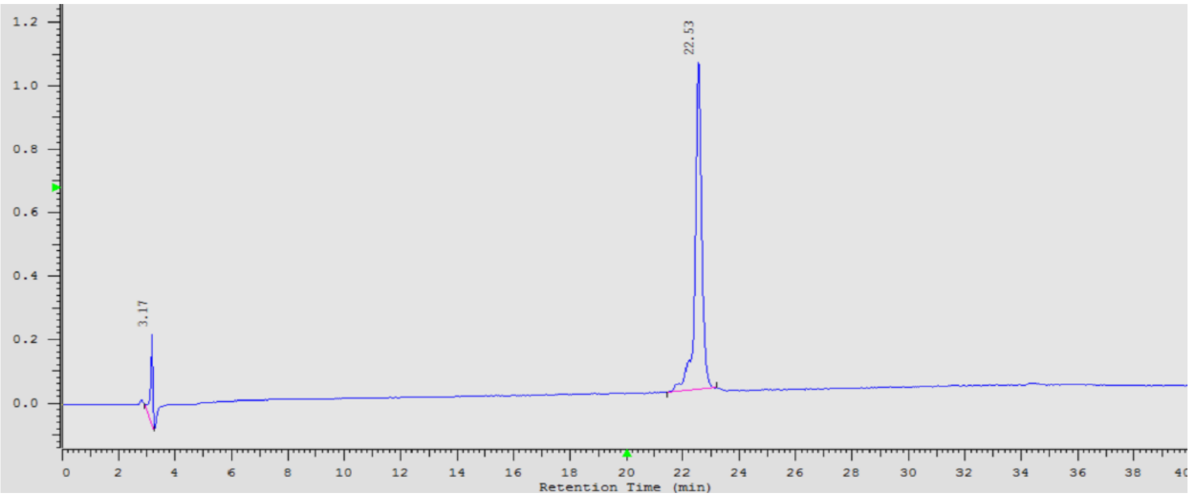

Peptide **2cc**: RWVAR-RDYRFD[Dab(DMA)]GFD-YWVWE (300 mg, 0.13 mmol of Wang resin was used)
HPLC: Peptide **2cc** retention time t_R = 20.56 min with a purity of 99%

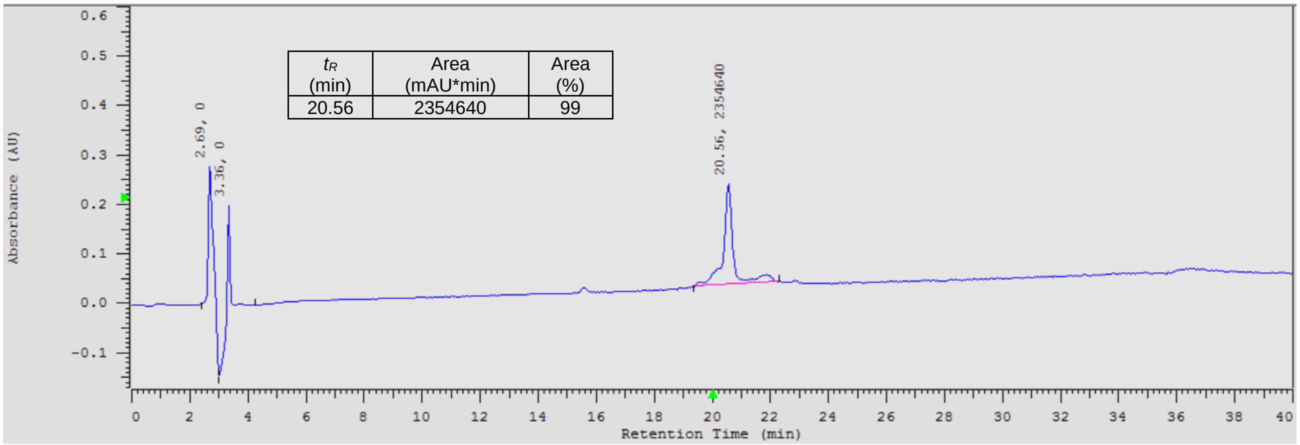

t_R (min)	Area (mAU*min)	Area (%)
20.56	2354640	99

Peptide **2cd**: RWVAR-RDYRFD[Dab(MAA)]GFD-YWVWE (300 mg, 0.13 mmol of Wang resin was used)
HPLC: Peptide **2cd** retention time t_R = 19.65 min with a purity of 99%

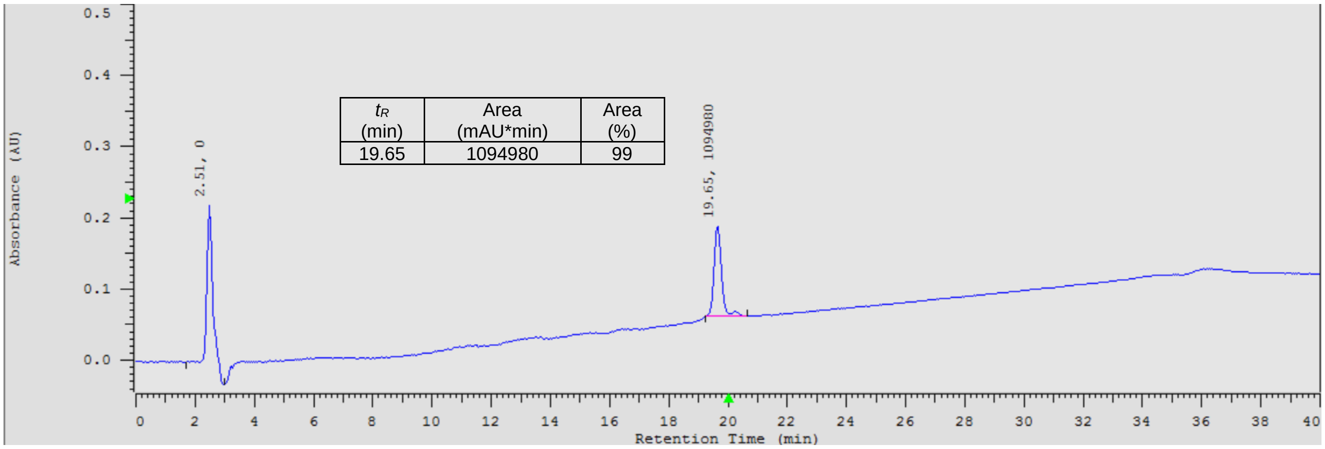

t_R (min)	Area (mAU*min)	Area (%)
19.65	1094980	99

Peptide **2pa**: RWVA-REGMNTDG[Dab(ACA)]FN-YWVWE (300 mg, 0.13 mmol of Wang resin was used)
HPLC: Peptide **2pa** retention time t_R = 20.72 min with a purity of 99%

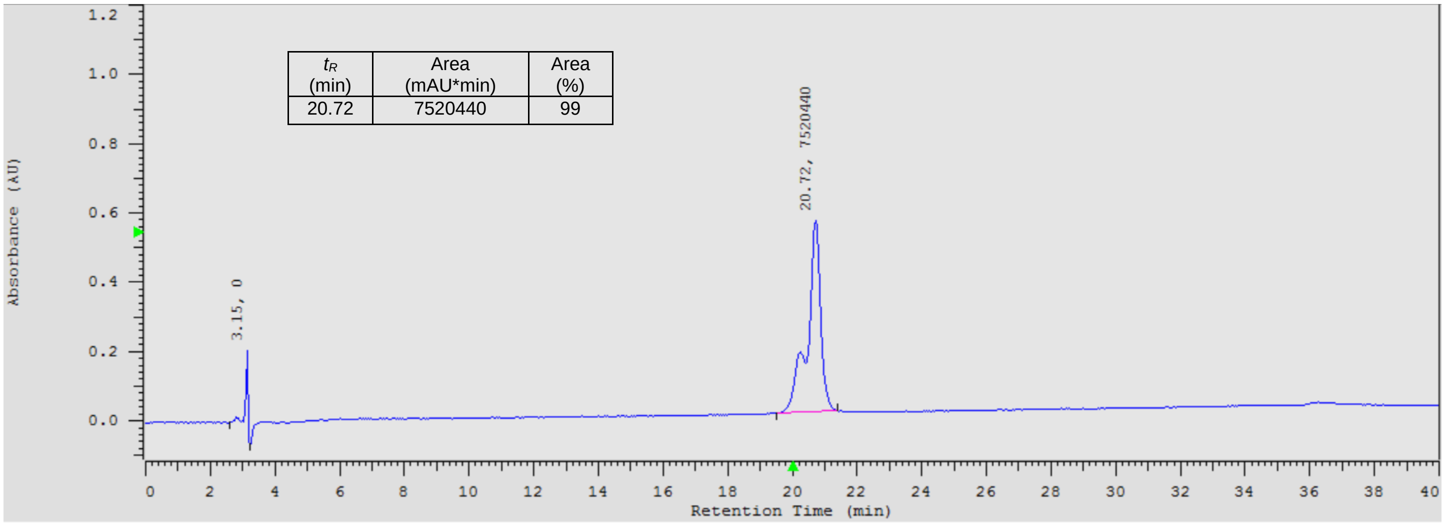

Peptide **2pb**: RWVA-REGMNTDG[Dap(ACA)]FN-YWVWE (300 mg, 0.13 mmol of Wang resin was used)
HPLC: Peptide **2pb** retention time t_R = 22.13 min with a purity of 99%

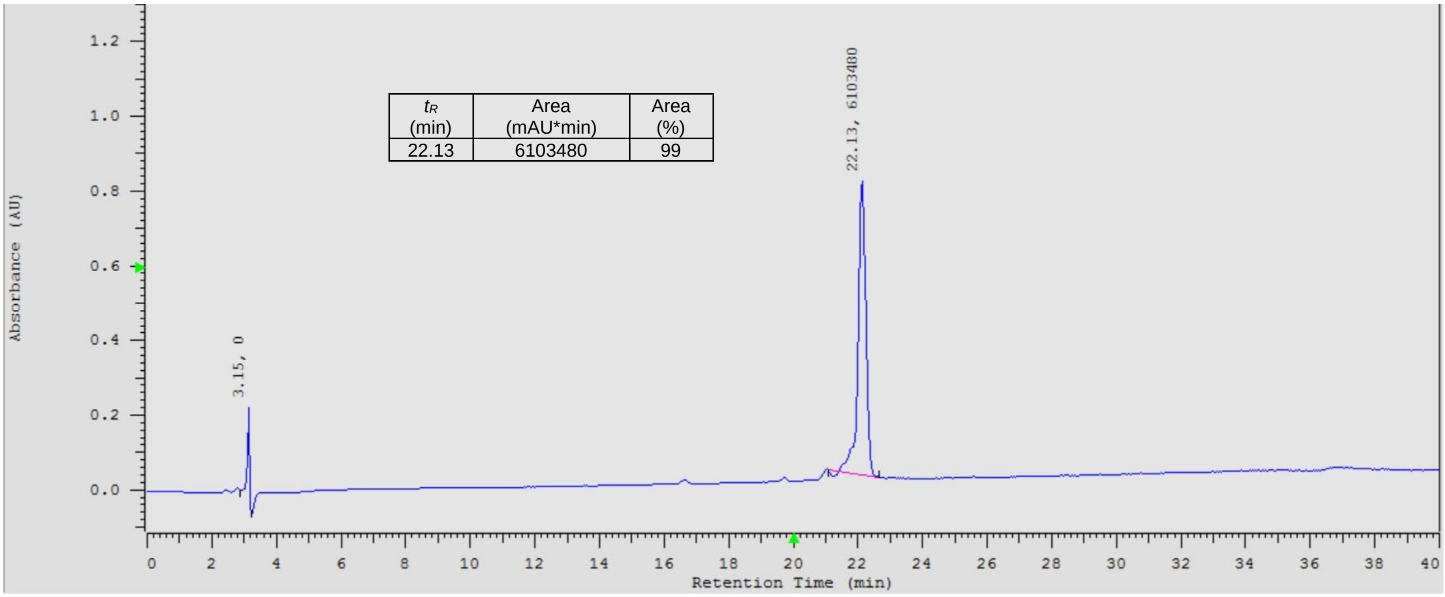

7. Comparison of β-hairpins Stability by Circular Dichroism (CD) Spectroscopy

CD Protocol. Peptides were solubilized in phosphate buffer (15mM, pH 6.5) with up to 20% methanol if required for solubility. The concentration in pure peptide was accurately determined by UV-absorbance measurements using a JASCO V-670 spectrophotometer using the combined molar absorptivity of Trp (ε_{280} = 5580 M^{-1} .cm^{-1} per Trp) and Tyr (ε_{280} = 1280 M^{-1} .cm^{-1} per Tyr) residues present in the sequences of *ADAPTins*. The samples were then subjected to recording of CD spectra using a JASCO J-810 Spectropolarimeter. The raw CD spectra were collected from 190 nm to 270 nm in units of mdeg every 0.1 nm. To baseline correct a blank sample was recorded to the solvent using the same parameters to subtract. Raw data from the Spectra Manager Software were treated using SpectraGryph 1.2, where the baseline was set to 0 between 250 nm to 260 nm and spectra were smoothed. Using the concentration, the smoothed and baseline corrected spectra were then converted from mdeg to molar ellipticity (deg.cm^2.$dmol^{-1}$). To collect raw data of peptide melting curves (unfolding hairpin/coil transition), the same spectropolarimeter was used varying the temperature from 0 to 95 °C at a ramp speed of 0.75 °C/min. Experimental melting curves were recorded at 228±2 nm (every 0.1 °C) depending on the maximum of the Trp/Trp interaction peak. Blank solutions were subjected to the same protocol to generate the baseline correction. Conversion of mdeg to molar ellipticity (deg• cm^2 •$dmol^{-1}$) and division by the number of tryptophan pairs contained within the peptide allowed for normalization of spectra between samples.

Individual Melting Curves Best-Fitting Protocol. Raw molar ellipticity data [θ(T)] at 228 nm were fitted to the Gibbs-Helmholtz equation for a two-state model (folded/unfolded transition) accordingly to the reported procedure by N. J. Greenfield[SI-6], and as used previously.[SI-7]

$$\theta(T) = \theta_U + (\theta_F - \theta_U)\frac{e^{-\frac{\Delta G(T)}{RT}}}{1+e^{-\frac{\Delta G(T)}{RT}}} \qquad \text{Eq. 1}$$

With

$$\Delta G(T) = \Delta H_m * \left(1 - \frac{T}{T_m}\right) - \Delta Cp * ((T_m - T) + T * \ln\left(\frac{T}{T_m}\right)) \qquad \text{Eq. 2}$$

And $[\theta(T)]_{230\pm2}$ representing the experimentally measured molar ellipticity at the temperature *T* which has been corrected by subtracting the experimental $[\theta]_{coil(95)}$ value obtained (at 95 °C) from a synthetic random coil:

$$\theta(T) = \theta_{measured}(T) - \theta_{coil(95)} \qquad \text{Eq. 3}$$

$[\theta]_U$ and $[\theta]_F$ are the respective molar ellipticity values for the 100%-unfolded and -folded states respectively. The random coil value was found in each type of solvent system used (ie. phosphate buffer, 10% MeOH and 20% MeOH). All random coil curves and calculated values at 95 °C can be found in the excel SI.

Table S5. Summary of peptides thermodynamic and hairpin folding parameters calculated in Origin 9.0.

Peptide	R^2 Value	ΔG_F^0 (kcal.mol^{-1})	ΔH_F^0 (kcal.mol^{-1})	$-T\Delta S_F^0$ (kcal.mol^{-1})	ΔCp_F^0 (cal.deg^{-1}.mol^{-1})	ΔH_m (kcal.mol^{-1})	T_m (ºC)	Hairpin %-fold 291K ---- 310K	
**RC**	Random Coil	—	—	—	—	—	—	—	—
**Native**	No fold	—	—	—	—	—	—	—	—
**1a**	0.9964	-1.13±0.005	-9.57±0.1	8.44±0.1	-322 ± 6	-18.2±0.09	51.5	90 ± 1%	76 ± 1%
**1b**	0.99938	-0.64±0.01	-8.94±0.02	8.29±0.02	-41 ± 1	-9.84±0.01	46.9	81 ± 1%	62 ± 1%
**1c**	0.99867	0.31±0.01	-3.35±0.01	3.66±0.01	-23 ± 0.4	-2.72±0.02	-2.99	40 ± 1%	32 ± 1%
**1d**	0.94985	0.010±0.001	1.09±0.04	-1.08±0.04	-189 ± 2	-0.037±0.07	34.9	48 ± 1%	49 ± 1%
**2a**	0.9928	-0.90±0.006	-10.8±0.1	9.87±0.1	-372 ± 10	-18.2±0.1	44.8	87 ± 1%	66 ± 1%
**2b**	0.99968	-1.24±0.01	-8.89±0.03	7.65±0.03	-193 ±1	-15.5±0.02	59.1	92 ± 1%	81 ± 1%
**2c**	0.99180	-1.26±0.01	-13.6±0.2	12.4±0.2	-29 ± 7	-14.5±0.08	54.1	93 ± 1%	77 ± 1%
**2d**	0.99825	-1.59±0.01	-19.8±0.2	18.2±0.2	0	-19.8±0.07	50.9	97 ± 1%	80 ± 1%
**2e**	0.98994	0.088±0.02	-2.31±0.03	2.40±0.02	-107±5	-0.070±0.6	3.96	48 ± 1%	42 ± 1%
**2f**	0.99818	-1.33±0.01	-7.09±0.07	5.76±0.07	-208 ±2	-15.5±0.04	65.4	92 ± 1%	85 ± 1%
**2g**	0.99609	-0.16±0.01	5.27±0.05	-5.11±0.05	-287 ± 3	-0.136±0.2	43.7	37 ± 1%	49 ± 1%
**2h**	0.99730	-1.12±0.01	-5.88±0.05	4.77±0.05	-145 ± 2	-12.1±0.04	67.8	89 ± 1%	81 ± 1%
**2i**	0.97567	0.094±0.05	-3.38±0.05	3.48±0.01	-211 ± 48	-0.087±0.2	9.25	49 ± 1%	39 ± 1%
**2j**	0.99818	-1.63±0.01	-15.9±0.02	14.3±0.09	-29 ± 3	-16.8±0.03	57.8	97 ± 1%	84 ± 1%
**2k**	0.98075	-0.01±0.003	-0.54±0.04	0.53±0.04	-252 ± 3	-1.20±0.2	27.5	50 ± 1%	47 ± 1%
**2l**	0.99293	-0.77±0.01	-5.46±0.05	4.69±0.05	-44 ± 2	-7.29±0.04	66.0	82 ± 1%	71 ± 1%
**1e**	0.98969	-0.90±0.01	-6.50±0.06	5.60±0.06	-148 ± 4	-11.5±0.05	58.3	85 ± 1%	74 ± 1%
**1f**	0.99751	-0.49±0.01	-10.5±0.06	9.96±0.06	-15 ± 3	-10.7±0.04	39.4	78 ± 1%	53 ± 1%
**2m**	β-sheet	—	—	—	—	—	—	—	—
**2n**	0.79513	0.46±0.01	-5.65±0.18	6.11±0.19	-97 ± 14	-2.75±0.80	-5.1	36 ± 2%	23 ± 2%
**2o**	0.99751	0.47±0.01	-5.97±0.01	6.45±0.02	-2 ± 1	-5.93±0.03	2.8	36 ± 1%	23 ± 1%
**2p**	0.99965	-0.99±0.009	-11.9±0.1	10.9±0.1	-115 ± 4	-14.7±0.06	48.9	89 ± 1%	70 ± 1%
**2q**	β-sheet	—	—	—	—	—	—	—	—
**2r**	0.94547	-1.15±0.01	-8.29±0.3	7.13±0.3	-44 ± 9	-10.2±0.2	67.6	91 ± 1%	80 ± 1%
**2ca**	0.99919	-0.79±0.002	-11.2±0.04	10.5±0.04	-23 ± 2	-11.8±0.02	46.8	86 ± 1%	64 ± 1%
**2cb**	0.99276	-0.14±0.002	-3.09±0.03	2.95±0.03	-204 ± 2	-5.21±0.03	35.3	58 ± 1%	49 ± 1%
**2cc**	0.99913	-0.93±0.002	-9.43±0.04	8.50±0.04	-268 ± 2	-15.8±0.04	48.7	87 ± 1%	70 ± 1%
**2cd**	0.98941	-0.12±0.003	-3.78±0.03	3.89±0.03	-212 ± 4	0.07±0.07	6.7	48 ± 1%	37 ± 1%
**2pa**	0.99961	-0.48±0.008	-7.36±0.01	6.87±0.01	-253 ± 1	-11.4±0.02	41.0	74 ± 1%	56 ± 1%
**2pb**	0.99914	-0.30±0.001	-7.50±0.02	7.20±0.02	-261 ± 1	-10.2±0.02	35.3	68 ± 1%	48 ± 1%

Global Fit Protocol

Melting curves were directly compared by fitting to a common maximum shared [θ] value between all peptides with common sequences. The global fitting was generated in OriginPro 9.0. At the end of the first fitting iteration the highest maximum molar ellipticity [θ_F] value (100%-folding) was obtained and shared between all other peptides. The initial best-fit was then processed again for as many iterations as needed to allow each curve in the model to be fitted to the same maximum and converge. All data for individual and global fitting are reported for a direct comparison (**Tables S6-S7**).

Individual fitting involves analyzing each melting curve separately, fitting a specific mathematical model (two-state approximation) to each curve to obtain the melting temperature (T_m) and thermodynamic parameters. Global fitting involves simultaneously fitting multiple melting curves acquired under different conditions (*e.g.*, different concentrations or sequences) to the same mathematical model. By fitting these curves simultaneously, a global fitting ensures consistency in the analysis, reducing the risk of overfitting introduced by individual biased [θ_F] maxima. Yet, a global fitting assumes that the same model and parameters apply to CD-melts, which may not always be the case, leading to potential inaccuracies. In this study, the global fitting protocol seems successful as long as experimental conditions are relatively consistent and peptide sequences are homologous. Overall, the choice of global fitting for the GY-14 peptide series (**Figure S18**) was successful because the non-covalent ADAPTins (**2n-p**) and the covalent analogs **2pa,pb** were all folded in a very similar manner (similar molar ellipticity intensities). In the case of pembrolizumab mimics (**Figure S19**), the global fitting protocol of melting curves was deemed suitable if outlier **2ca** was excluded (*i.e.* R^2 value of 0.989 to 0.997).

*GY-14 CDR-H3 Analogs of **2p***

Table S6. Comparison of folding and melting temperatures using individual and global fitting protocols for **GY-14** peptides (covalent and non-covalent *ADAPTins*).

Peptide ID	Peptide sequence[a]	Conc. (μM)	228 nm				202 nm			
			T_m (°C)		χ_F (291 K)		T_m (°C)		χ_F (291 K)	
			Indiv.	Glob.[b]	Indiv.	Glob.[b]	Indiv.	Glob.[b]	Indiv.	Glob.[b]
**2n**	RWVA-REGMNTD**W**YFDY-WHWE	42	-5.1	-	36%	0%	-	-	-	-
**2o**	RWVA-REGMNTDGYFDY-WHWE	39	2.8	14.6	36%	47%	7.9	-18.3	38%	19%
**2p**	RWVA-REGMNTDGYFNY-WVWE	23	48.9	48.6	86%	88%	44.6	45.7	85%	88%
**2pa**	RWVA-REGMNTDG[Dab(ACA)]FNY-WVWE	29	41.0	44.4	74%	82%	41.8	42.3	82%	82%
**2pb**	RWVA-REGMNTDG[Dap(ACA)]FNY-WVWE	25	35.3	40.3	68%	79%	39.3	-14.3	79%	38%

[a] Residues highlighted in color designate substitutions. [b] Global fit data comes from sharing the maximum molar ellipticity value between all curves in the data set, where the individual fit maximizes the values for each individual curve.

*Pembrolizumab CDR-H3 Analogs of **2c***

Table S7. Comparison of folding and melting temperatures using individual and global fitting protocols for pembrolizumab peptides (covalent and non-covalent *ADAPTins*).

Peptide ID	Peptide sequence[a]	Conc. (μM)	228nm				202nm			
			T_m (°C)		χ_F (291 K)		T_m (°C)		χ_F (291 K)	
			Indiv.	Glob.[b]	Indiv.	Glob.[b]	Indiv.	Glob.[b]	Indiv.	Glob.[b]
**2c**	RWVAR-RDYRFDMGFD-YWVWE	36	36.7	48.0	56%	63%	62.2	67.5	73%	97%
**2ca**	RWVAR-RDYRFD[Dab(ACA)]GFD-YWVWE	15	43.7	-	84%	-	29.0	41.4	58%	76%
**2cb**	RWVAR-RDYRFD[Dap(ACA)]GFD-YWVWE	23	52.4	13.6	87%	50%	27.6	27.9	57%	57%
**2cc**	RWVAR-RDYRFD[Dab(DMA)]GFD-YWVWE	21	49.8	51.7	88%	95%	44.3	9.7	83%	47%
**2cd**	RWVAR-RDYRFD[Dab(MAA)]GFD-YWVWE	29	8.1	-	48%	27%	-	-	43%	19%

[a] Residues highlighted in color designate substitutions. [b] Global fit data comes from sharing the maximum molar ellipticity value between all curves in the data set, where the individual fit maximizes the values for each individual curve.

Figure S18. CD Analysis of **GY-14** peptides. (**A**) Spectra taken every 10 degrees. (**B-C**) Melting curves obtained from a global fit protocol for the bands at 228 and 202 ± 2 nm (data points every 0.1 ºC).

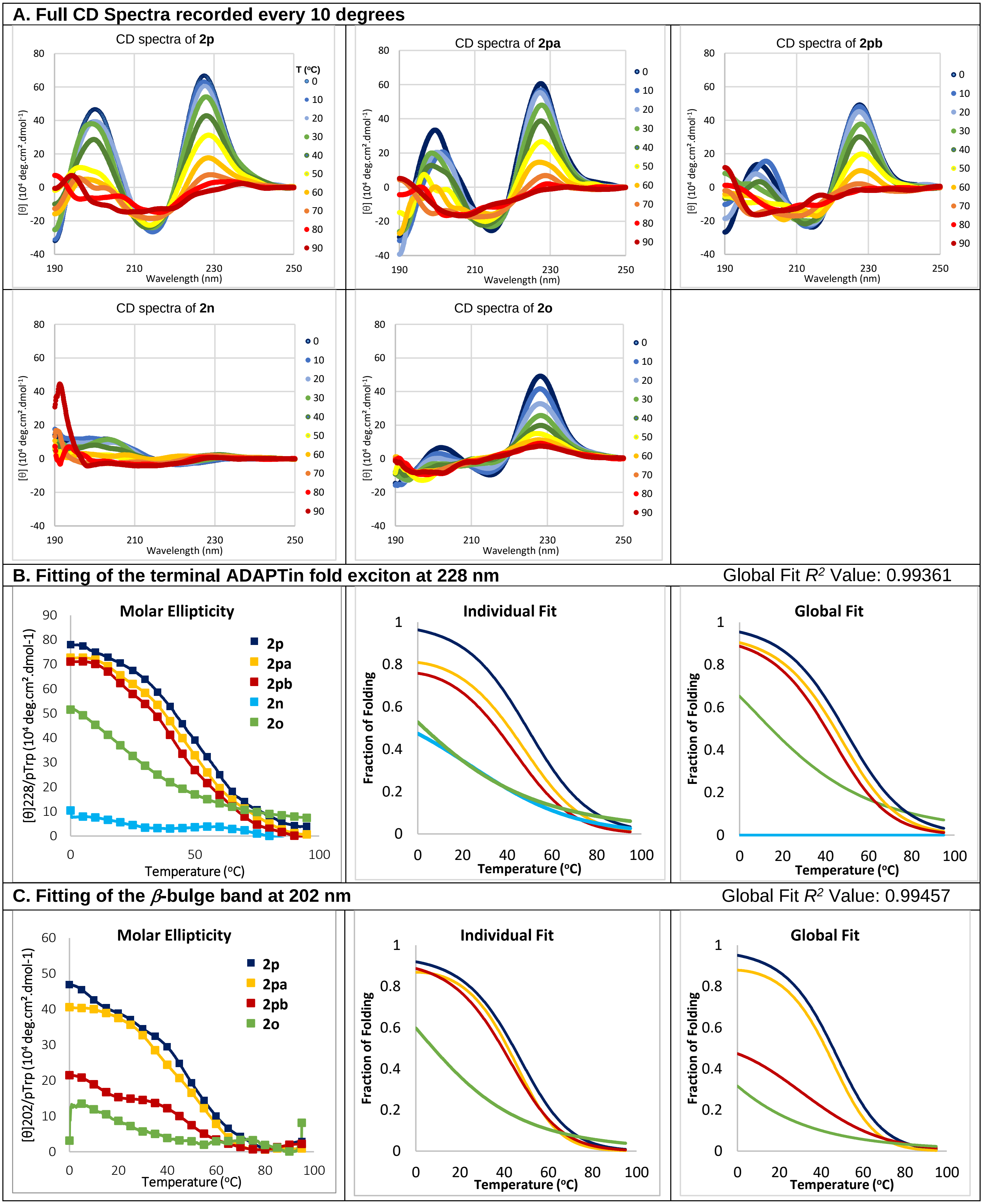

Figure S19. CD Analysis of pembrolizumab peptides. (**A**) Spectra taken every 10 degrees. (**B-C**) Melting curves obtained from a global fit protocol for the bands at 228 and 202 ± 2 nm (data points every 0.1 ºC).

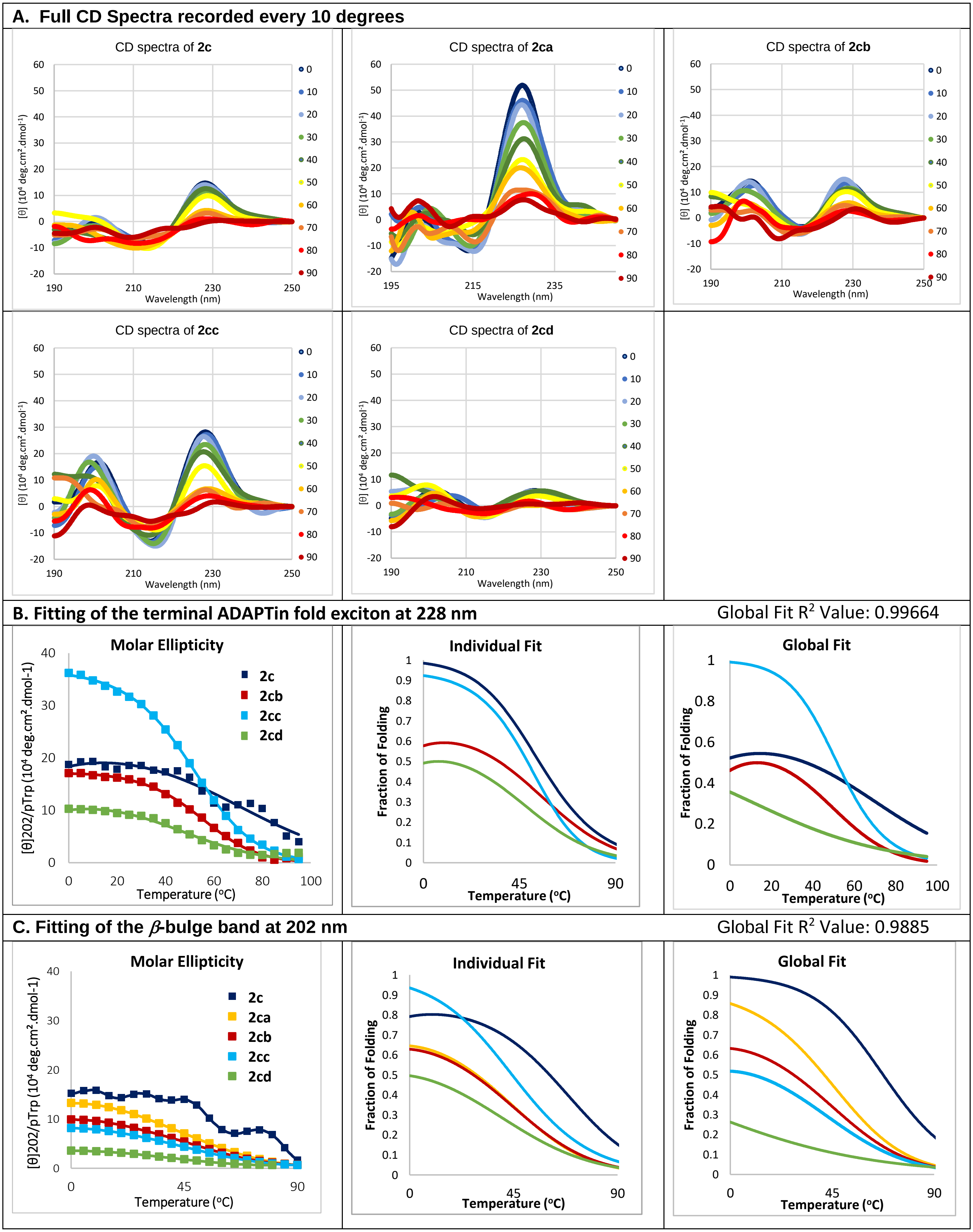

8. Immunoblocking ELISA Assay

Peptide inhibitor stock solutions were prepared in the concentration range of approximately 1-5 mM in DMSO. Screening and profiling assays for PD1/PDL1 interaction inhibitors were conducted using assay kits from BPS Bioscience (cat # 72005). Initially, a 96-well plate was coated with PDL1 at 2.0 μg/mL overnight at 4 °C. The plate was then washed and blocked with blocking buffer for 1 h at room temperature. Inhibitor solutions (ranging from 0.01 to 100 μM) and a blank solution (DMSO/PBS buffer) were added to designated wells. To initiate the reaction, 0.5 ng/μL of biotin-labeled human PD-1 was added to positive control wells and wells containing inhibitor solutions. After a 2 h incubation at room temperature, the plate was washed and blocked again for 10 min. Streptavidin-HRP (diluted at 1 : 1000) was added and incubated for 1 h at room temperature with gentle shaking. The plate was washed and blocked once more for 10 min. ELISA ECL substrate was added, and the resulting chemoluminescence was immediately measured in a luminometer (FLUOstar OPTIMA, BMG Labtech). The luminescence value of the blank was subtracted from all readings according to the manufacturer's instructions. The half maximal effective concentrations (EC_{50}) values were determined by fitting the experimental normalized luminescence data to the Hill model, with RLUmax values representing maximal relative luminescence values from the PD1/PDL1 interaction. Mean EC_{50} values and their corresponding standard deviations were calculated from three independent experiments (**Table S8**). Ligand efficiency (LE) values were calculated using the formula LE= $-RT\textit{ln}(EC_{50})/HA$, where HA is the number of non-hydrogen atoms in each ligand, omitting the similar sequence (strap) between each peptide (RWV•••VWE).

Table S8. Tabulated mean EC_{50} values their corresponding standard deviations (n = 3) and the calculated ligand efficiency values (LE).

**Peptide**	**EC_{50} Value** (nM)	**SD** (nM)	**LE**
**1a**	3290	--	--
**1b**	7080	1620	0.06
**1c**	12270	1500	0.06
**1d**	6799	55	0.05
**2b**	7055	234	0.06
**2c**	249	65	0.07
**2d**	5651	263	0.06
**2e**	586.3	144	0.06
**2f**	862.3	34	0.06
**2g**	78	12	0.07
**2h**	780.6	26	0.06
**2i**	70.0	5	0.07
**2j**	97	5	0.07
**2k**	170	12	0.07
**2l**	584	93	0.06
**1e**	267	40	0.10
**1f**	3359	560	0.06
**2m**	144	29	0.07
**2n**	252	150	0.07
**2o**	349	60	0.07
**2p**	160	80	0.08
**2q**	230	10	0.07
**2r**	170	5	0.08
**2ca**	5220	150	0.12
**2cb**	3979	400	0.13
**2cc**	1530	250	0.13
**2cd**	9870	1060	0.11
**2pa**	950	80	0.16
**2pb**	3040	270	0.15

9. Competitive Binding Kinetics

Assay kits (cat# 72016) were obtained from BPS Bioscience. In brief, 96-well plates were coated with PD1 at 2.0 μg/mL overnight at 4 °C. The plates were washed and blocked with blocking buffer for 1 h at room temperature. Non-Covalent peptide competitor **2c** (3.0 and 0.75 μM), covalent peptides **2ca** and **2cb** (3.0 μM) and a blank solution were added to designated wells and incubated at room temperature for 60 min. To initiate the reaction, biotinylated PDL1 in an excess amount of 30 μg/mL was added every 10 minutes for 2 h. Plates were washed and blocked again for 10 min. Streptavidin-HRP (1 : 1000 dilution) was added and incubated for 1 h at room temperature with slow shaking. Plates were washed and blocked again for 10 minutes. A colorimetric streptavidin-HRP substrate was then added until a blue color developed in the positive control well, followed by the addition of 100 μl of an HCl solution (1.0 M) to each well. The absorbance was measured at 450 nm using a Biorad xMark™ microplate absorbance spectrophotometer. Abs values consistent with the PD1•PDL1 complex formation concentrations were plotted as a function of time at the varying concentrations of peptide blocker **2c** and covalent analogs **2ca** and **2cb**.

A UV-Vis assay was used to assess the competitive binding of the non-covalent *ADAPTin*s **2c** or **2k** to PD1 (pre-coated plates) in presence of a large excess of PDL1 (ligand 12 equiv., 560 μM). PD1 coated plates (n = 3) were incubated with non-covalent peptides **2k** and **2c** at varying concentrations or with covalent analogs **2ca**/**2cb** (**Figure S20**) for 1 h before the addition of PDL1. In this competitive PDL1 inhibition assay, model kinetic curves were obtained following a procedure reported by S. R. J. Horae.[SI-8] The kinetic values of k_{on} and k_{off} were derived from the fits to determine the inhibition constant K_i.

Figure S20. A) Kinetic PDL1 binding curves at varying concentrations of **2k** to determine K_i value. **B)** Kinetic PDL1 binding curves at varying concentrations of **2c** (non-covalent) to determine K_i value and with **2ca-cb** (covalent).

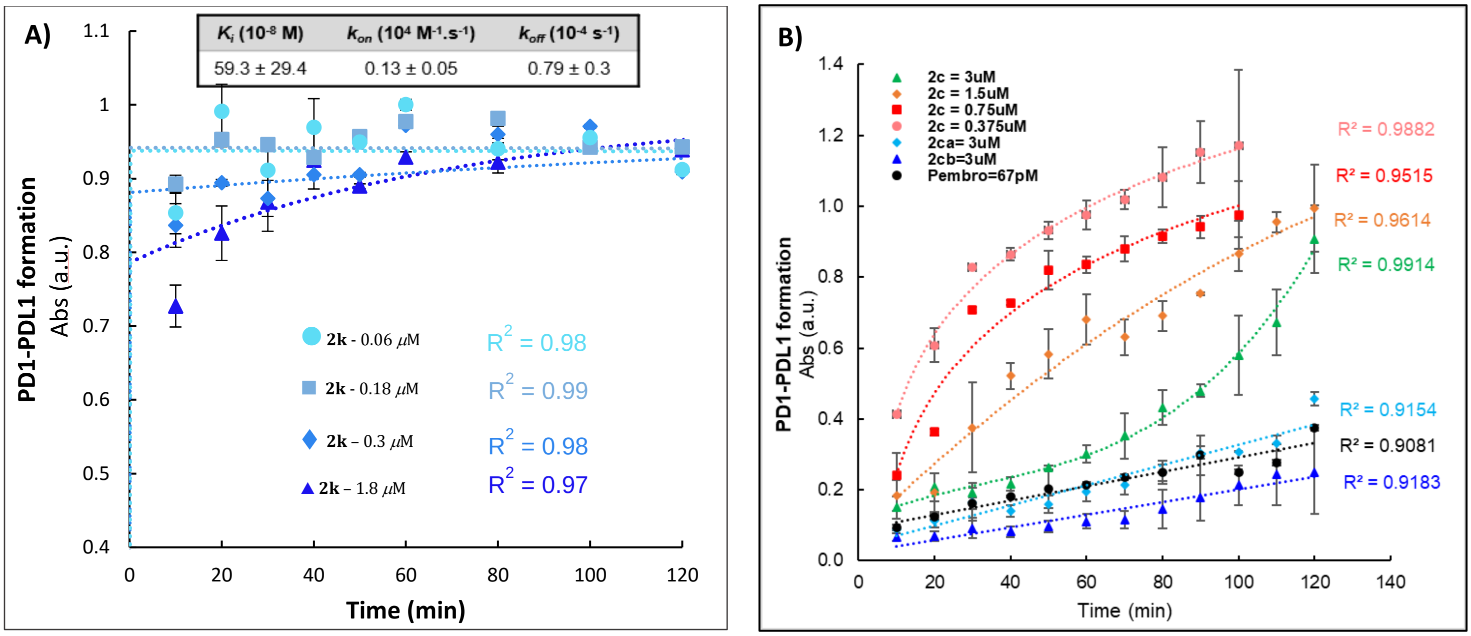

10. SDS-PAGE and Mass Spectrometry Experiments for the Covalent Binding of ADAPTins to PD1

SDS-PAGE Experiments.

Recombinant human PD1 His-tagged protein (100 µg purchased from R&D Systems™) was reconstituted in 400 µL of phosphate buffer (15 mM, pH 6.5). To remove all the complex N-linked sugars, 5 µL of the PD-1 solution was added to a mixture of PNGase F enzyme (4.5 µL Gibco™) following manufacturer's instructions and incubation for 1 h at 50 °C. Electrophilic *ADAPTin* peptide **2cb** was solubilized in phosphate buffer (15 mM, pH 6.5) to a stock concentration of 72 µM. Peptide **2cb** (1, 2, 4, 8, and 16 eq.) was then added to the mixture of PD-1 and PNGase F enzyme to be incubated overnight at 37 °C. Final concentration of PD-1 was 2.2 µM in each case. Samples were then heated at 70° C with NuPAGE® reducing agent and LDS buffer for 10 mins (denaturation step) was completed before loading onto gel. One-dimensional SDS-PAGE conditions using 10% Bis-Tris NuPAGE® gels were optimized with a MOPS running buffer for 1 h at 200 V using a XCell SureLock® mini-cell system (ThermoFisher Scientific™). Once the electrophoresis migrations achieved, gels were stained with SimplyBlue Safe Stain (Coomassie stain) and imaged (**Figure S21**). Similar experimental conditions were carried out to evaluate the covalent binding of our series of electrophilic peptides **2ca-cd** (**Figure S22**).

Trypsin Digestion Standard Protocol.

Bands of interest were excised from the SDS-PAGE using a razor blade. Pierce™ in-gel Tryptic Digestion Kit (cat #. 89871, ThermoFisher™) was used to achieve all in-gel trypsin digestions, following manufacturers' instructions. Briefly, gel slices were destained, reduced, alkylated using iodoacetamide, shrunk with acetonitrile, and then extracted and digested with the trypsin mixture. The resulting solution was pipetted off the gel slice and used for high-resolution mass spectrometry (HR-LCMS).

Overall, the SDS-PAGE gels analyzed (**Figures S21/22**) suggest the presence of a more intense band of higher molecular weight within the PD-1 band itself. Yet, our results did not show a clear enough band of covalent binding because our electrophilic peptides are about 2.5 kDa in molecular weight while the smear of the *N*-deglycosylated forms of PD-1 is larger than 5 kDa. Although the darker intensity of the bands with increasing equivalence in electrophilic peptides **2ca** and **2cd** was observed, the expected fragmentation patterns for the covalent bonding could not be observed by HR-LCMS analysis of the treated and digested bands. For the positive control, several fragments unbound PD-1 were verified using HR-LCMS, yet none of the treated bands after incubation with our electrophilic peptides showed any differences in the digested band results.

Figure S21. Full SDS-Page with varying equivalence of peptide (**2cb**) to PD-1. Lanes 1 and 10 are SeeBlue™ Plus2 Pre-stained protein ladder, lanes 2, 6, and 9 negative control PD-1 treated with PNGaseF enzyme with no peptide added, lanes 3, 4, 5, 7, and 8 are **2cb** (1, 2, 4, 8 and 16 eq., respectively) incubated with PNGaseF treated PD-1 overnight at 37 °C.

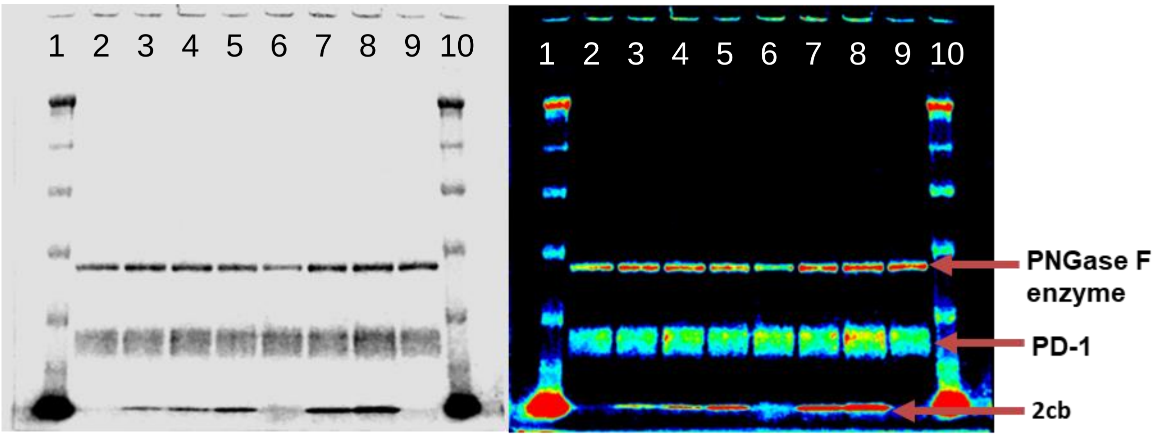

Figure S22. Full SDS-Page with varying equivalence of peptides (**2ca**, **2cb**, **2cc**, and **2cd**) to PD-1 (2 µg/well). Positive control 4eq **2cb** incubated overnight with PNGase F treated PD-1 and lyophilized. Negative Control of PNGase F treated PD-1 incubated overnight.

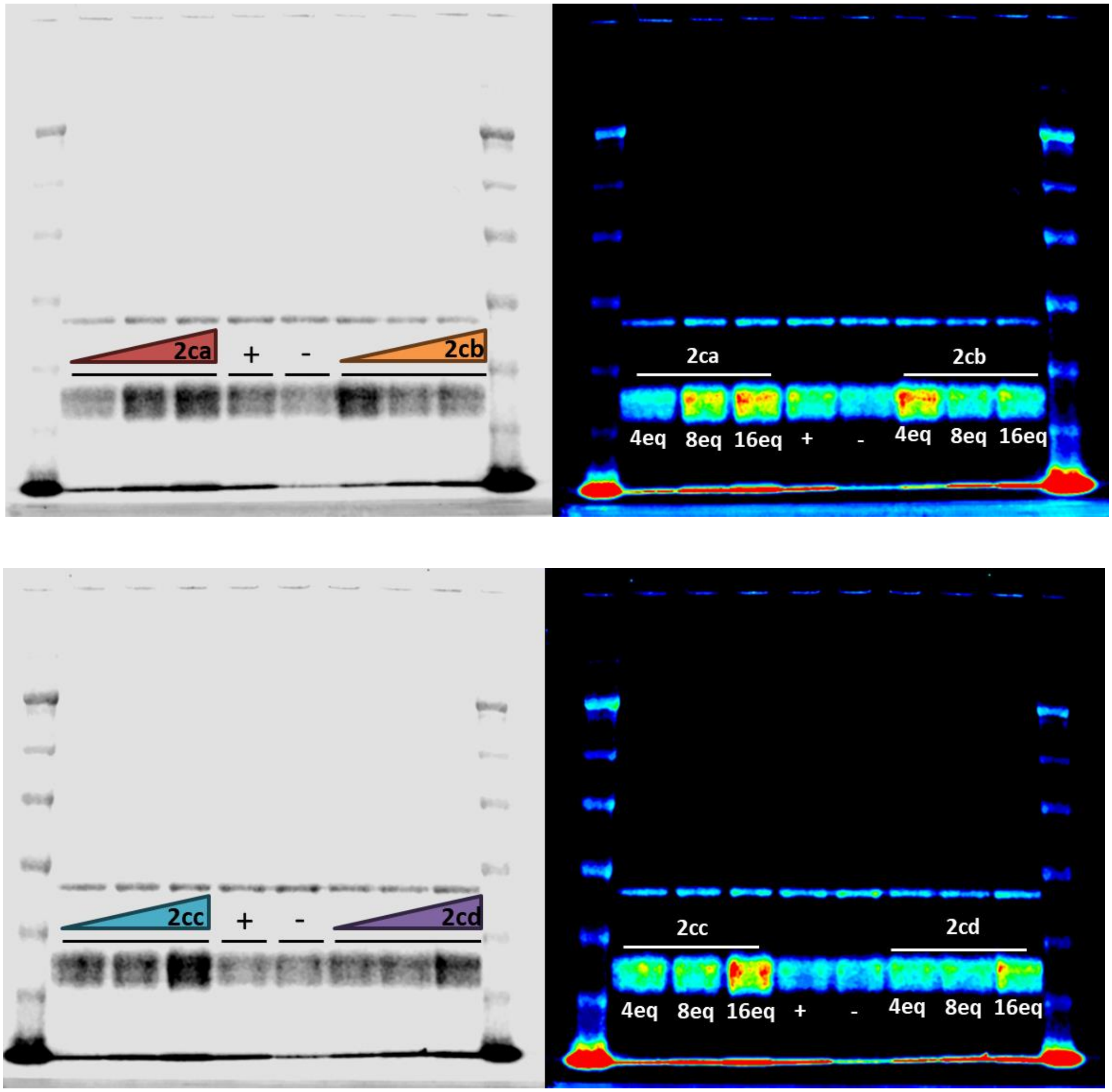

Intact Mass Protocol

To assess covalent bonding between PD1 and peptides containing electrophilic warheads, the intact His-tagged PD1 protein was incubated with peptides. The His-tagged PD1 protein (100 µg) was reconstituted in 50 µL of HEPES buffer (25 mM, pH 8) and 10 µL of stock solution was transferred into Eppendorf tubes. To simplify the mass spectrometry analysis, PD1 samples were treated with 0.5 µL PNGase F enzyme (as detailed above to remove all N-glycosylation sites) for 1 h at 50 °C. After cooling to room temperature, 7.5 µL of electrophilic peptide solutions (**2cb, 2cc, 2pa, 2pb**, 4 equiv.) dissolved in HEPES buffer and DMSO (99:01 vol/vol for solubility) were added, and the resulting incubation mixture totaled 18 µL. All steps of this process were carried out in a clean-air (HEPA) hood to prevent contamination (*e.g.* keratin.) and surfaces were sterilized with 70% ethanol. The mixtures of PD-1 (55 µM), PNGaseF, and peptide (220 µM) were incubated for 2 days at 40 °C and diluted to a final volume of 25 µL before analysis. Intact mass spectra were collected with an Orbitrap Fusion™ Lumos™ Tribrid™ Mass Spectrometer coupled to a Vanquish™ Neo UHPLC System (Thermo Scientific™). Samples (2 µL containing approximately 1.6 µg of PD1) were injected on a MAbPac Capillary reversed phase column (150 µm x 150 mm) and eluted using a mixture of 0.1% formic acid in water (solvent A) and 0.1% formic acid in 80% acetonitrile in water (solvent B). A solvent gradient of B:A mixture 3:97 was increased to 90:10 over 6 minutes at a flow rate of 2 µL/min and a column temperature of 60 °C. The EASY-Spray™ ion source parameters were set as follows: spray voltage 1800 V positive ion, ion transfer tube temp 305 °C, and a RF lens 75%. The detection parameters were set as follows: detector type Orbitrap 7.5K resolution, quad isolation, scan range 400–2000 (m/z), positive mode, FT Full scan AGC target 4×10^5 , normalized AGC target 100%, and cycle time of 3 seconds. The resolution was set to the lowest setting possible to shorten the transients to only use the high signal to noise beats before the protein dephases in the Orbitrap. Intact mass spectra were deconvoluted using BioPharma Finder (version 3.0 Thermo Scientific software). The default deconvolution method titled “Default SW ReSpect” was edited as follows: chromatogram m/z range was set from 400-2000, sensitivity was set to “High”, source spectra method was set to “Sliding Windows”, and the deconvolution algorithm was set to “ReSpect” due to the lack of isotopically resolved source spectra. The output mass range for deconvolution was set from 10,000 to 36,000 Da with peak model set to “Intact Protein” and all other parameters were default.

Intact Mass Analysis of PD-1 with Electrophilic ADAPTin Peptides.

Results from incubation of PD1 with **2pa** suggested that some covalent binding is taking place most predominantly on two glyco-forms of the protein (**Figure S24**). Incubation with **2pa** caused a decrease in the relative abundances of masses at 20,686 and 21,422 Da in the deconvoluted spectra of PD1 along with the appearance of two new masses outside of the PD1 mass range of 19,955-21,884. These new masses detected 23,285 and 24,023 of conjugated PD1 represent an addition of 2,600 corresponding to the oxidized form of **2pa** (methionine oxidation to sulfone). The intact mass deconvolution of **2pb** shows the appearance of a peak with a mass difference of 5114 (mass of **2pb** 2557 Da) compared to the control PD1 peak, representing the covalent binding of **2pb** to PD1 in a 2:1 manner (**Figure S25**).

Figure S24. A) Deconvoluted mirrored spectra output for a direct comparison between deconvoluted PD1 masses recorded after incubation with **2pa** (top) versus pure PD1 control (bottom). **B)** Zoomed in spectral region to show peaks of peptide **2pa** covalently bound to PD1 in a 1:1 manner. MW of **2pa** is 2570.83 Da, but an oxidized methionine sulfone was observed with a mass of 2600.14 Da.

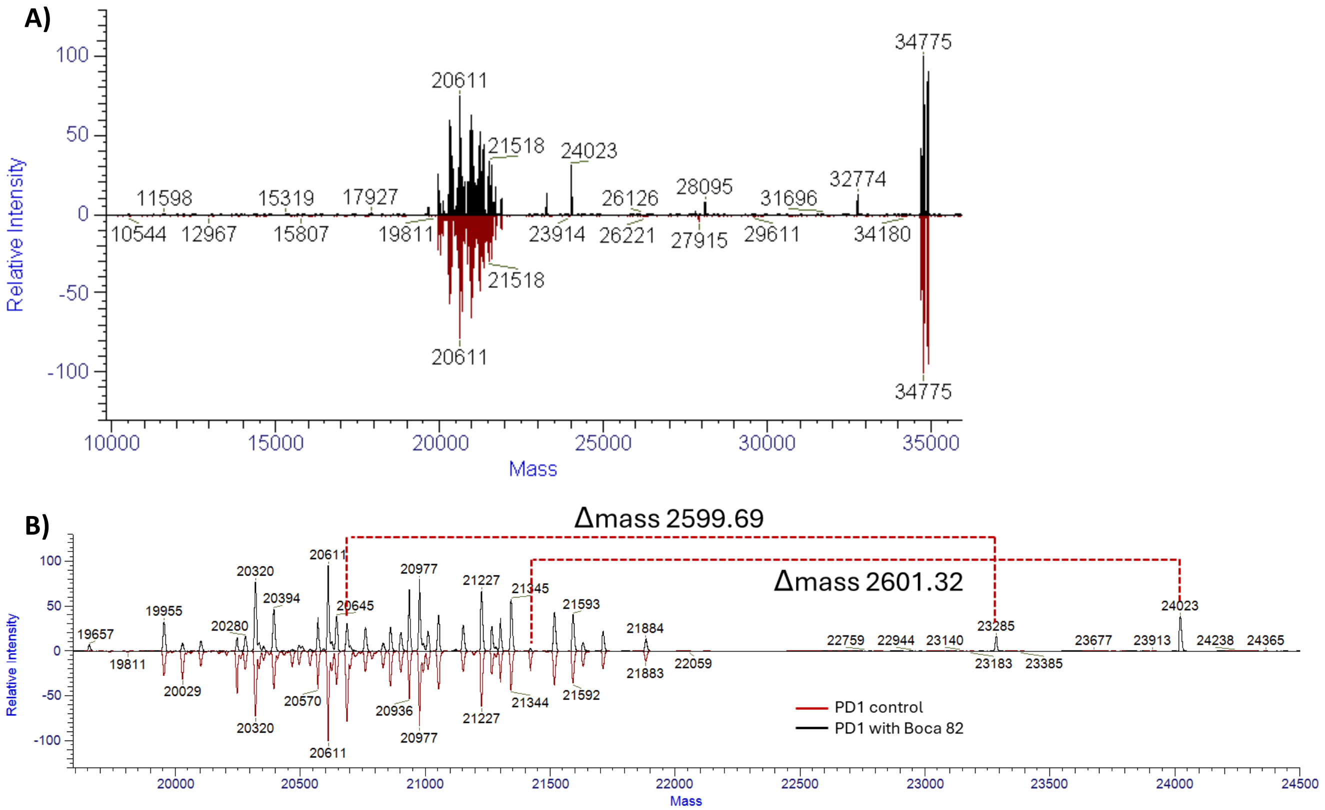

Figure S25. A) Deconvoluted mirrored spectra comparison output for a direct comparison between deconvoluted spectra of **2pb** (top) with respect to PD-1 control (bottom). **B)** Zoomed in spectral region to show peak of peptide **2pb** covalently bound to PD-1 in a 2:1 manner. MW of **2pb** is 2556.80 Da therefore a double addition would result in an additional mass of 5113.60 Da.

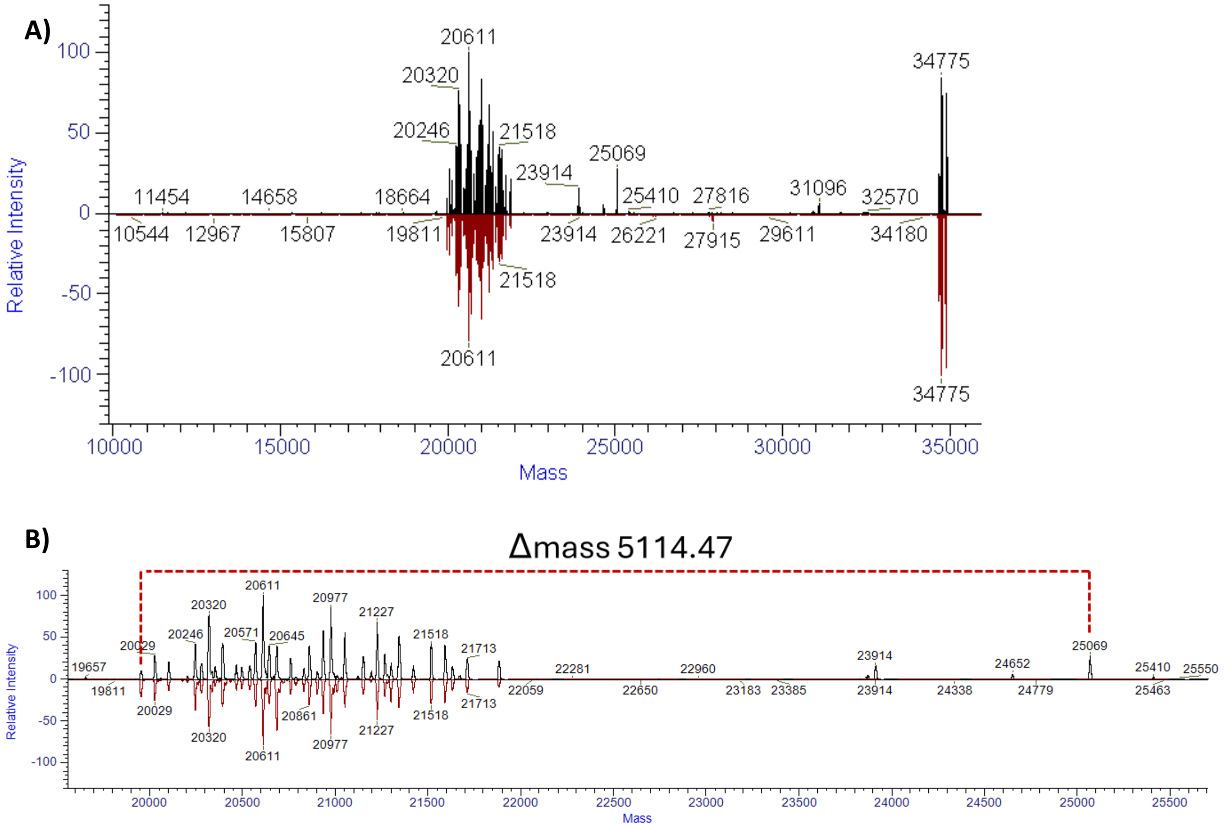

11. *In Vitro PBMC Binding Data*

<u>*In Vitro PBMC Binding Assay.*</u>

Human peripheral blood mononuclear cells (PBMCs) from healthy donors were activated for 48 h before being co-cultured using anti-CD2/28 monoclonal antibodies with IL-2 (2 ng/mL) as we have previously reported.[SI-9] To test for PBMCs proliferation, on day 3, cells were washed and incubated with 1 µm of carboxyfluorescein succinimidyl ester (CFSE) (BioLegend, cat no. 423801) at 37 °C for 7 min then thoroughly washed with staining buffer before in vitro co-culture. The proliferation rate of lymphocytes was determined by assessing the reduction of the intensity of the fluorescent cell-permeable dye CFSEPBMCs culture media were then washed and cells were treated for 6 h with *ADAPTins* **2c/2p**, the electrophilic analogs **2ca-cd, 2pa-pb** (at 5 µM) and with pembrolizumab (at 100 nM as positive control). Untreated cells were used as negative control to determine the baseline level of PD1 expression. After 6 h of treatment, PBMCs were washed 1 time with PBS, and further incubated in complete media for another 24 h. Culture media was collected to be used for cytokine secretion assay. PBMCs were washed 1 time with PBS and collected for a flow cytometry analysis. Percent PD1$^+$ was gated on CD45$^+$ and fractionated for CD3$^+$/CD8$^+$. The percentage of PD1 blockade was calculated in each case from the untreated PBMC baseline level.

Figure S26. Selected example of *In Vitro* PD1 blocking activity of *ADAPTins* **2** on healthy PBMCs measured by flow cytometry.

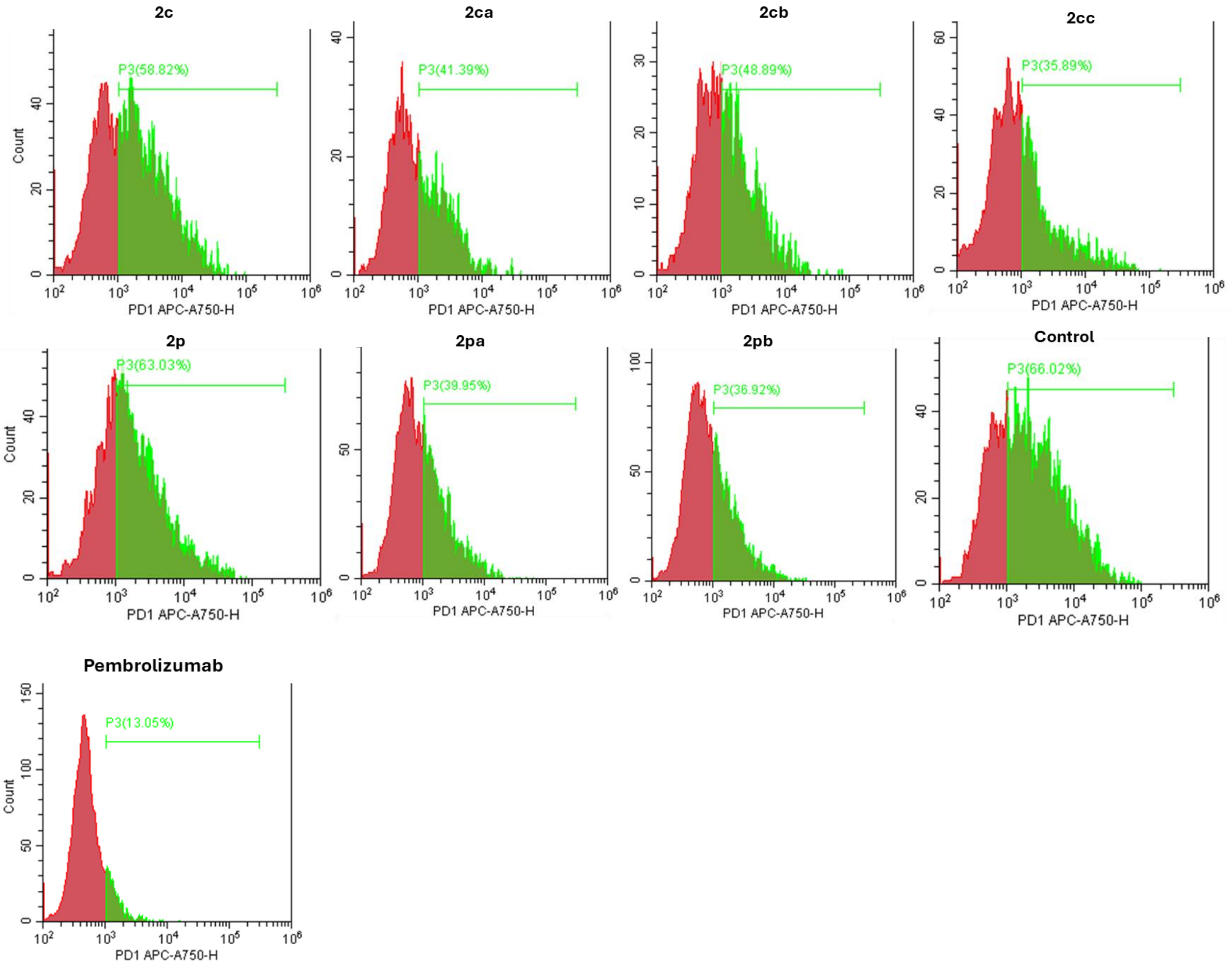

Ex Vivo Cytokine Secretion Assay.

Normal PBMCs (from healthy donor) and exhausted PBMCs isolated from the blood sample of a melanoma patient were activated for 48 h before using anti-CD2/28 monoclonal antibodies with IL-2 (2 ng/mL) as mentioned above. After 48 h, PBMCs were washed and incubated with a series of *ADAPTins* for 6 h. After treatment, media supernatant was collected for cytokine release measurements using the LEGENDplex™ human CD8/NK (13-plex) panel (BioLegend, cat no. 740267). Briefly, samples were mixed with assay buffer and human (13-plex) mixed-beads and plated on the 96-well filter plate. Samples were incubated for 2 h at room temperature, washed, and resuspended beads with detection antibodies according to manufacturer's protocol. Samples were transferred to a 96-well plate to read on a flow cytometer. The flow cytometer and data acquisition software setting were used according to the manufacturer's instructions. Flow analysis was obtained using a CytoFLEX-S flow cytometer equipped with 3 lasers (488 nm blue, 405 nm violet, and 638 nm from Beckman).

Table S9. Raw data of cytokine secretion assay (n=2).

**Cytokine assay (pg/ml)**	**IL-2**		**TNF-α**		**IFN-γ**		**Perforin**	
e-PBMC	157.14	151.35	312.91	318.47	2384.78	2489.13	218.3	220.68
**2c**	0.44	266.63	483.47	1150.47	4131.52	7535.05	217.39	381.31
**2ca**	254.01	250.92	889.42	1016.29	6460.88	6434.14	321.51	351.76
**2cb**	219.79	287.5	654.09	1278.69	6587.71	7478.07	351.77	428.77
Pembro	0.44	275.42	500.12	1224.22	4359.77	7617.66	227.36	438.61

**SD**	**IL-2**	**TNF-α**	**IFN-γ**	**Perforin**
e-PBMC	4.094148	3.931514	73.78659	1.682914
**2c**	188.2248	471.6402	2406.659	115.9089
**2ca**	2.18496	89.71064	18.90804	21.38998
**2cb**	47.8782	441.6589	629.5796	54.44722
Pembro	194.4402	512.016	2303.676	149.3763

12. *ADAPTin NMR Analysis and Selected Spectra*

Table S10. NMR Chemical Shift Deviations (CSD, ppm) calculated using random coil values reported by Poulsen.[SI-10]

	Scaffold (R) **1a**	Mimic **1b**	Scaffold (B) **2a**	Mimic **2b**
Res	**H_N Chemical shift deviations from RC values**			
R	n.d	n.d	n.d	n.d
W	0.97	0.91	0.85	1
V	2.07	1.91	1.9	2.07
**A/W**	1.17	0.84	0.36	0.43
**R**	n.a.	n.a.	0	0.15
L1	0.19	0.45	-0.23	-0.11
L2	0.26	0.04	-0.14	0.23
L3	-0.96	-0.02	-0.05	0.33
L4	0.02	0.25	0.03	0.53
L5	0.11	-0.58	-0.06	0.5
L6	0.04	0.16	-0.1	0.6
L7	0.01	-0.25	-0.3	-0.07
L8	0.01	-0.23	-0.17	0.16
**F/G**	-0.12	0.23	-0.09	0.23
**D/G**	-1.33	0.29	0.25	0.48
**Y**	n.a.	n.a.	0.21	0.62
**W**	0.2	0.97	0.75	0.98
V	2.06	1.97	2.03	1.96
W	1.08	0.47	0.92	1.12
E	0.01	-0.09	-0.04	0.05

	Scaffold (R) **1a**	Mimic **1b**	Scaffold (B) **2a**	Mimic **2b**
Res	**H_α Chemical shift deviations from RC values**			
R	-0.59	-0.76	-0.77	-0.75
W	0.74	0.59	0.59	0.63
V	0.98	0.79	0.61	0.65
**A/W**	-0.25	0.04	-0.08	-0.08
**R**	n.a.	n.a.	-0.04	-0.02
L1	0.19	0.19	-0.02	0.44
L2	-0.06	0.04	-0.11	0.25
L3	-0.22	-0.10	-0.08	0.32
L4	-0.09	-0.34	-0.09	0.17
L5	-0.04	-0.155	-0.09	-0.72
L6	-0.03	-0.14	-0.12	0.15
L7	0.02	-0.12	-0.02	0.24
L8	-0.02	-0.11	-0.05	-0.145
**F/G**	-0.23	0.06	-0.01	0.06
**D/G**	-0.46	0.07	0.42	0.65
**Y**	n.a.	n.a.	0.17	0.25
**W**	0.45	0.29	0.61	0.71
V	1.05	0.78	1.06	1.16
W	-0.02	-0.07	0.05	0.05
E	0.23	0.04	0.07	0.14

Figure S27. Histograms of CSD for hairpins **1a,b** (Regular)and **2a,b** (bulged) in water/DMSO-d_6 (90:10, v/v) at 291 K

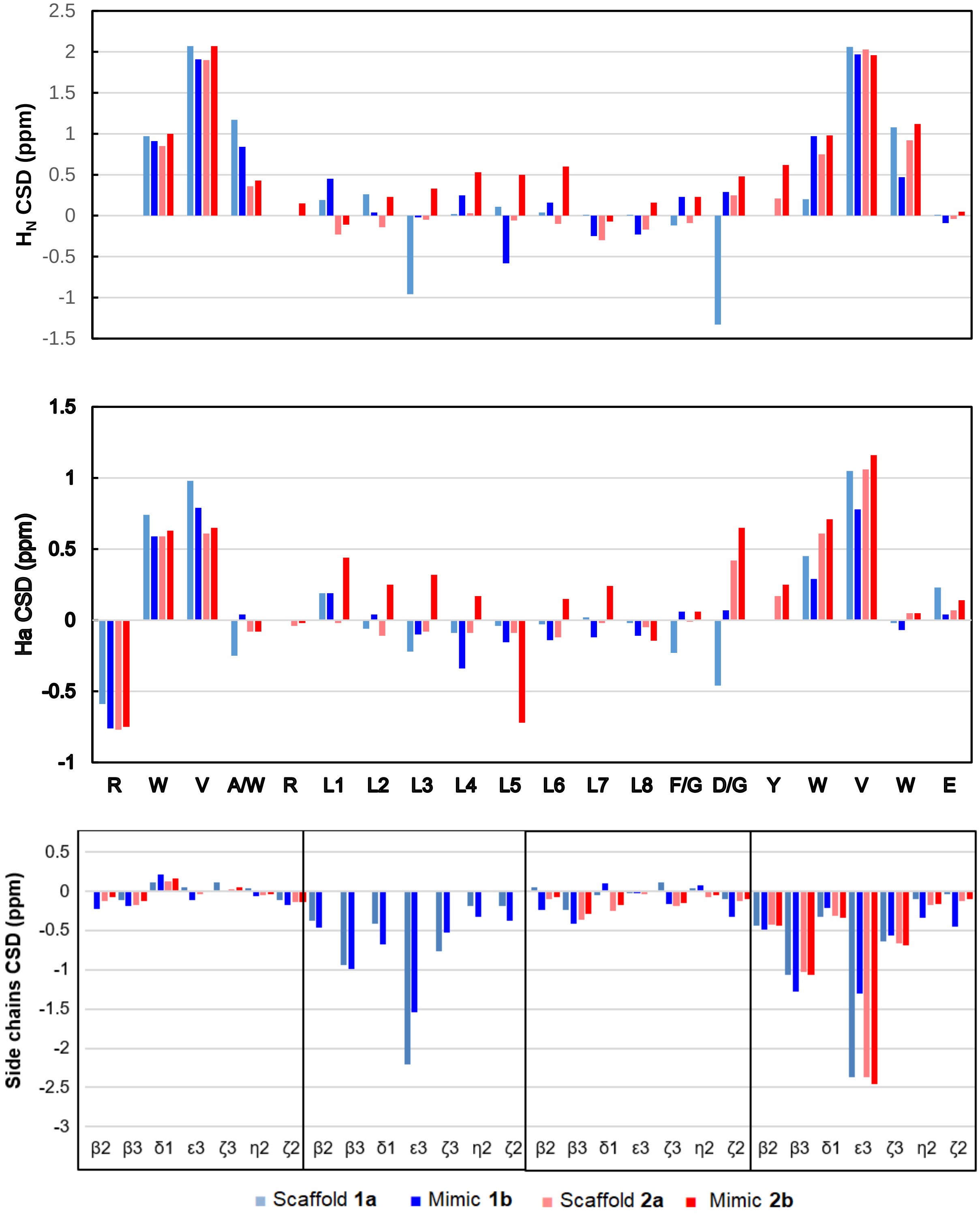

Scaffold 1a: RWVW-GGGGKKGGGG-WVWE (300 mg, 0.13 mmol of Wang resin) Yield: 70%

NMR chemical shift (δ, ppm): Samples were prepared in a phosphate buffer (15 mM) /D_2O mixture (90:10 v/v) to improve solubility and spectral resolution.

Solution of peptide **1a** (5 mM) was prepared for ^{1}H, COSY, TOCSY, NOESY, ^{1}H-^{13}C HSQC, and HMBC analysis (500 MHz) and 20 mM solution of **1a** was prepared ^{13}C analysis (400 MHz).

Res (pos)	δH^{N}	δH^{α}	δH^{β}	δH other Side chains	δC (type)
R(1)	n.d.	3.56	$H_{\beta2}$: 1.62 $H_{\beta3}$: 1.81	$H_{\gamma2}$: 1.41, $H_{\gamma2}$: 1.37, H_{δ}: 3.13, H_{ε}: 7.20	C_{α}: 52.7, C_{β}: 27.4, C_{γ}: 21.9, C_{δ}: 40.3
W(2)	9.03	5.46	$H_{\beta2}$: 3.35 $H_{\beta3}$: 3.14	$H_{\delta1}$: 7.39, $H_{\varepsilon1}$: 10.18, $H_{\zeta2}$: 7.39, $H_{\eta2}$: 7.22, $H_{\zeta3}$: 7.28, $H_{\varepsilon3}$: 7.71	C_{α}: 54.4, C_{β}: 27.6, $C_{\delta1}$: 125.5, $C_{\varepsilon3}$: 117.8, $C_{\zeta2}$: 112.6, $C_{\zeta3}$: 120.1, $C_{\eta2}$: 121.4
V(3)	9.95	4.83	H_{β}: 2.13	H_{γ}: 0.93	C_{α}: 58.3, C_{β}: 32.8, C_{γ}: 17.7
W(4)	9.08	4.33	$H_{\beta2}$: 2.96 $H_{\beta3}$: 2.31	$H_{\delta1}$: 6.84, $H_{\varepsilon1}$: 9.98, $H_{\zeta2}$: 7.31, $H_{\eta2}$: 7.05, $H_{\zeta3}$: 7.71, $H_{\varepsilon3}$: 6.40	C_{α}: 54.2, C_{β}: 25.7, $C_{\delta1}$: 124.6, $C_{\varepsilon3}$: 119.1, $C_{\zeta2}$: 111.8, $C_{\zeta3}$: 117.8, $C_{\eta2}$: 122.1
G(5)	8.28	3.56; 4.18	n.a.	n.a.	C_{α}: 42.4
G(6)	8.32	4.01	n.a.	n.a.	C_{α}: 42.4
G(7)	8.32	4.01	n.a.	n.a.	C_{α}: 424
G(8)	8.32	4.01	n.a.	n.a.	C_{α}: 424
K(9)	8.36	4.29	1.86	H_{γ}: 1.45, H_{δ}: 1.72, $H_{\varepsilon1}$: 3.01	C_{α}: 54.2, C_{β}: 29.9, C_{γ}: 21.8, C_{δ}: 26.3, C_{ε}: 39.1
K(10)	8.47	4.30	1.86	H_{γ}: 1.45, H_{δ}: 1.72, $H_{\varepsilon1}$: 3.01	C_{α}: 54.2, C_{β}: 29.8, C_{γ}: 21.8, C_{δ}: 26.3, C_{ε}: 39.1
G(11)	8.28	4.01	n.a.	n.a.	C_{α}: 42.4
G(12)	8.32	4.01	n.a.	n.a.	C_{α}: 42.4
G(13)	8.22	3.70	n.a.	n.a.	C_{α}: 41.8
G(14)	6.91	**3.07; 3.62**	n.a.	n.a.	C_{α}: 42.1
W(15)	8.14	5.14	$H_{\beta2}$: 3.41 $H_{\beta3}$: 3.03	$H_{\delta1}$: 7.27, $H_{\varepsilon1}$: 10.10, $H_{\zeta2}$: 7.40, $H_{\eta2}$: 7.28, $H_{\zeta3}$: 7.60, $H_{\varepsilon3}$: 7.64	C_{α}: 54.4, C_{β}: 27.6, $C_{\delta1}$: 125.5, $C_{\varepsilon3}$: 117.6, $C_{\zeta2}$: 112.6, $C_{\zeta3}$: 118.4, $C_{\eta2}$: 120.1
V(16)	9.89	4.91	2.18	0.96	C_{α}: 58.1, C_{β}: 32.8, C_{γ}: 18.5
W(17)	8.85	4.60	$H_{\beta2}$: 2.89 $H_{\beta3}$: 2.18	$H_{\delta1}$: 6.97, $H_{\varepsilon1}$: 10.16, $H_{\zeta2}$: 7.46, $H_{\eta2}$: 7.14, $H_{\zeta3}$: 7.18, $H_{\varepsilon3}$: 6.50	C_{α}: 53.8, C_{β}: 27.6, $C_{\delta1}$: 125.9, $C_{\varepsilon3}$: 117.8, $C_{\zeta2}$: 112.4, $C_{\zeta3}$: 119.5, $C_{\eta2}$: 121.4
E(18)	8.20	4.24	1.96	2.26	C_{α}: 53.1, C_{β}: 28.4, C_{γ}: 30.2

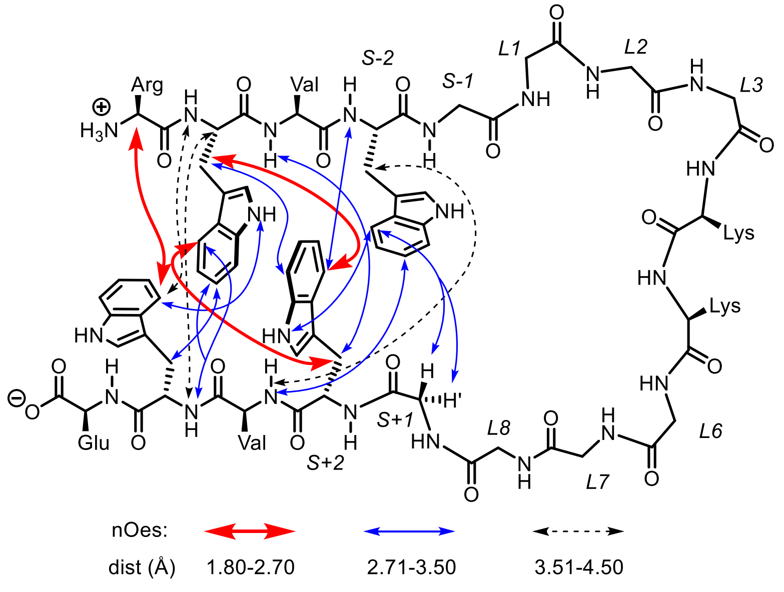

Figure S28. Schematic representation of the most relevant inter-residue NOESY cross-peak correlations for **1a.**

Scaffold 2a: RW-VAR-GGGGKKGGFD-YWV-WE (300 mg, 0.13 mmol of Wang resin) Yield: 70%

NMR chemical shift (δ, ppm): Sample of scaffold **2a** was prepared in a mixture of DMSO-d^6 / H_2O (10:90 v/v) for the following ^{1}H, TOCSY, NOESY, and ^{1}H-^{13}C HSQC analysis.

Res(pos)	δH^N	δH^α	δH^β	δH other Side chains	δC (type)
R(1)		3.48	$H^{\beta2}$: 1.72, $H^{\beta3}$: 1.55	$H^{\gamma2}$: 1.35, $H^{\gamma3}$: 1.29, H^{δ}: 3.06, H^{ε}: 7.19	C_α:52.5, C_β:27.7, C_γ: 23.0, C_δ: 40.5
W(2)	9.00	5.32	$H^{\beta2}$: 3.21, $H^{\beta3}$: 3.08	$H^{\delta1}$: 7.41, $H^{\varepsilon1}$: 10.37, $H^{\zeta2}$: 7.37, $H^{\eta2}$: 7.21, $H^{\zeta3}$: 7.21, $H^{\varepsilon3}$: 7.61	C_α: 54.2, C_β: 27.7, $C_{\delta1}$: 125.1, $C_{\varepsilon3}$: 117.6, $C_{\zeta2}$: 111.9, $C_{\zeta3}$: 119.8, $C_{\eta2}$: 121.9
V(3)	9.68	4.52	1.96	$H^{\gamma1}$: 0.85, $H^{\gamma2}$: 0.77	C_α: 58.1, C_β: 27.7, $C_{\gamma1}$: 18.5, $C_{\gamma2}$: 17.9
A(4)	8.50	4.10	0.68		C_α: 48.9, C_β: 18.1
R(5)	8.48	4.30	1.61	$H^{\gamma2}$: 1.49, $H^{\gamma3}$: 1.30, H^{δ}: 3.10, H^{ε}: 7.15	C_α: 52.4, C_β: 29.3, C_γ: 24.3, C_δ: 40.6
G(6)	8.30	4.01			C_α: 42.4
G(7)	8.25	3.92			C_α: 42.4
G(8)	8.32	3.94			C_α: 42.4
G(9)	8.34	3.90			C_α: 42.4
K(10)	8.21	4.24	$H^{\beta2}$: 1.85, $H^{\beta3}$: 1.74	$H^{\gamma2}$: 1.42, $H^{\gamma3}$ 1.37, H^{δ}: 1.65, H^{ε}: 2.95, H^{ζ}: 7.54	C_α: 53.8, C_β: 30.2, C_γ: 22.2, C_δ: 26.3, C_ε: 39.4
K(11)	8.36	4.22	$H^{\beta2}$: 1.82, $H^{\beta3}$: 1.75	H^{γ}: 1.40, H^{δ}: 1.65, H^{ε}: 2.95, H^{ζ}: 7.54	C_α:52.4, C_β: 30.2, C_γ: 22.2, C_δ: 26.3, C_ε: 39.4
G(12)	8.18	3.91			C_α: 42.4
G(13)	8.13	3.89			C_α: 42.4
F(14)	8.04	4.60	$H^{\beta2}$: 3.06, $H^{\beta3}$: 2.93	H^{δ}: 7.17, H^{ε}: 7.23, H^{ζ}: 7.26	C_α: 54.7, C_β: 37.2, C_δ: 129.2, C_ε: 127.1, C_ζ: 128.7
D(15)	8.41	4.85	2.55		C_α: 50.0, C_β: 36.1
Y(16)	8.18	4.63	2.94	H^{δ}: 6.84, H^{ε}: 6.58	C_α: 54.7, C_β: 36.8, C_δ: 130.7, C_ε: 115.3
W(17)	8.43	5.20	$H^{\beta2}$: 3.24, $H^{\beta3}$: 2.89	$H^{\delta1}$: 7.03, $H^{\varepsilon1}$: n.d., $H^{\zeta2}$: 7.38, $H^{\eta2}$: 7.18, $H^{\zeta3}$: 6.99, $H^{\varepsilon3}$: 7.61	C_α: 54.4, C_β: 29.0, $C_{\delta1}$: 124.6, $C_{\varepsilon3}$: 117.6, $C_{\zeta2}$: 111.9, $C_{\zeta3}$: 119.3, $C_{\eta2}$: 121.9
V(18)	9.67	4.87	2.12	$H^{\gamma1}$: 0.99, $H^{\gamma2}$: 0.93	C_α: n.d., C_β: 30.2, $C_{\gamma1}$: 18.7, $C_{\gamma2}$: 18.1
W(19)	8.76	4.59	$H^{\beta2}$: 2.91, $H^{\beta3}$: 2.22	$H^{\delta1}$: 6.97, $H^{\varepsilon1}$: 10.12, $H^{\zeta2}$: 7.39, $H^{\eta2}$: 7.08, $H^{\zeta3}$: 6.52, $H^{\varepsilon3}$: 5.28	C_α: 54.0, C_β: 29.0, $C_{\delta1}$: 125.2, $C_{\varepsilon3}$: n.d., $C_{\zeta2}$: 111.9, $C_{\zeta3}$: 119.3, $C_{\eta2}$: 121.2
E(20)	8.06	4.17	$H^{\beta2}$: 1.91, $H^{\beta3}$: 1.59	H^{γ}: 2.17	C_α: 54.0, C_β: 27.7, C_γ: 30.1

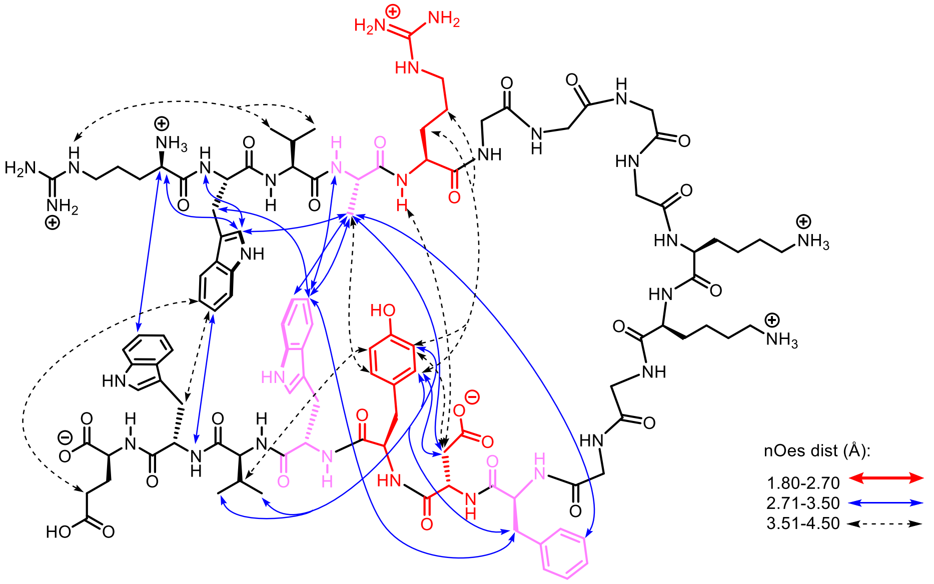

Figure S29*.* Schematic representation of the most relevant inter-residue NOESY cross-peak correlations for **2a.**

Table S11. Long range NOESY cross-peaks for peptide RW-VAR-GGGGKKGGFD-YWV-WE **(2a)**

(Strong: 1.80 – 2.70 Å; Medium: 2.71 – 3.50 Å; Weak: 3.51 – 4.50 Å)

Residue I	Residue II	peak	Integration	Distance (Å)
17Trp H^{β}	17Trp H^{β}	S	100.00	1.80
1Arg H^{α}	2Trp $H^{\delta1}$	M	2.07	3.44
1Arg H^{ε}	3Val $H^{\gamma1,\ \gamma2}$	W, W	1.21, 1.71	3.76, 3.55
1Arg H^{α}	19Trp $H^{\eta2}$	M	5.18	2.95
2Trp H^{N}	2Trp $H^{\delta1}$	M	8.16	2.73
2Trp $H^{\beta2}$	17Trp $H^{\zeta3}$	M	3.73	3.11
2Trp $H^{\delta1}$	4Ala H^{β}	M	2.09	3.43
2Trp $H^{\eta2}$	19Trp H^{N}	M	4.66	3.00
2Trp $H^{\eta2}$	19Trp $H^{\beta3}$	W	1.57	3.60
2Trp $H^{\zeta3}$	14Phe $H^{\beta3}$	M	3.19	3.20
2Trp $H^{\zeta3}$	20Glu H^{γ}	W	1.33	3.70
4Ala H^{N}	17Trp $H^{\zeta3}$	M	2.79	3.27
4Ala H^{β}	14Phe H^{ε}	M	2.41	3.35
4Ala H^{β}	15Asp H^{β}	M	5.41	2.93
4Ala H^{β}	16Tyr H^{δ}	W	0.70	4.11
4Ala H^{β}	17Trp $H^{\zeta3}$	M	5.38	2.93
4Ala H^{β}	17Trp $H^{\eta2}$	M	4.29	3.04
5Arg H^{N}	15Asp H^{β}	W	1.82	3.51
5Arg H^{β}	16Tyr H^{δ}	W	0.21	5.03
5Arg H^{β}	16Tyr H^{ε}	W	0.83	4.00
5Arg $H^{\gamma2,\ \gamma3}$	16Tyr H^{δ}	W, W	0.52, 0.59	4.32, 4.24
5Arg $H^{\gamma2,\ \gamma3}$	16Tyr H^{ε}	M, W	2.93, 0.76	3.24, 4.06
16Tyr H^{ε}	14Phe $H^{\beta2,\ \beta3}$	M, M	3.90, 5.52	3.09, 2.92
16Tyr H^{δ}	15Asp H^{β}	M	2.43	3.35
16Tyr H^{ε}	15Asp H^{β}	W	0.74	4.08
16Tyr H^{δ}	18Val H^{β}	W	0.95	3.91
16Tyr H^{δ}	18Val $H^{\gamma1,\ \gamma2}$	M, M	3.65, 4.70	3.12, 3.00
16Tyr H^{ε}	18Val $H^{\gamma1,\ \gamma2}$	S, M	12.33, 8.16	2.55, 2.73

^{1}H NMR (500 MHz) and ^{13}C NMR (100 MHz) of **1a** in water/DMSO-d_6 (90:10, vol/vol).

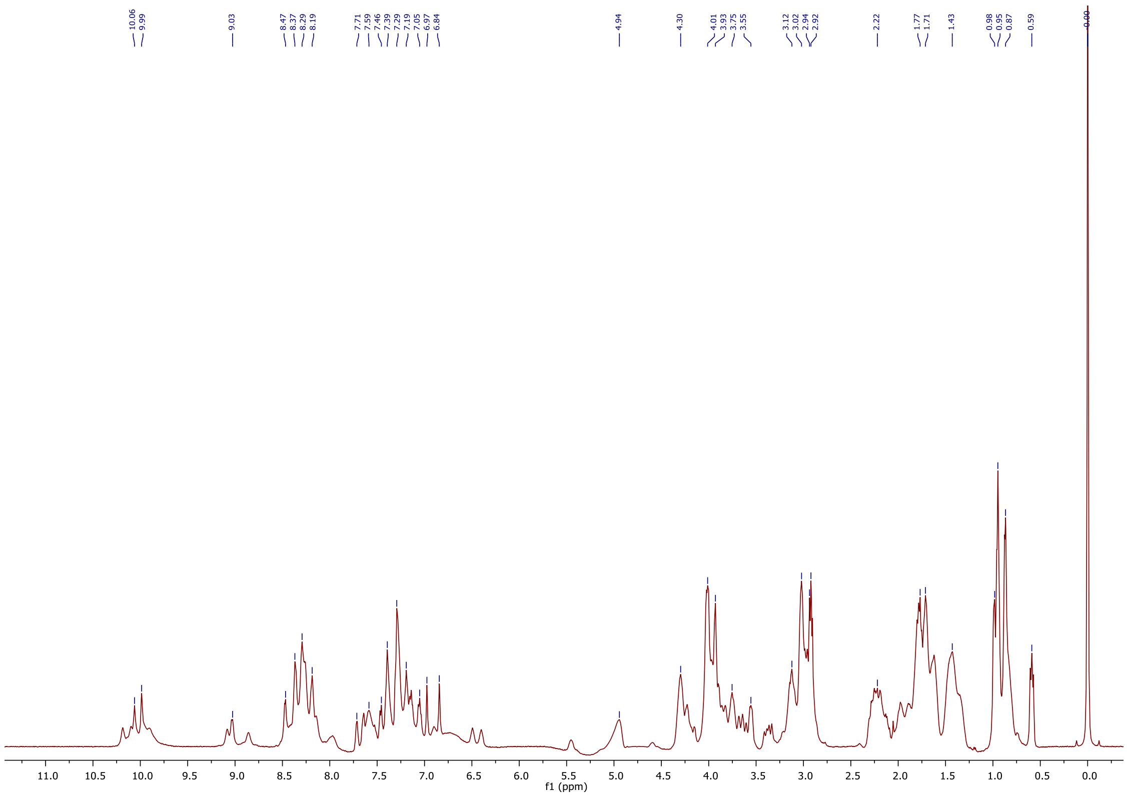

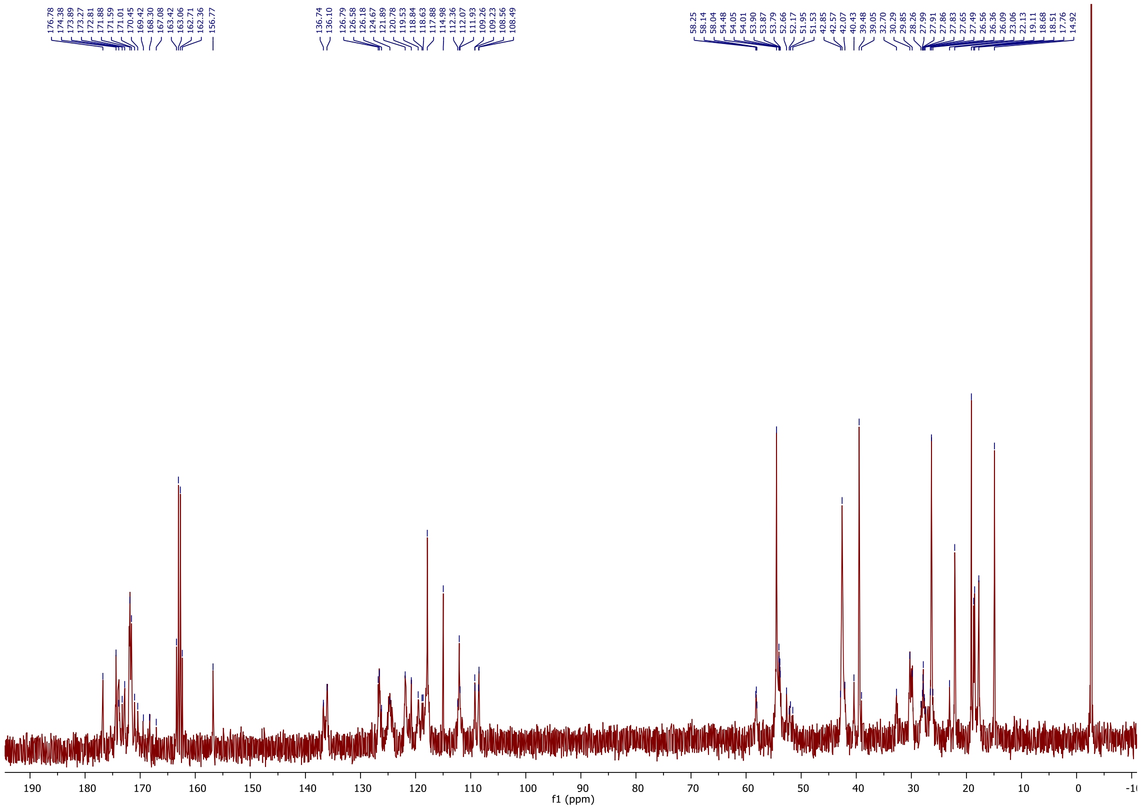

^{1}H ^{13}C-HSQC and ^{1}H ^{13}C-HMBC (500 MHz) of **1a** in water/DMSO-d_6 (90:10, vol/vol).

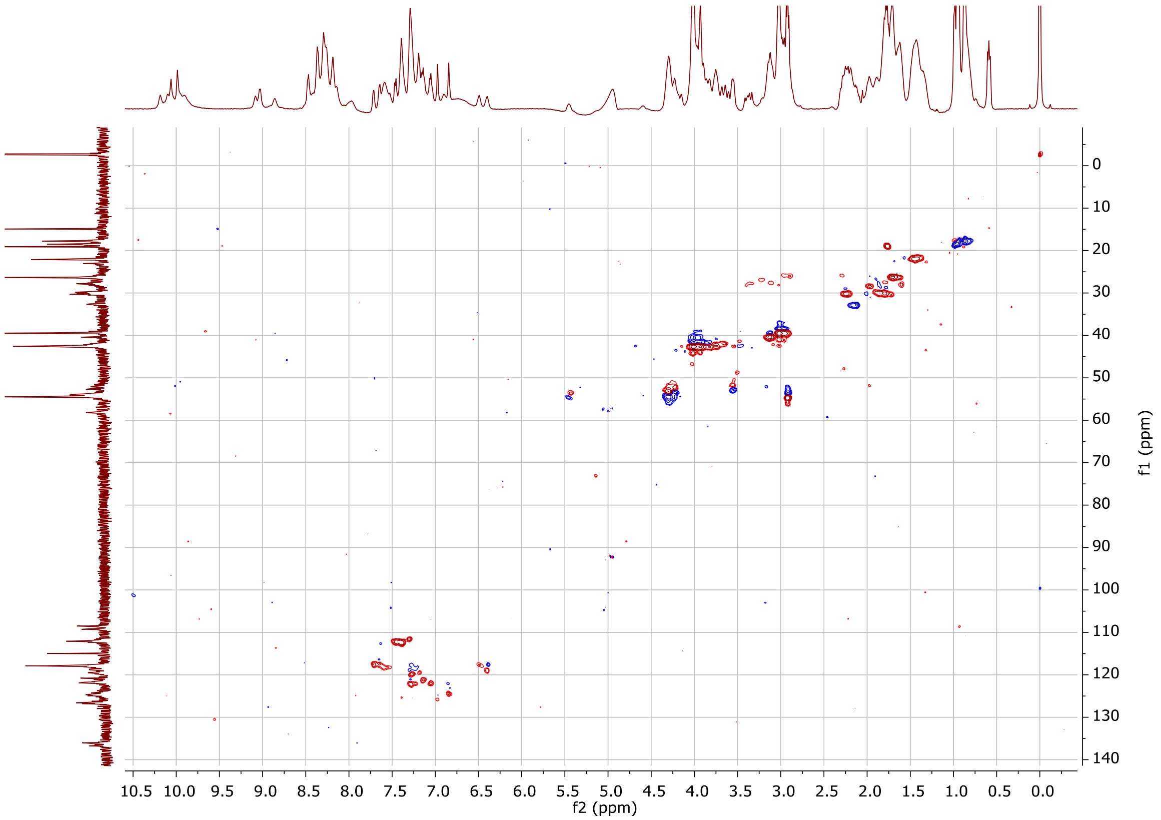

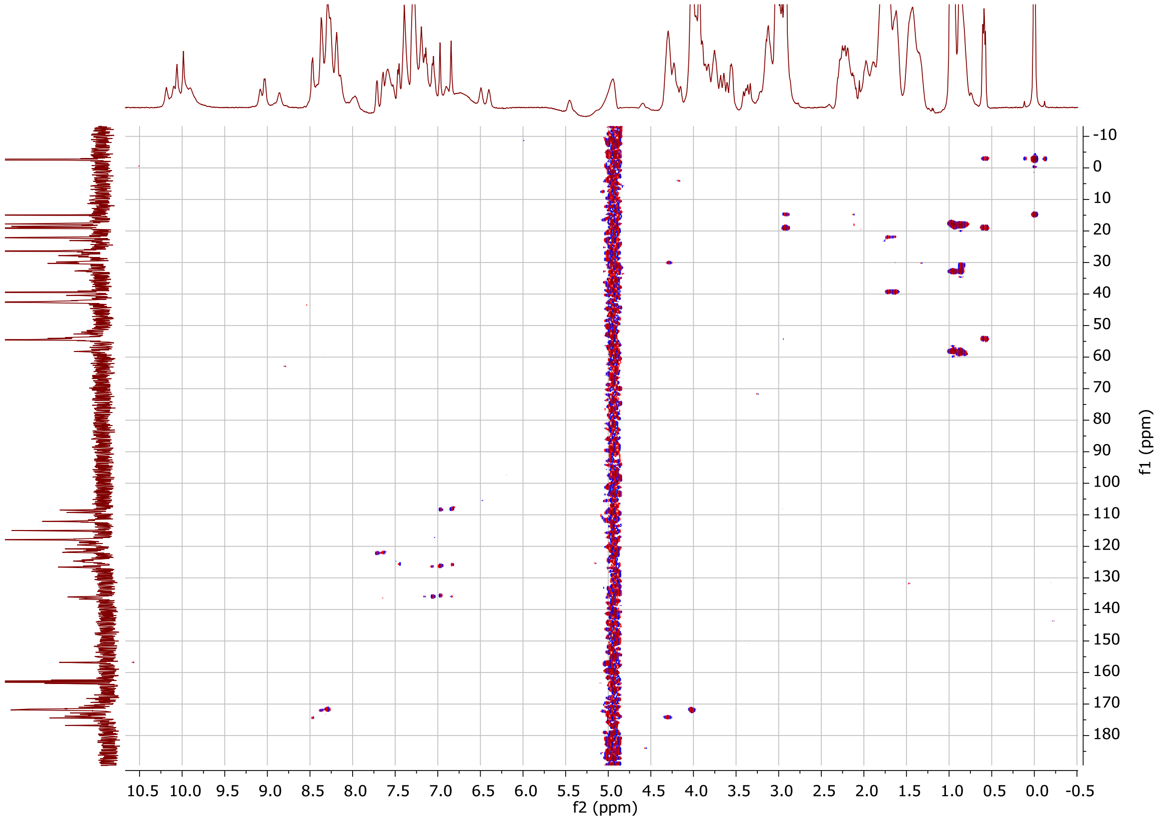

TOCSY and NOESY (500 MHz) of **1a** in water/DMSO-d_6 (90:10, vol/vol).

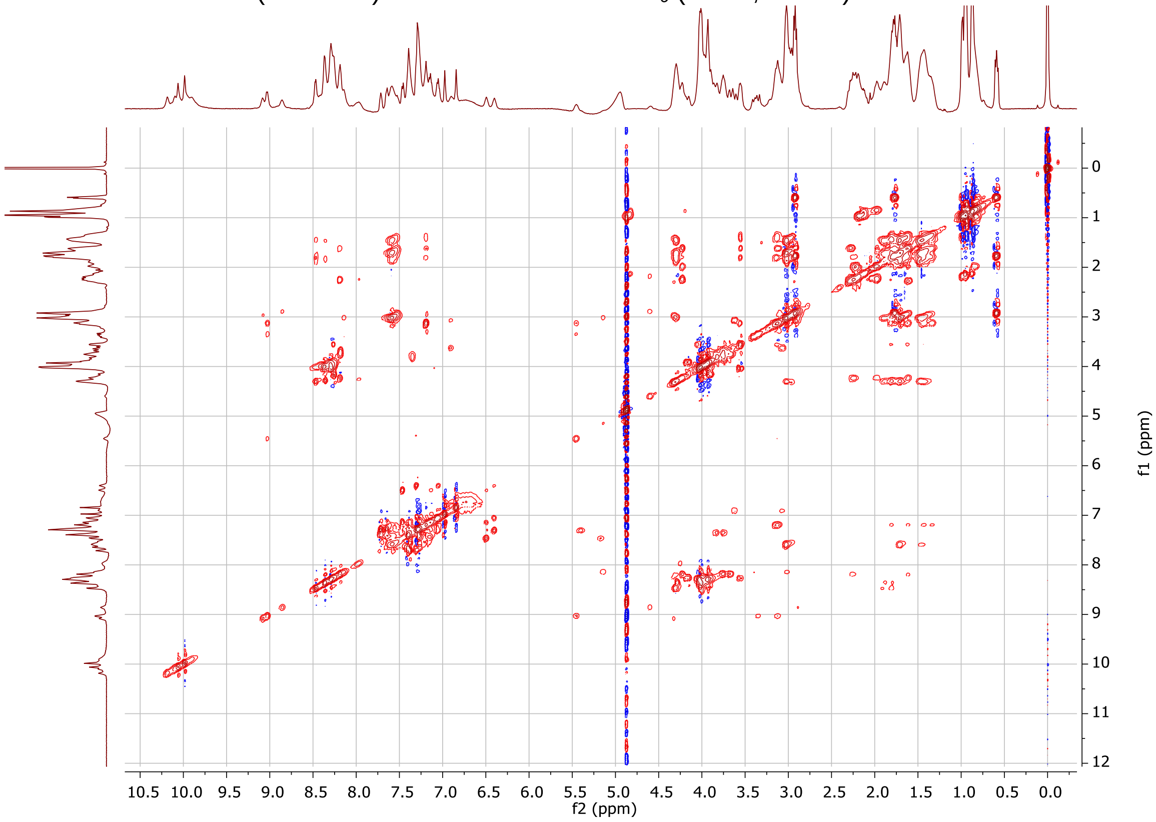

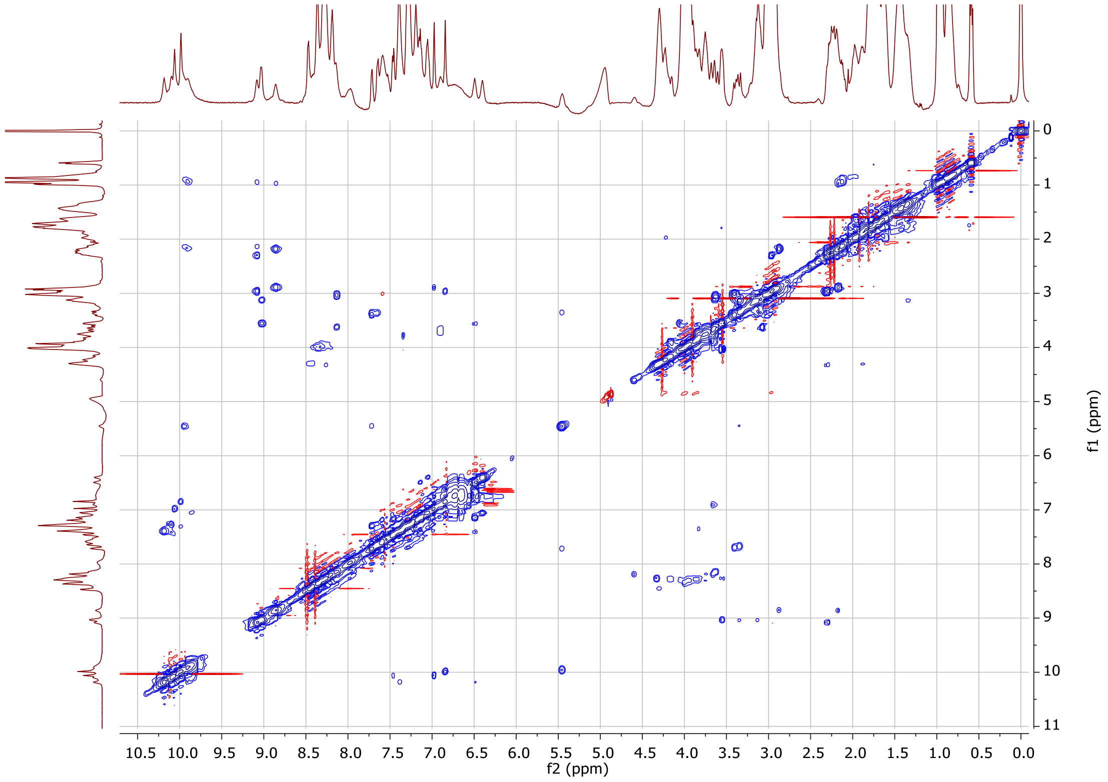

TOCSY and NOESY Overlay of the fingerprint region (500 MHz) of **1a** in water/DMSO-d_6 (90:10, vol/vol).

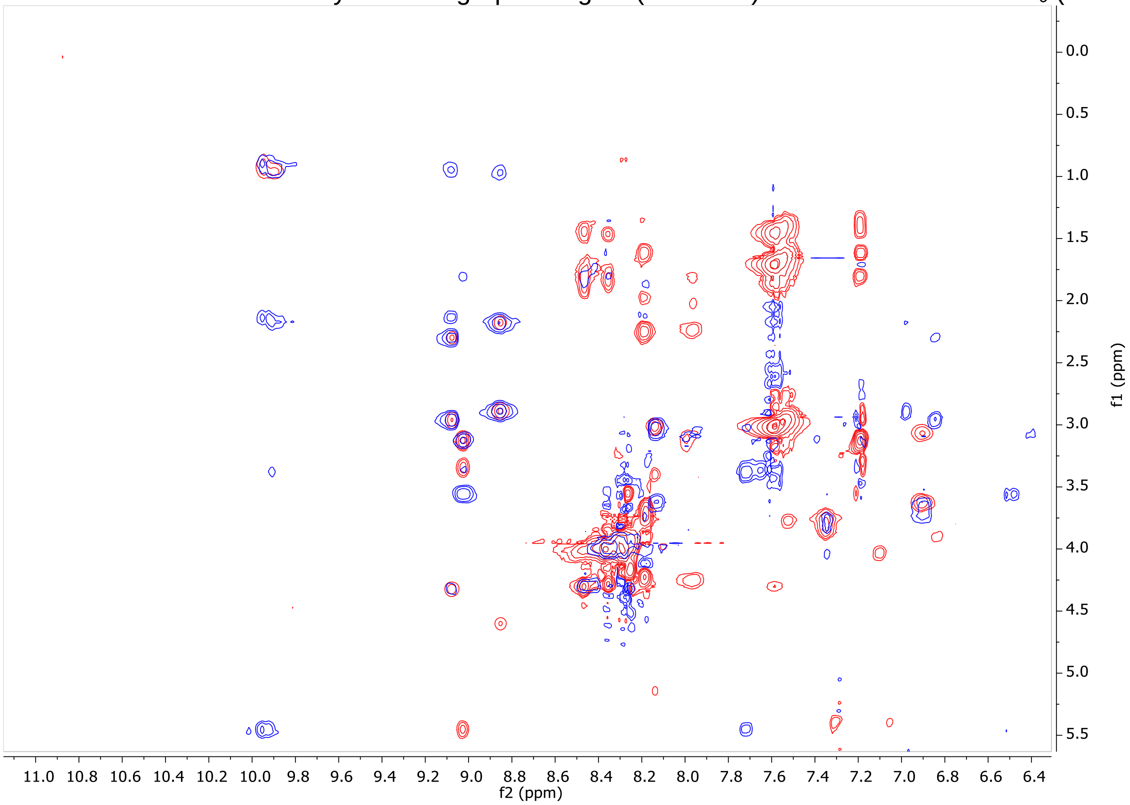

Selected NOESY correlations (500 MHz) of **1a** in water/DMSO-d_6 (90:10, vol/vol).

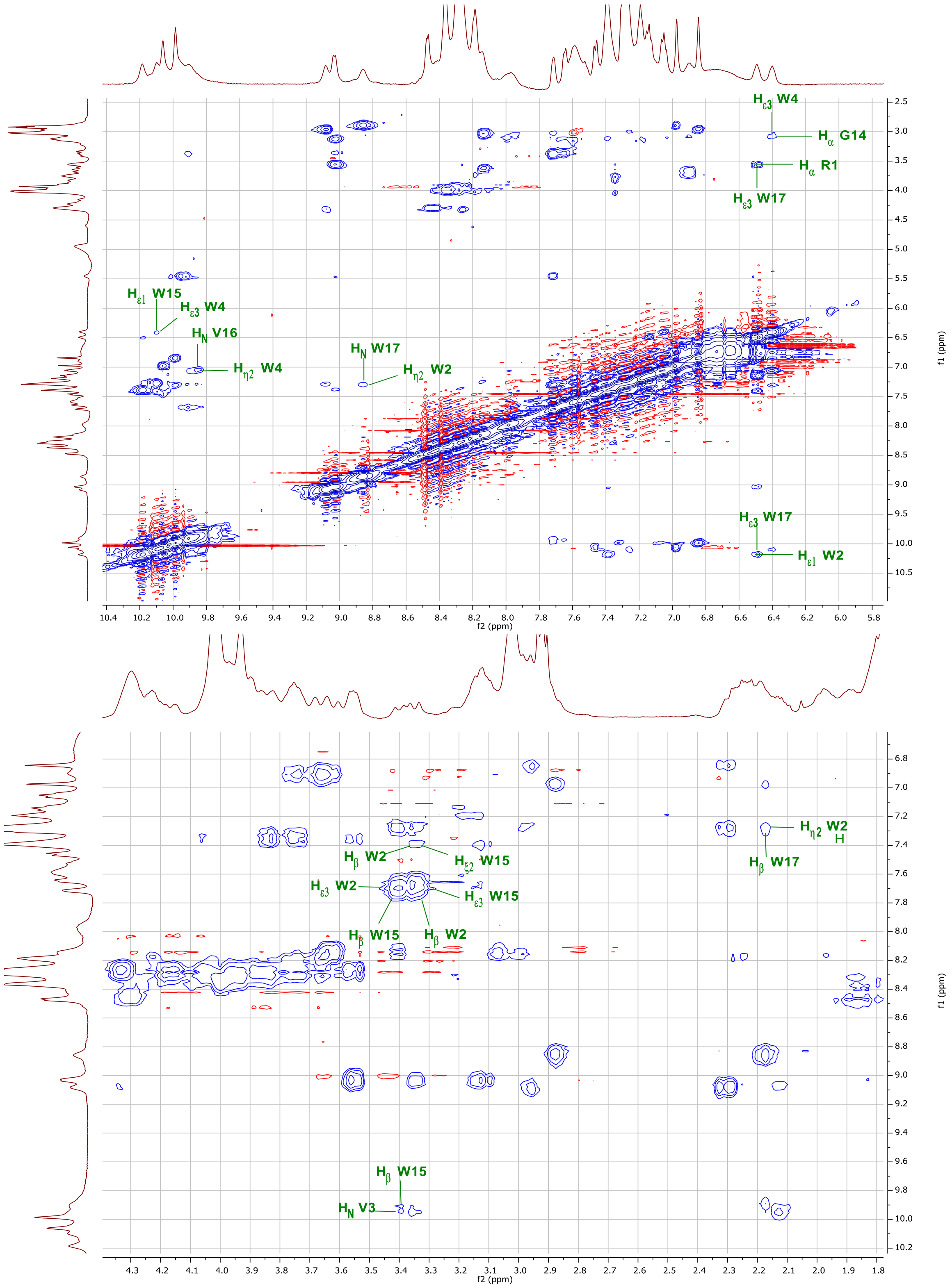

^1H NMR and ^1H ^{13}C-HSQC (500 MHz) of **1b** in water/DMSO-d_6 (90:10, vol/vol).

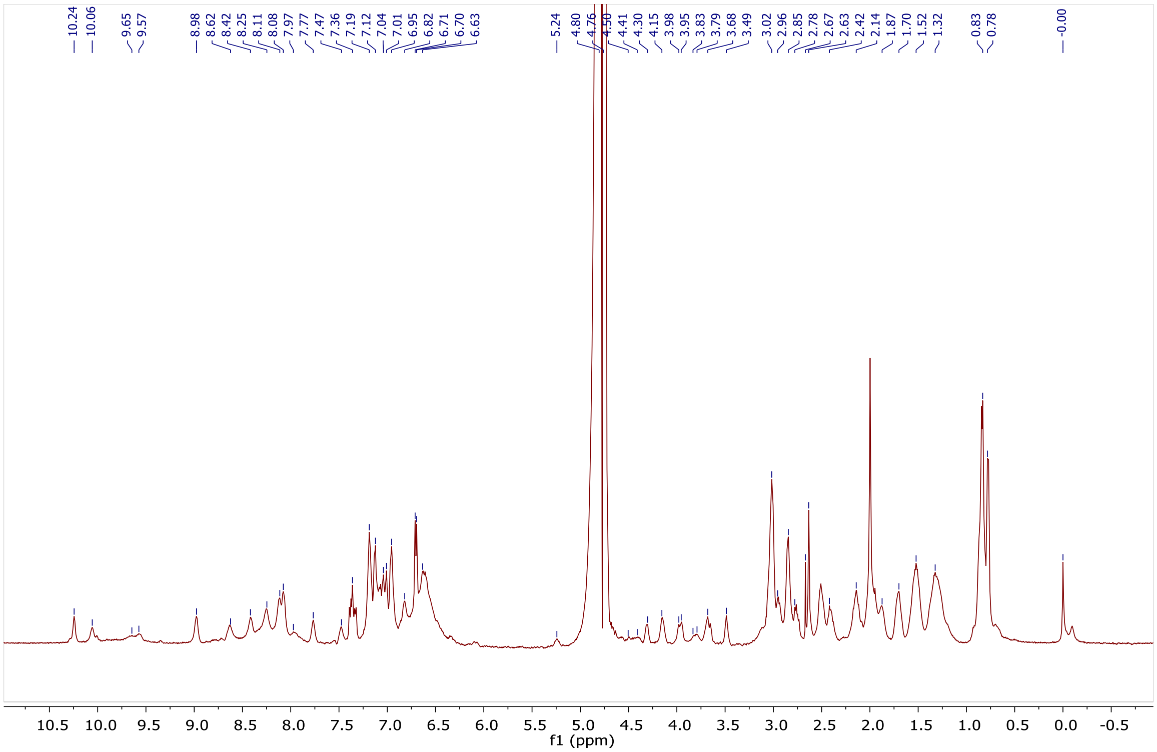

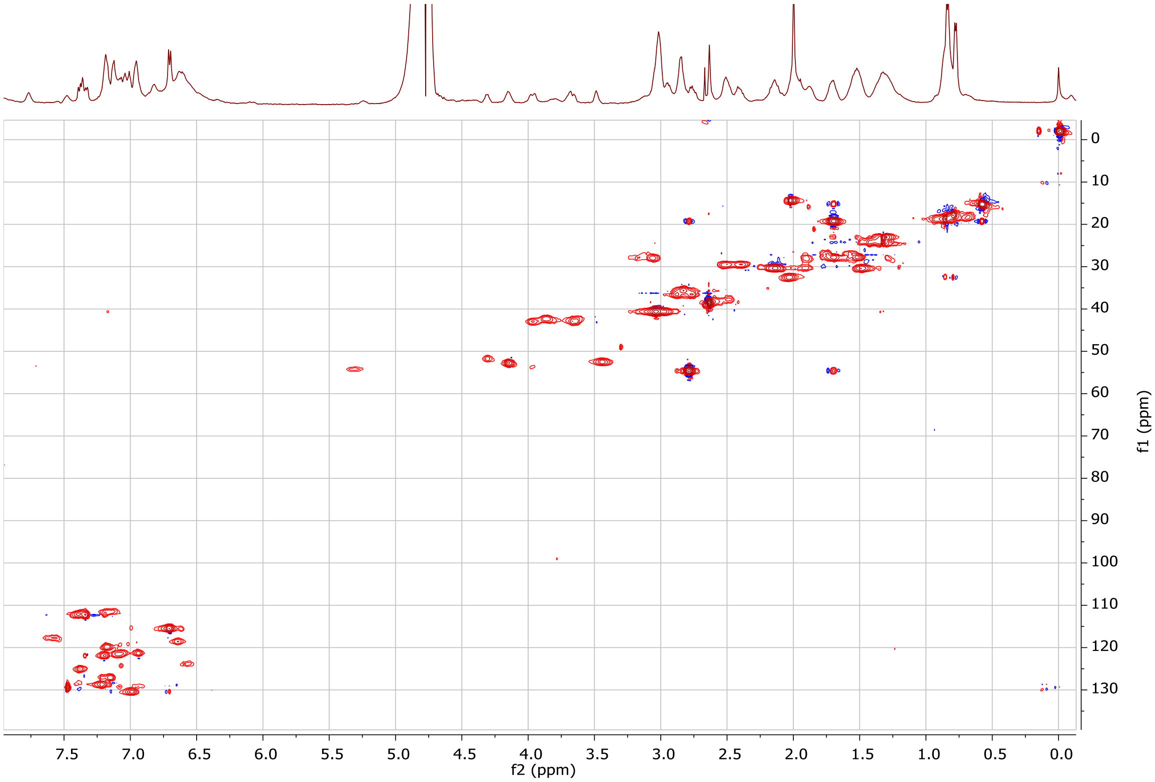

TOCSY and NOESY (500 MHz) of **1b** in water/DMSO-d_6 (90:10, vol/vol).

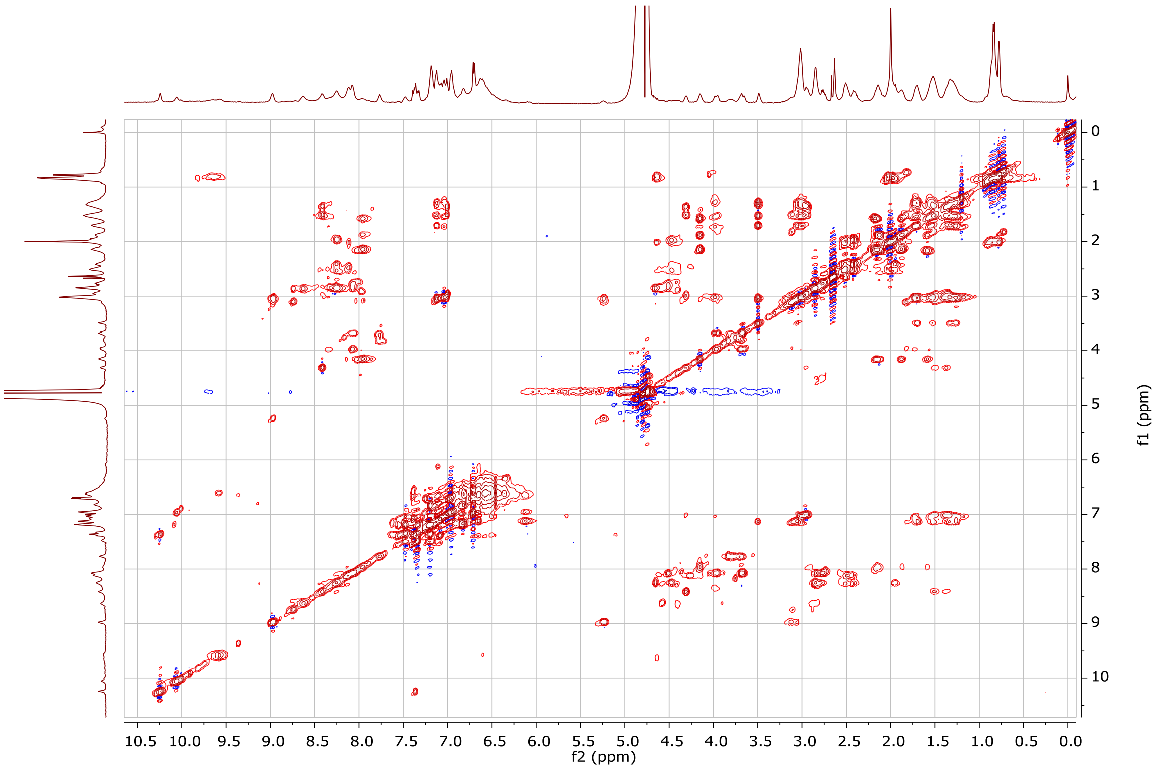

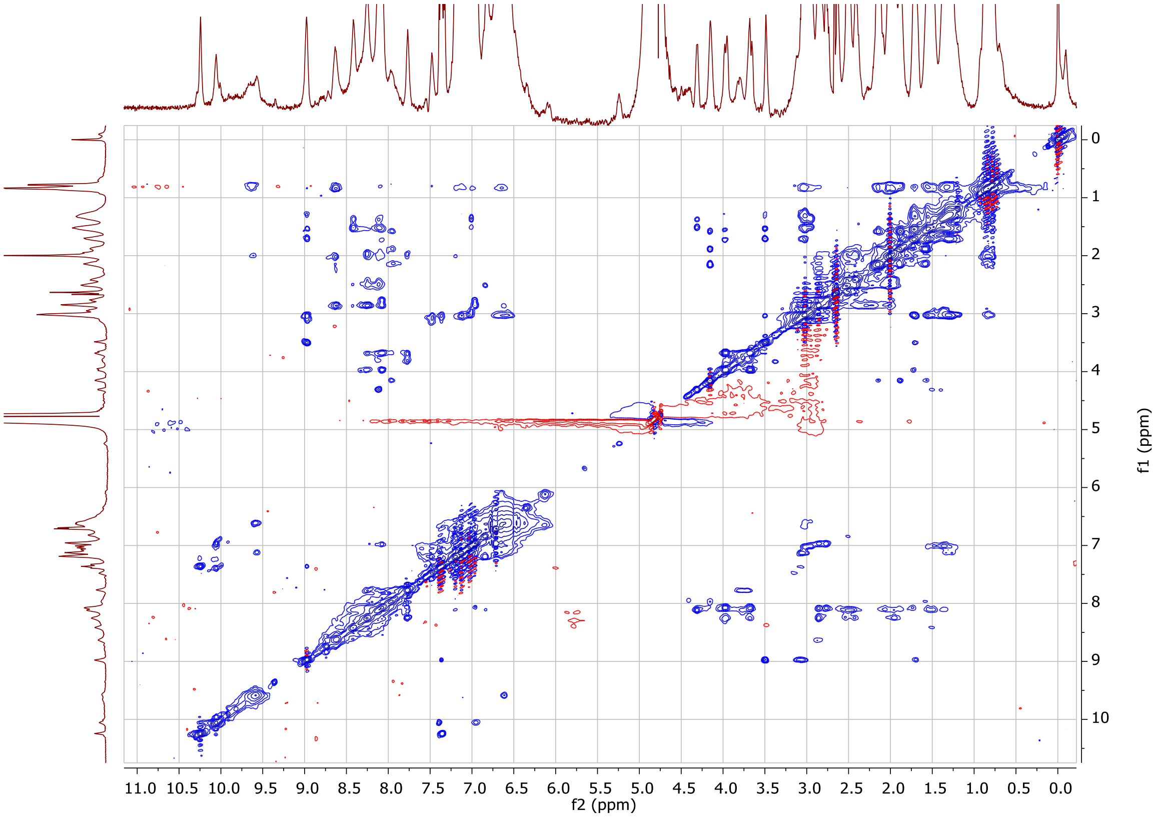

TOCSY and NOESY Overlay on the fingerprint region (500 MHz) of **1b** in water/DMSO-d_6 (90:10, vol/vol).

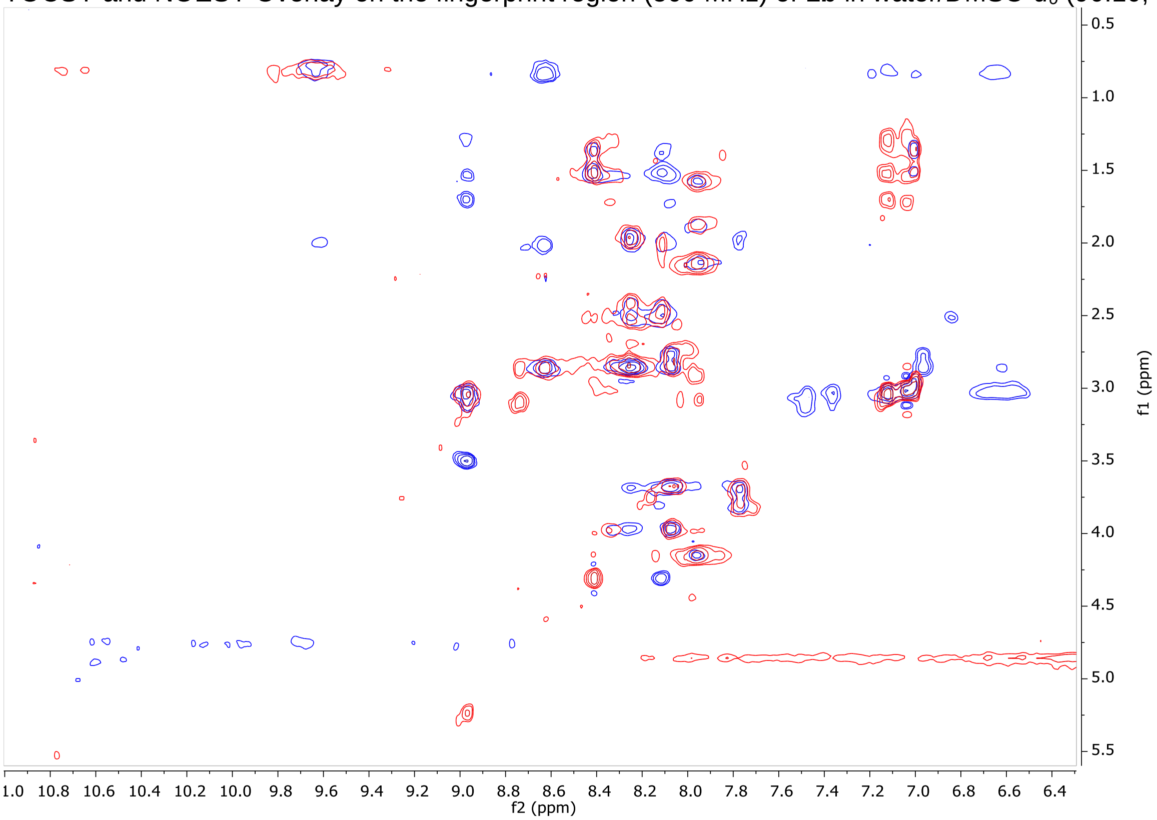

Selected NOESY correlations (500 MHz) of **1b** in water/DMSO-d_6 (90:10, vol/vol).

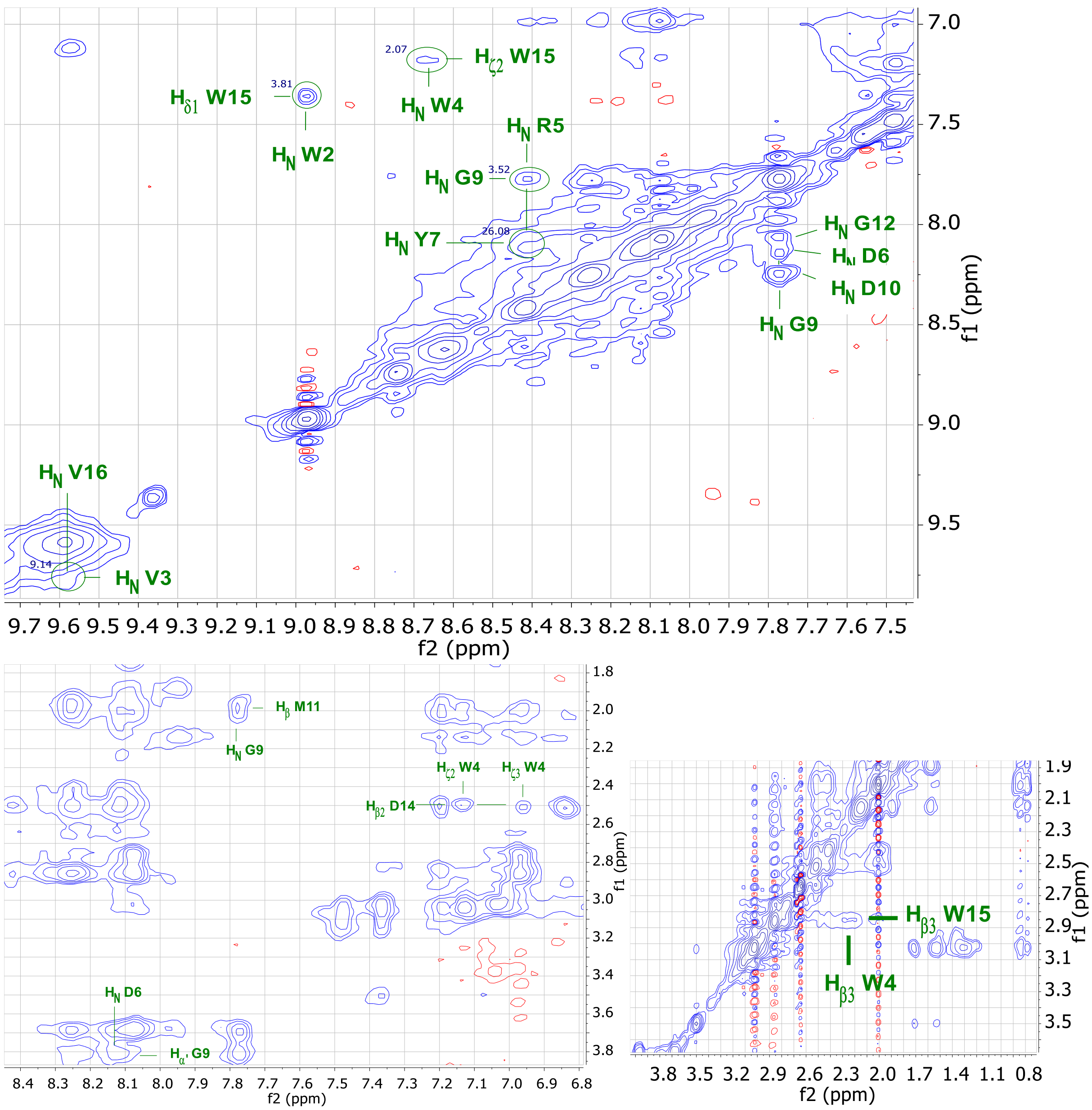

^{1}H NMR (500 MHz) of **2a** in water/DMSO-d_6 (90:10, vol/vol).

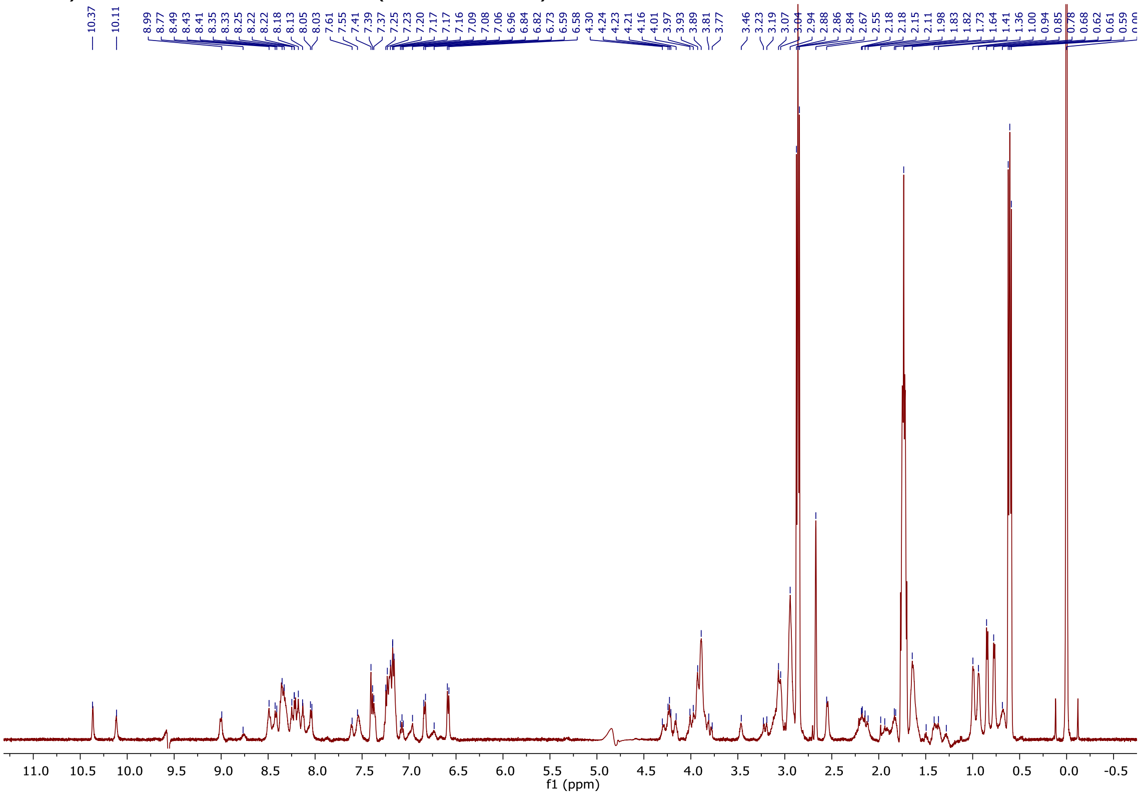

^{1}H ^{13}C-HSQC (500 MHz) of **2a** in water/DMSO-d_6 (90:10, vol/vol).

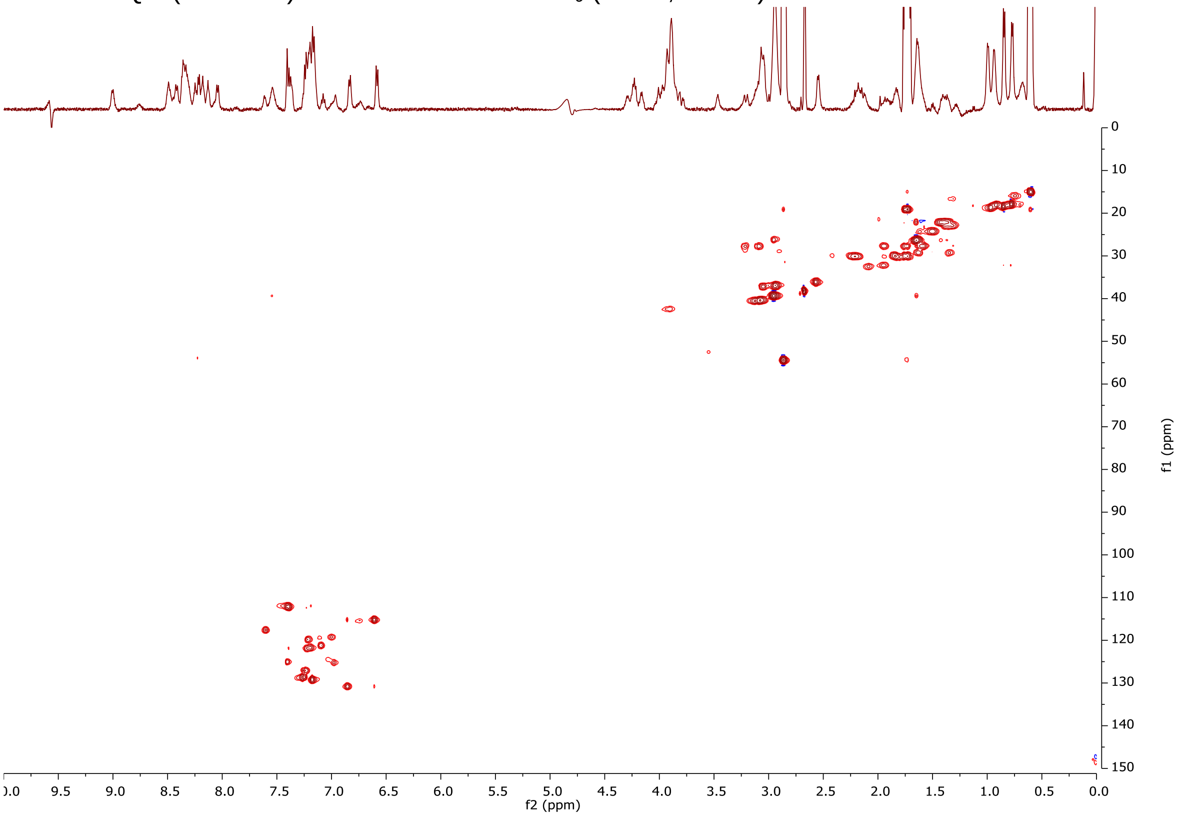

TOCSY and NOESY (500 MHz) of **2a** in water/DMSO-d_6 (90:10, vol/vol).

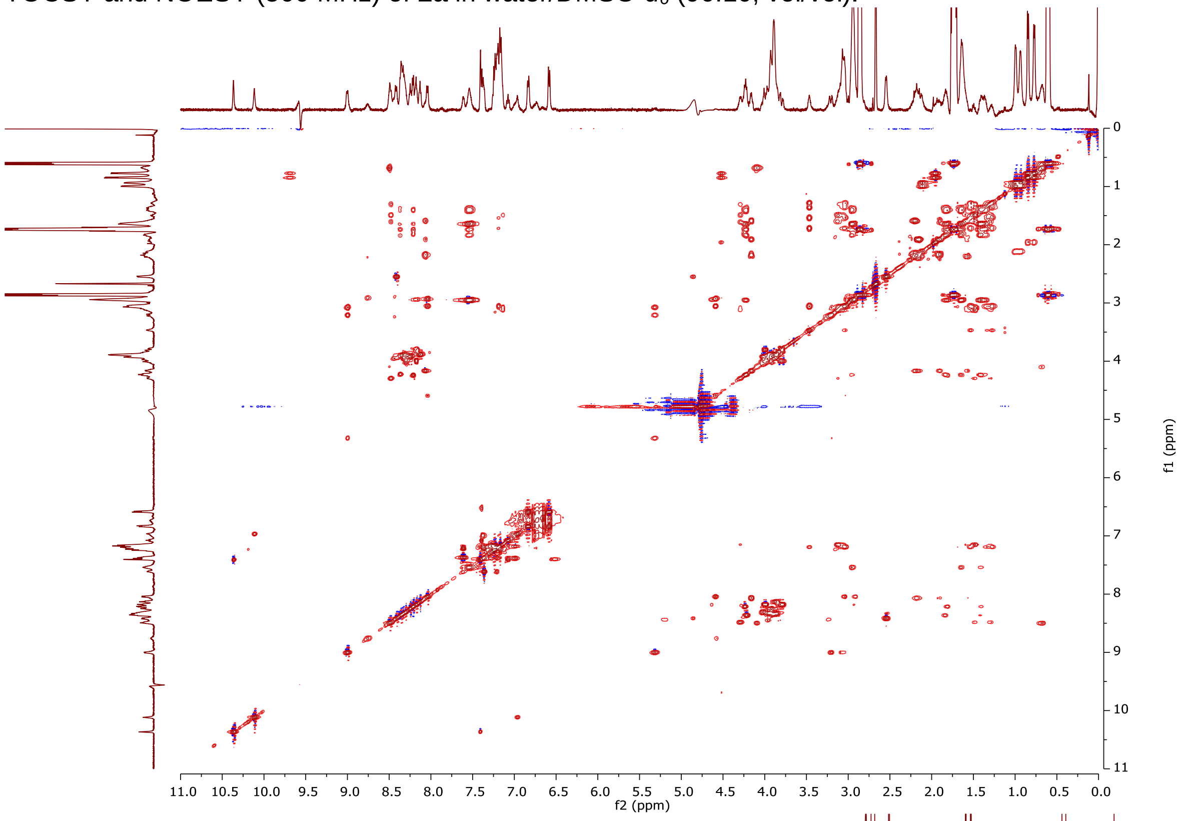

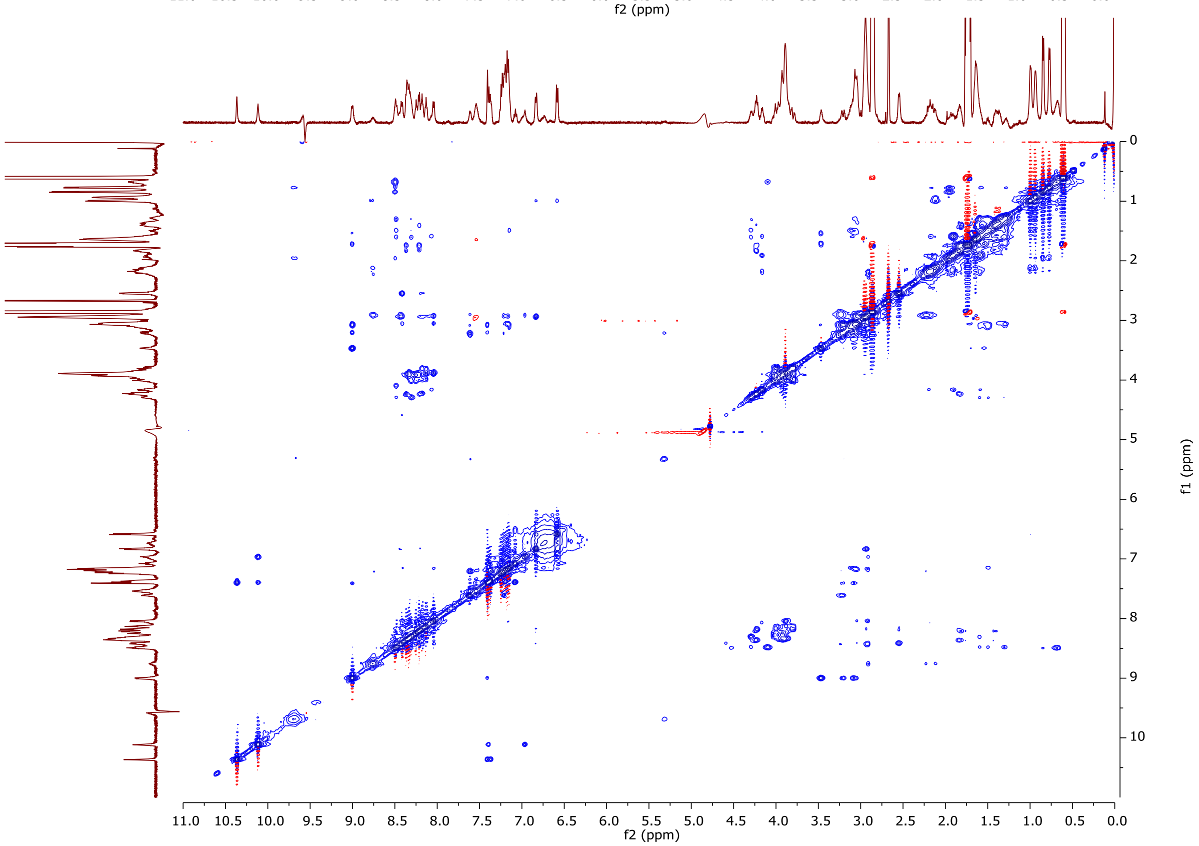

TOCSY and NOESY Overlay on the fingerprint region (500 MHz) of **2a** in water/DMSO-d_6 (90:10, vol/vol).

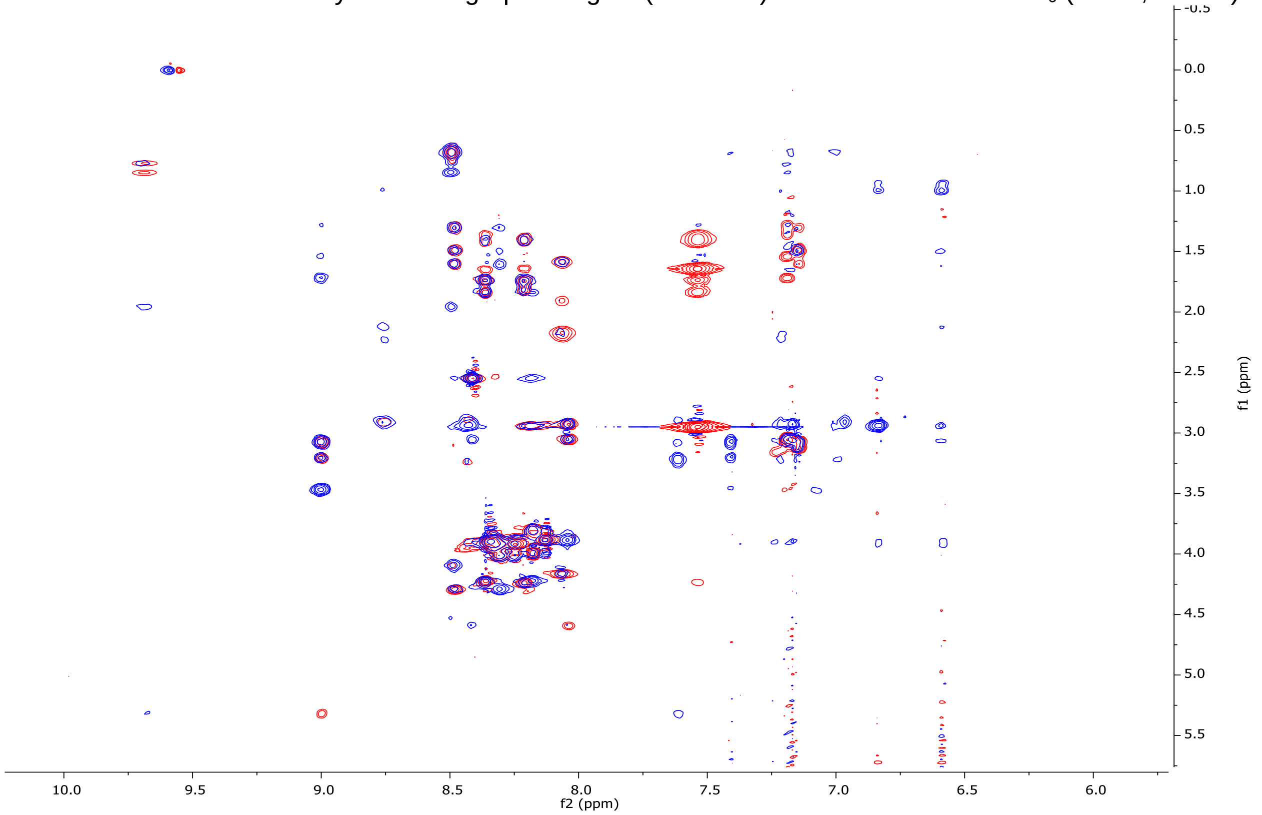

Selected NOESY correlations (500 MHz) of **2a** in water/DMSO-d_6 (90:10, vol/vol).

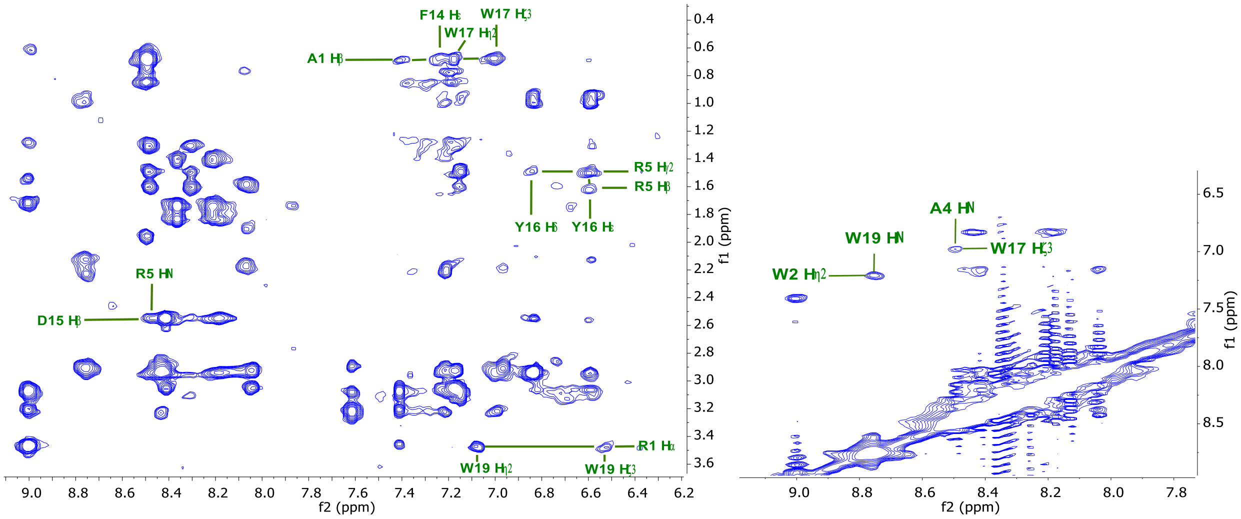

^{1}H NMR (500 MHz) of **2b** in water/DMSO-d_6 (90:10, vol/vol).

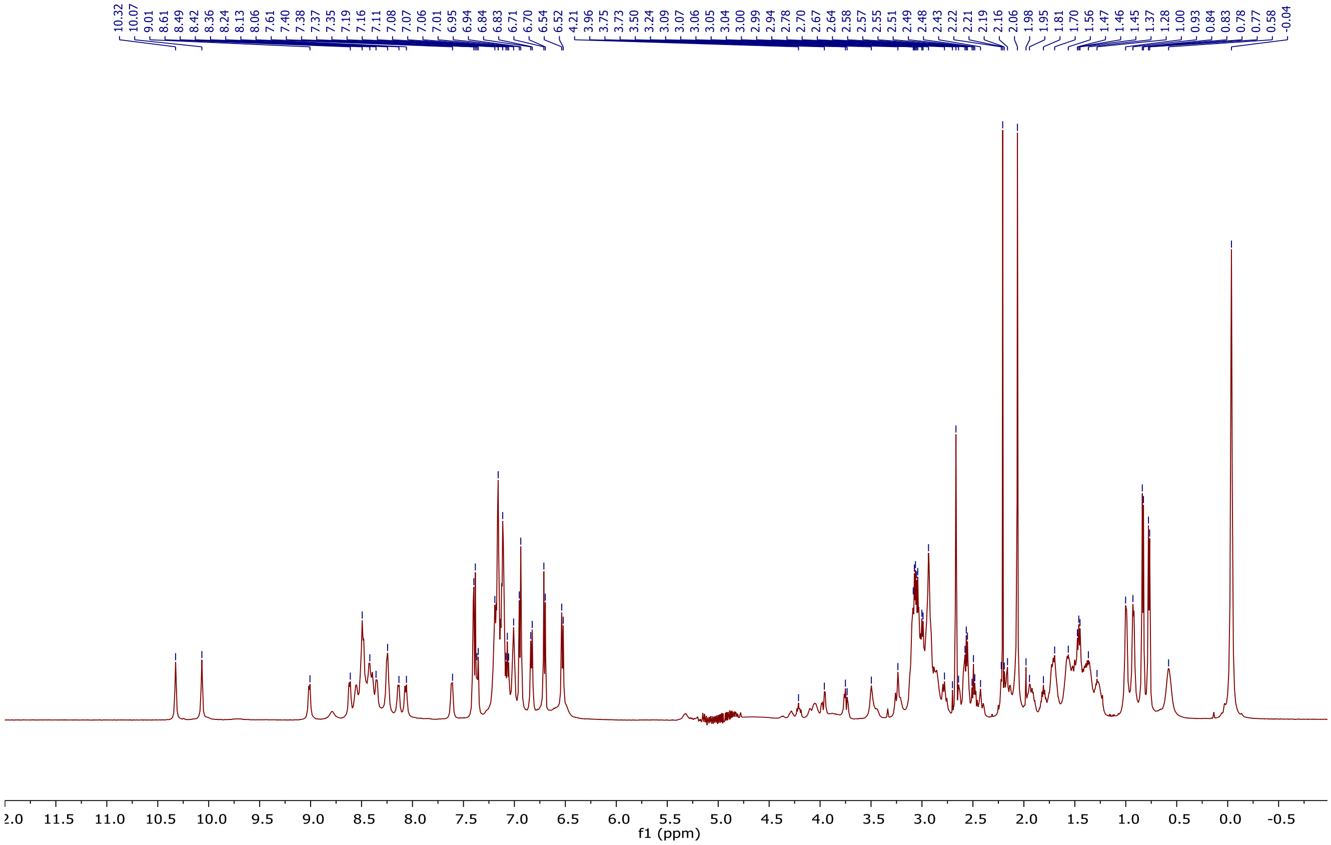

^{1}H ^{13}C-HSQC and ^{1}H ^{15}N-HSQC (500 MHz) of **2b** in water/DMSO-d_6 (90:10, vol/vol).

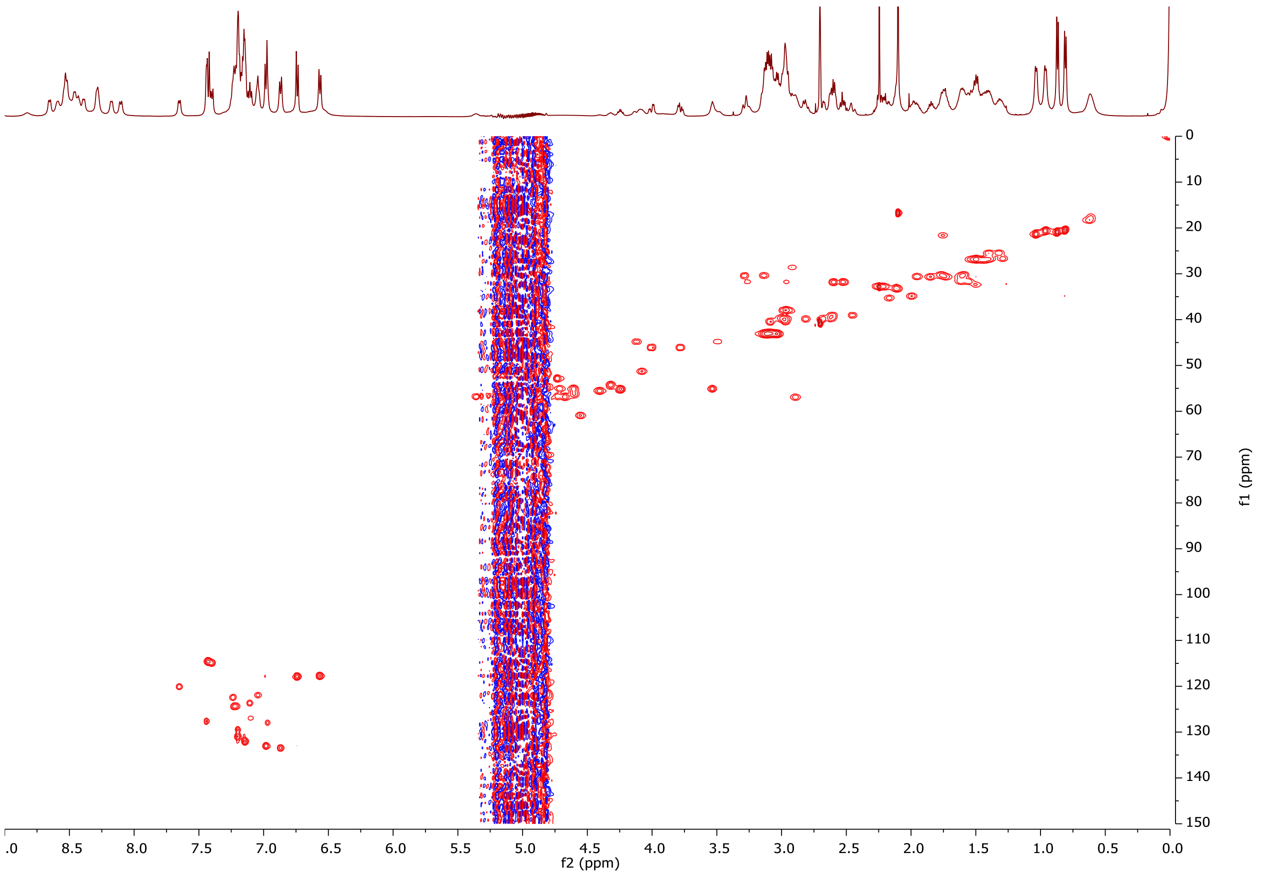

TOCSY and NOESY (500 MHz) of **2b** in water/DMSO-d_6 (90:10, vol/vol).

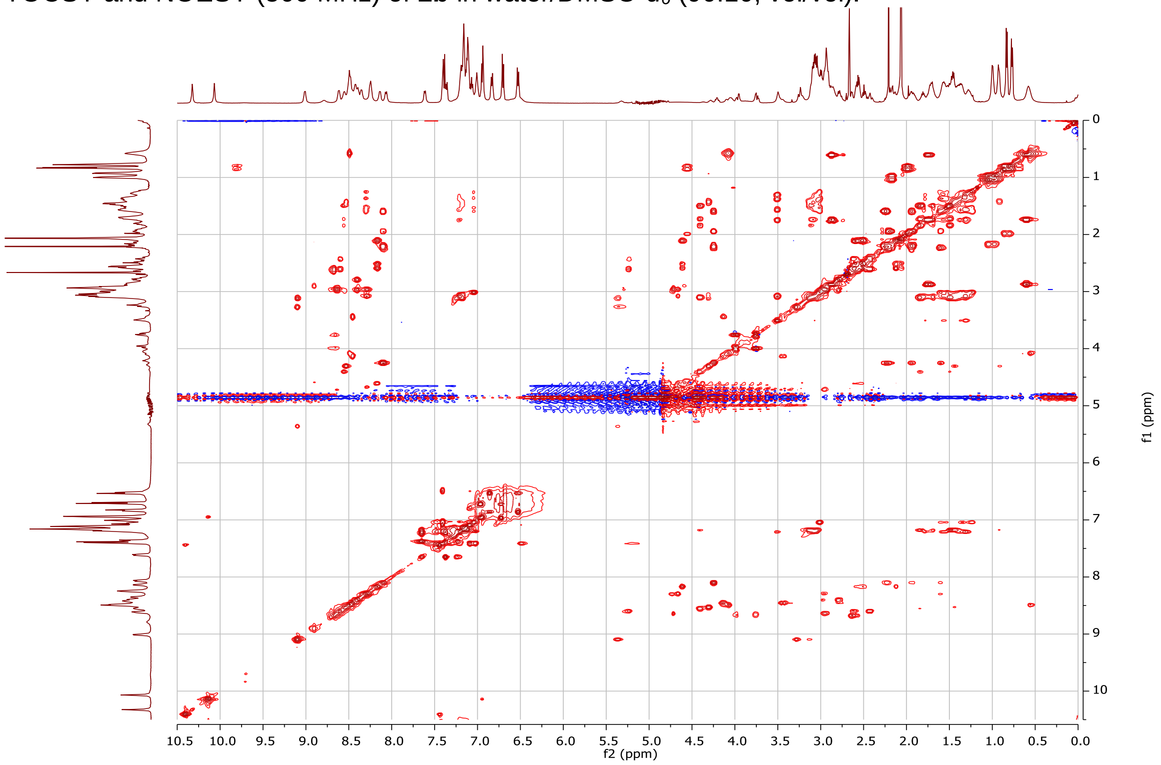

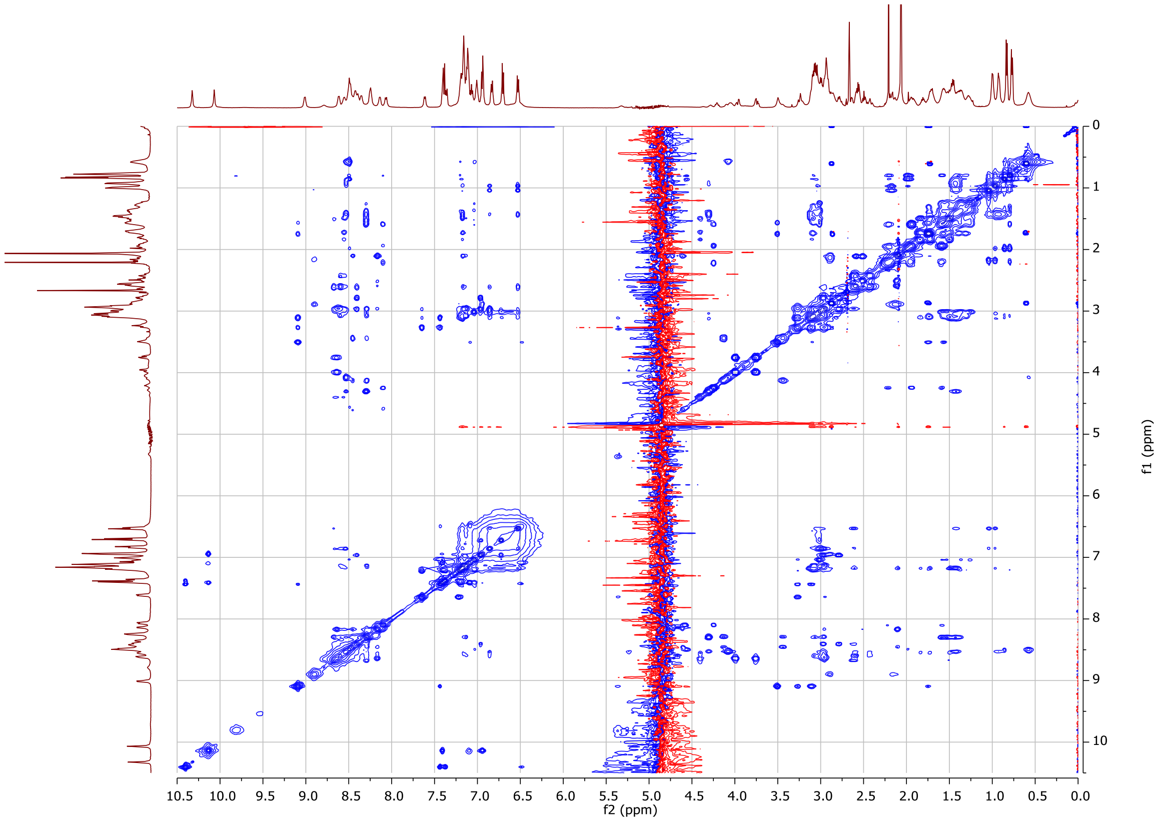

TOCSY and NOESY Overlay on the fingerprint region (500 MHz) of **2b** in water/DMSO-d_6 (90:10, vol/vol).

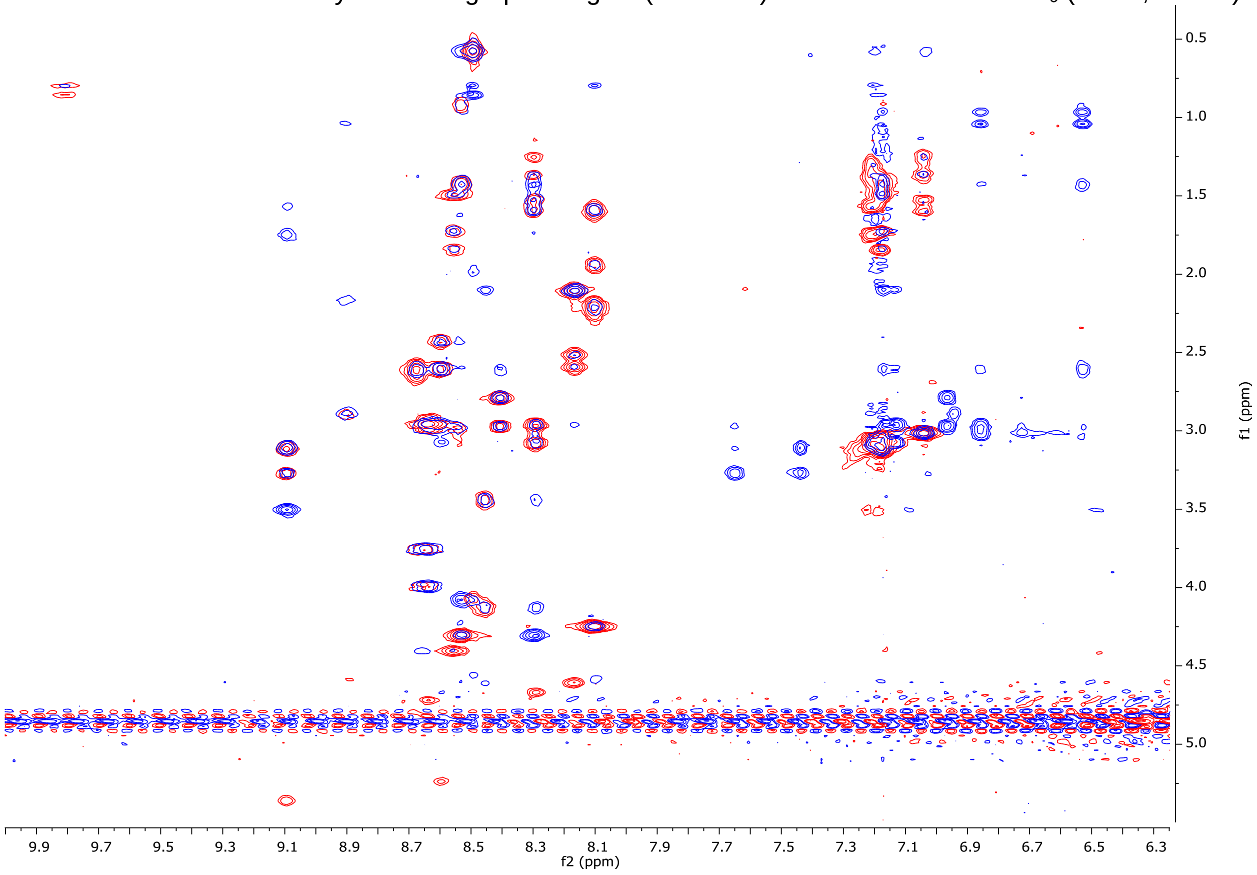

selected NOESY correlations (500 MHz) of **2b** in water/DMSO-d_6 (90:10, vol/vol).

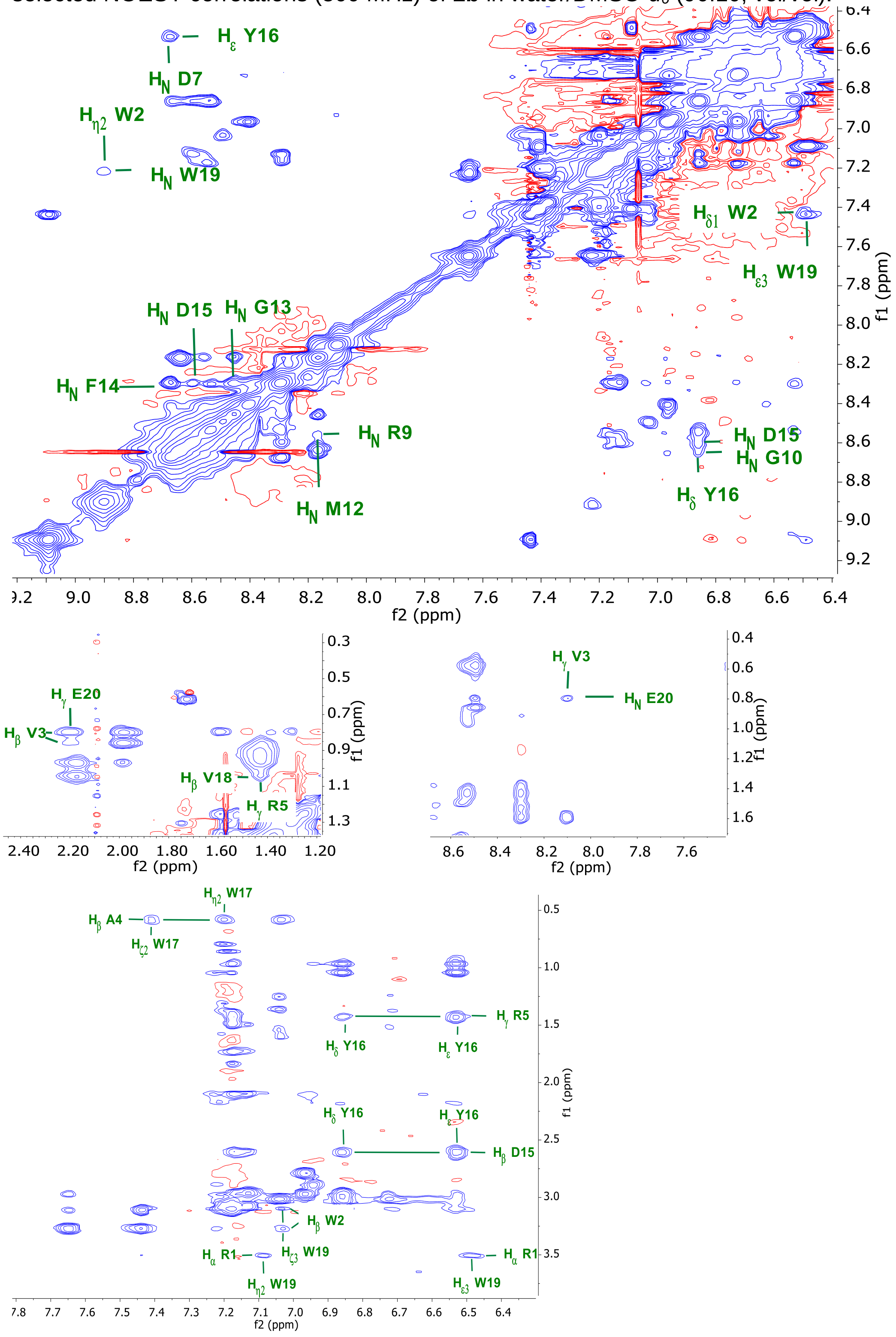

References

[SI-1] Laskowski, R. A.; Swindells, M. B. LigPlot+: Multiple Ligand–Protein Interaction Diagrams for Drug Discovery. *J. Chem. Inf. Model.* **2011**, *51*, 2778.

[SI-2] The PyMOL Molecular Graphics System, Version 2.0 Schrödinger, LLC.

[SI-3] Ribeiro, J., Ríos-Vera, C., Melo, F., Schüller, A. Calculation of accurate interatomic contact surface areas for the quantitative analysis of non-bonded molecular interactions. *Bioinformatics* **2019**, 35, 3499.

[SI-4] Lyskov S, Chou FC, Conchúir SÓ, Der BS, Drew K, Kuroda D, Xu J, Weitzner BD, Renfrew PD, Sripakdeevong P, Borgo B, Havranek JJ, Kuhlman B, Kortemme T, Bonneau R, Gray JJ, Das R., "Serverification of Molecular Modeling Applications: The Rosetta Online Server That Includes Everyone (ROSIE)". *PLoS One* **2013**, 8(5):e63906.

[SI-5] Kilambi KP, Gray JJ., "Rapid calculation of protein pKa values using Rosetta". *Biophysical Journal* **2012**, 103(3):587-595.

[SI-6] Greenfield, N. J. Using Circular Dichroism Collected as a Function of Temperature to Determine the Thermodynamics of Protein Unfolding and Binding Interactions. *Nat. Protoc.* **2006**, 1, 2527.

[SI-7] Richaud, A. D.; Zaghouani, M.; Zhao, G.; Wangpaichitr, M.; Savaraj, N.; Roche, S. P. *ChemBioChem* **2022**, 23, e202200449.

[SI-8] Hoare, S. R. J. Analyzing Kinetic Binding Data. In Assay Guidance Manual [Internet] Markossian, S., Grossman, A., Brimacombe, K., Eds.; Bethesda (MD): Eli Lilly & Company and the National Center for Advancing Translational Sciences, **2021**.

[SI-9] Wu, C.; Spector, S. A.; Theodoropoulos, G.; Nguyen, D. J. M.; Kim, E. Y.; Garcia, A.; Savaraj, N.; Lim, D. C.; Paul, A.; Feun, L. G.; Bickerdike, M.; Wangpaichitr, M. Dual inhibition of IDO1/TDO2 enhances anti-tumor immunity in platinum-resistant non-small cell lung cancer. *Cancer Metab.* **2023**, *11*, 7.

[SI-10] Kjaergaard, M.; Poulsen, F.M. Sequence correction of random coil chemical shifts: correlation between neighbor correction factors and changes in the Ramachandran distribution. *J. Biomol. NMR* **2011**, 50, 157.
